# Supplementary material for: A Meta-Analysis of Social and Contextual Correlates of Migrant Adaptation to Living in Receiving Societies
Source: Nat Commun. 2025 Dec 17;16:11231. doi: 10.1038/s41467-025-67468-z (PMC12715197; doi:10.1038/s41467-025-67468-z)
Supplement: Supplementary file 1 — Supplementary Information [file 41467_2025_67468_MOESM1_ESM.pdf]

Supplementary Information

For

A Meta-Analysis of Social and Contextual Correlates of Migrant Adaptation to Living in Receiving Societies

Kinga Bierwiaczonek[1,2,3*], Dinh H. Vu[3], Rongtian Tong[4], Mike W.-L. Cheung[5], Nora C.G. Benningstad[3], Evita van Duin[6], Karine Lindholm[3,7], Colleen Ward[8], Jonas R. Kunst[9]

[1] Leibniz Institute for Psychology (ZPID), Trier, Germany, [2] Trier University, Trier, Germany, [3]University of Oslo, Oslo, Norway, [4] University of Washington, Seattle, USA, [5] National University of Singapore, Singapore, Singapore, [6] Ghent University, Ghent, Belgium, [7] Norwegian Police University College, Oslo, Norway, [8] Victoria University of Wellington, Wellington, New Zealand, [9] BI Norwegian Business School, Oslo, Norway

* Corresponding author. Email: kmb@leibniz-psychology.org

Content links:

- Supplementary Methods
- Supplementary Note 1
- Supplementary Figure 1
- Supplementary Tables (also available in Excel via OSF for improved readability)
  - Supplementary Table 1
  - Supplementary Table 2
  - Supplementary Table 3
  - Supplementary Table 4
  - Supplementary Table 5
  - Supplementary Table 6
  - Supplementary Table 7
  - Supplementary Table 8
  - Supplementary Table 9
  - Supplementary Table 10
  - Supplementary Table 11
  - Supplementary Table 12
  - Supplementary Table 13
  - Supplementary Table 14
  - Supplementary Table 15
  - Supplementary Table 16
  - Supplementary Table 17
- Supplementary References

Supplementary Methods

Eligibility Criteria

Studies included in this meta-analysis are listed at the end of this document, [1-1005]. To be eligible for this meta-analysis had to fulfill the following criteria:

- An empirical quantitative study (cross-sectional, longitudinal, experimental)
- Conducted between 1988 (date of publication of the first paper deemed to be based on a theoretical framing compatible with the current meta-analysis[1006]) and the end of data collection
- Published or unpublished
- Available in English
- Include an eligible sample consisting of participants in international mobility (1st generation migrants, international students, expatriates and their families, refugees; see below for eligible and ineligible samples)
- Include at least one eligible measure of cross-cultural adaptation (overall, psychological, socio-cultural, occupational; see Section 3, Data items, for eligible measures)
- Include at least one eligible measure of antecedents of adaptation from any of the following groups: (a) stress-and-coping factors (acculturative stressors and resources, including family resources), (b) culture-learning factors (related to social learning and learning generalization), and (c) social-cognitive factors (related to intergroup perceptions; see Section 3, Data items, for eligible measures)
- Report at least one correlation between a measure of adaptation and a measure of adaptation antecedent OR other statistical information sufficient to estimate at least one such correlation.

Sample eligibility:

- *Include* studies with samples drawn from the following populations in international transitions: (1) expatriate employees (business expatriates, self-initiated expatriates) and expatriate families (spouses, children), (2) international students, (3) international 1st generation migrants (includes 1.5 generation), (4) refugees.

- *Exclude* other populations, such as tourists, repatriates (people coming back to their home country, including second and further generation repatriates), internal migrants (e.g., rural-to-urban), 2nd or further generation immigrants.
- *Exclude* samples selected based on specific mental health or physical health status (pregnant women, abuse survivors, torture survivors, HIV patients, cancer patients, diabetes patients, people in in-patient treatment centers, people with psychiatric disorders, etc.)

Examples of studies excluded based on the above criteria:

- Bakhtiari et al., 2018[1007]: Reason for exclusion: The sample consists of 84% second-generation migrants (not first generation)
- Gokuladas, 2022[1008]: Reason for exclusion: The outcome variable, satisfaction with school facilities, is not relevant (no eligible measures of cross-cultural adaptation)
- Gomes, 2020[1009]: Reason for exclusion: This study uses qualitative methods (not quantitative).

Information sources and search strategy

Studies dating from the period 1988-2014 were included from an existing database by Bierwiaczonek[1010,1011]. This database was adjusted to strictly match the inclusion criteria and coding scheme presented in the current protocol. To ensure that no studies from 1988-2014 were missed, a supplementary backward search was conducted by checking the reference lists of previous literature reviews: Bak-Klimek at al., 2015; Bender et al., 2019; Bhaskar-Shrinivas et al., 2005; Dimitrova et al., 2016; van der Laken et al., 2019; Wilson et al., 2019[1012-1018].

Studies dating from 2014 or later were identified using a new literature search. Search strategies were developed in collaboration with University of Oslo's library staff who has expertise in systematic review searching and is not associated with the project. The search was limited to the English language from the year 2014 onwards. We searched PsycINFO (OVID interface), Web of Science and Scopus on June 20, 2022; and ProQuest on June 24, 2022.

Below we present the search query in a format developed for PsycINFO. This query was developed by the project team and was peer-reviewed by a librarian. Once finalized, the query was adapted to the syntax and subject headings of the remaining databases.

1. ("cross-cultural" or "international" or "abroad" or "overseas" or "socio-cultural" or "intercultural" or "psychological").mp.
2. ("homesickness" or "psychosomatic" or "adjust*" or "adapt*" or "depress*" or "self-esteem" or "distress" or "well-being" or "satisfaction" or "stress").mp.
3. ("migrant*" or "immigrant*" or "international student*" or "expat*" or "refuge*" or "asylum seek*").mp.
4. 1 and 2 and 3
5. exp well being/ or exp health/ or exp life satisfaction/ or exp mental health/ or exp quality of life/ or exp social connectedness/ or exp social health/ or exp work life balance/
6. exp adjustment/
7. 5 or 6
8. exp refugees/ or exp human migration/ or exp asylum seeking/ or exp political asylum/
9. exp immigration/
10. exp migrant workers/ or exp migrant farm workers/ or exp foreign workers/
11. exp expatriates/ or exp foreign workers/
12. exp international students/
13. 8 or 9 or 10 or 11 or 12
14. 7 and 13
15. 4 or 14
16. limit 15 to (english language and yr="2014 -Current")

To ensure literature saturation, in addition to this core search strategy, a call for unpublished studies was sent to relevant scientific organizations: International Association of Cross-Cultural Psychology and International Academy of Intercultural Research. Call for unpublished studies was sent on May 9, 2023.

Screening and Coding

All hits from the databases covered by the core literature search (k = 16,639) were combined into one excel file. After a preliminary removal of duplicates and unrelated categories, the excel database was loaded to the ASReview Lab program for title and abstract screening. ASReview Lab is an open-source tool which uses active machine learning to maximise efficiency in the screening process by returning the most relevant records [1018]. Three members of the project team took turns reviewing titles and abstracts. All the abstracts marked as

irrelevant were then checked independently by the fourth team member. All discrepancies were discussed by the coder team. We used the following three criteria to determine when title and abstract screening could be stopped:

1. At least 33% of the database was screened: According to the software developers' estimates, 95% of the eligible studies will be found after screening between only 8% to 33% of the studies[1018].
2. The charts provided by ASReview Lab showed evidence that few relevant records can be added. Specifically, we analyzed two charts: the Progress chart and the Recall chart. The Progress chart depicts the number of relevant studies in the last 10 studies reviewed. The Recall chart compares the patterns of finding relevant records between ASReview's assisted screening and (theoretically) random manual screening. Both charts (available in project registration) were discussed by the project team and considered to indicate satisfactory saturation.
3. There were at least 30 consecutive irrelevant abstracts.

We stopped after screening abstracts of 5,697 reports (34.24% of the total records), with 33 consecutive irrelevant abstracts.

Full text screening and coding was conducted by trained members of the project team (a PhD student and research assistants). Each paper was screened and coded independently by two team members. All discrepancies were discussed and solved by the project team in a weekly meeting. The full coding scheme including the information extracted from each study is available in the project preregistration file (see https://osf.io/qc9h2/files/nb3rs). Coding categories were based on previous reviews by Bierwiaczonek[1010,1011].

Risk of Bias Assessment

To assess risk of bias of the cumulative estimate, we adapted the Joana Briggs Institute's (JBI) Critical Appraisal Tool for observational studies[1019]. Specifically, we applied the following criteria to each included study:

1. The criteria for inclusion in the study sample were clearly defined and reported, that is, the study included only first-generation migrants and provided a clear description of sample types (e.g., international students, expatriates, migrants, etc.).

2. The participants and the setting were described in detail, that is, the study provided a clear sample description, including gender/sex composition, mean age, mean length of stay in the host country, and clear details of the countries they migrated from and resided in.
3. The use of the same sample or a portion of the same sample for different reports was transparently communicated, that is, when multiple studies utilized the same sample or portion thereof to investigate different research questions, the study clearly acknowledge this overlap, particularly in subsequent publications stemming from the same sample.
4. The predictor variables were measured in a valid and reliable way, that is, the predictor measures were clearly described, Cronbach's alpha (if applicable) was ≥ 0.70, and no other issues were captured during the coding process (e.g., unclear scale direction).
5. The outcome variables were measured in a valid and reliable way, that is, the outcome measures were clearly described, Cronbach's alpha (if applicable) was ≥ 0.70, and no other issues are captured during the coding process (e.g., unclear scale direction).
6. Confounding bias was accounted for, and no potential item overlap (common method bias) existed between predictor and outcome measures.
7. Appropriate statistical analyses were reported, that is, bivariate correlations between predictor and outcome variables were available (either in the main paper, supplementary materials, or could be directly calculated from publicly available datasets).

Effect size conversion

Whenever a primary study did not report Pearson's correlation r but reported other measures of association, we converted the available measures following one of the following three approaches:

- We calculated Pearson's rs and the corresponding variances from t-statistics using Lipsey and Wilson's approach[1020]. We either used the reported t-statistics directly, or, if not reported, we calculated t using linear regression coefficients (unstandardized b, standard error, confidence interval, or p-value; standardized beta, number of predictors)[1020,1021].
- If one of the variables of interest was dichotomized, and the study provided sufficient information to calculate the standardized mean difference d between two relevant groups (e.g., means, standard deviations and sample sizes for each group; or unadjusted odds ratios), we estimated biserial correlations

and the corresponding variances from standardized mean difference *d* between two groups, following Jacobs and Viechtbauer[1022], and Pustejovsky[1023].

- If both variables of interest were dichotomized and the study reported enough information to construct a 2x2 contingency table, we estimated tetrachoric correlations and the corresponding variance following Viechtbauer[1024].

Supplementary Note 1

Methodological issues detected in the meta-analyzed literature

Please note that the below numbers are based on the full ACCA dataset (1,190 studies). Please see Vu et al.[1022] for a detailed discussion.

Reporting practices:

- **Failure to report essential demographic details (e.g., age, sex/gender, country of origin).**

  Of the included studies, 98.66% clearly report both the migrant generation and subgroup (e.g., migrants, international students, refugees, expats). However, 11.01% do not report the mean age of participants, 3.95% omit sex/gender information, 41.6% fail to include the mean length of stay, and 0.25% do not provide information about participants' countries of origin or destination. In other words, nearly half of the studies lack at least one essential demographic detail.
- **Omission of information on the reliability and validity of variables used in analyses**.

  7.28% of the included studies were flagged for having biased predictor measures and 4.57% for having biased outcome measures (e.g., Cronbach's alpha lower than .70 or noted issues with reliability or validity). 65.65% of studies lacked sufficient information to assess the reliability and/or validity of predictor measures, and 32.17% of outcome measures (e.g., unreported Cronbach's alpha, unreported scale items for face-validity evaluation, the use of one-item scales, lack of clarity regarding scale direction).
- **Failure to report other basic statistical information (i.e., bivariate correlations):** 43.53% of studies
- **Scarcity of publicly available datasets, indicating a lack of open science practices.**

Measurement:

- **Conceptual overlap between measures:**

  Common method bias (i.e., semantic and conceptual overlap between measures that are theoretically adaptation outcomes, e.g., depression, culture shock; and measures that assess adaptation antecedents, e.g., acculturative stress scales, loneliness) can be found in at least 15% of the included studies. Additionally, acculturative stressor measures include other concepts that are used as

predictors of adaptation (e.g., subscales for perceived discrimination, language barrier) that are often combined into one composite scale.

Research designs:

- **Lack of designs allowing for causal inference.** 88.82% of included studies are cross-sectional observational studies, 10.76% are longitudinal, and 0.42% are experimental.

Resulting biases:

We conducted risk of bias analyses including all issues reported above under *Reporting Practices* (see Supplementary Table 13, criteria ROB1, ROB2, ROB3, ROB7, ROB8) and the common method bias reported above under Measurement (ROB4). These analyses found no clear evidence of bias in terms of results, that is, the effect size and heterogeneity showed no meaningful change after biased studies were removed from analysis. However, additional analyses reported by Vu et al. (*in press*) suggested that confounding due to common method bias (ROB4), but not other biases, led to inflated effect sizes.

Supplementary Figure 1.

Funnel plots of the main categories of correlates related to culture learning (cultural distance, exposure to social groups) and stress-and-coping (perceived social resources, stressors) with psychological and socio-cultural adaptation. Primary effects in correlation units (X axis) are plotted against their standard error (Y axis). The number of effects is noted on the X axis of each figure.

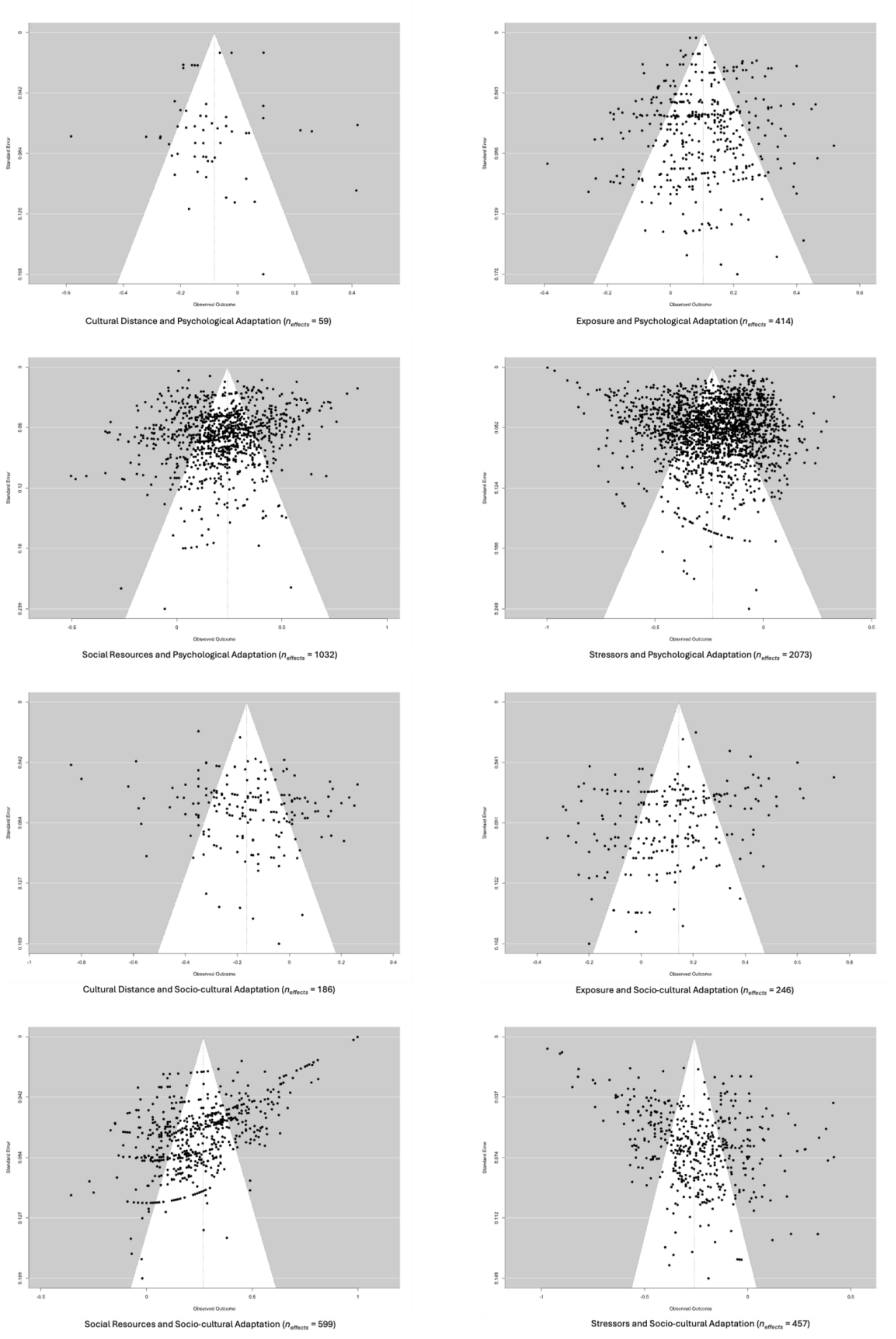

Supplementary Table 1.

Meta-regressions comparing the absolute values of overall cumulative estimates from the four main categories of predictors. A significant result (i.e., *p(r)* < .050) means that the average effect for a given category differs significantly in size from the reference; a non-significant result indicates a lack of significant difference.

Outcome	Predictor	*N*	*k* studies	*n* effects	*r*	*p(r)*	95% CI	*QE*	*df(QE)*	*p(QE)*	*QM*	*df(QM)*	*p(QM)*	τ^2 level 3	τ^2 level 2	Pseudo R^2
**Socio-cultural adaptation**		80,277	360	1,488				116,960	1,484	<.001	86	3	<.001	.01	.02	6.19%
	Stressors (reference)				.27	<.001	[.25, .29]									
	Cultural Distance				-.10	<.001	[-.14, -.07]									
	Exposure				-.12	<.001	[-.15, -.09]									
	Social Resources				-.01	.663	[-.03, .02]									
**Psychological adaptation**		491,260	910	3,578				677,310	3,574	<.001	133	3	<.001	.01	.02	5.50%
	Stressors (reference)				.24	<.001	[.22, .25]									
	Cultural Distance				-.13	<.001	[-.18, -.09]									
	Exposure				-.10	<.001	[-.12, -.08]									
	Social Resources				-.01	.378	[-.02, .01]									

Supplementary Table 2.

Subset analyses of main categories of correlates of adaptation by migrant population for psychological and socio-cultural adaptation. Effects reported in the *r* column refer to the average effect size within each sub-category.

Correlate	Outcome	Predictor	*N*	*k* studies	*n* effects	*r*	*p(r)*	95% CI	90% PI	*Q*	*p(Q)*	τ^2 level 3	τ^2 level 2	I^2 level 3	I^2 level 2	I^2 Total
**Stressors**	**Socio-cultural adaptation**	Migrants	9,489	39	72	-.27	<.001	[-.33, -.21]	[-.61, .07]	5,617	<.001	.02	.02	38.83%	56.24%	95.07%
		Students	26,001	91	196	-.29	<.001	[-.33, -.25]	[-.65, .07]	10,083	<.001	.02	.02	46.38%	49.28%	95.66%
		Refugees	1,557	6	10	-.23	.003	[-.37, -.08]	[-.63, .18]	191	<.001	.00	.05	0.00%	95.29%	95.29%
		Expats	13,950	69	171	-.22	<.001	[-.26, -.18]	[-.51, .07]	1,818	<.001	.01	.02	35.27%	53.33%	88.60%
	**Psychological adaptation**	Migrants	333,875	355	1,061	-.22	<.001	[-.23, -.20]	[-.49, .06]	499,944	<.001	.01	.02	39.77%	57.87%	97.64%
		Students	69,498	210	606	-.26	<.001	[-.29, -.24]	[-.55, .02]	11,685	<.001	.01	.01	46.54%	45.84%	92.38%
		Refugees	50,563	121	345	-.24	<.001	[-.27, -.21]	[-.53, .05]	30,103	<.001	.02	.01	51.77%	43.27%	95.04%
		Expats	4,898	21	36	-.19	<.001	[-.27, -.11]	[-.48, .10]	278	<.001	.03	.00	75.29%	11.40%	86.69%
**Social resources**	**Socio-cultural adaptation**	Migrants	6,526	29	70	.24	<.001	[.16, .31]	[-.13, .60]	7,878	<.001	.03	.02	63.76%	32.55%	96.31%
		Students	20,548	64	164	.29	<.001	[.25, .33]	[-.02, .61]	34,561	<.001	.02	.02	44.67%	52.20%	96.86%
		Refugees	739	4	9	.18	.002	[.06, .30]	[-.04, .40]	33	<.001	.01	.01	44.00%	29.70%	73.70%
		Expats	16,335	79	349	.26	<.001	[.23, .30]	[-.02, .55]	8,504	<.001	.02	.01	62.01%	28.45%	90.46%
	**Psychological adaptation**	Migrants	150,211	154	395	.25	<.001	[.22, .27]	[-.04, .53]	11,012	<.001	.02	.01	64.64%	31.19%	95.83%
		Students	42,206	143	382	.25	<.001	[.23, .28]	[-.02, .52]	4,320	<.001	.01	.01	49.76%	40.33%	90.10%
		Refugees	23,473	65	140	.20	<.001	[.16, .24]	[-.07, .48]	1,632	<.001	.02	.01	66.37%	24.71%	91.07%
		Expats	5,595	32	97	.22	<.001	[.15, .28]	[-.16, .59]	1,068	<.001	.02	.04	28.04%	62.63%	90.68%
**Exposure**	**Socio-cultural adaptation**	Migrants	1,367	5	6	.22	.005	[.06, .37]	[-.11, .54]	61	<.001	.00	.03	0.00%	91.18%	91.18%
		Students	12,437	51	149	.14	<.001	[.10, .19]	[-.17, .46]	1,424	<.001	.01	.02	36.99%	52.36%	89.34%
		Refugees	707	3	6	.21	<.001	[.13, .29]	[.06, .36]	14	.016	.00	.01	0.00%	64.14%	64.14%
		Expats	2,622	18	73	.15	<.001	[.08, .21]	[-.14, .43]	391	<.001	.01	.02	36.97%	44.58%	81.54%
	**Psychological adaptation**	Migrants	114,180	56	147	.09	<.001	[.06, .12]	[-.10, .28]	1,505	<.001	.01	.00	62.39%	32.23%	94.61%
		Students	17,211	56	197	.12	<.001	[.09, .15]	[-.08, .33]	820	<.001	.01	.01	27.86%	51.73%	79.59%
		Refugees	13,453	15	27	.08	<.001	[.04, .11]	[-.01, .17]	58	<.001	.00	.00	58.34%	12.50%	70.84%
		Expats	1,622	10	22	.12	.011	[.03, .21]	[-.13, .37]	76	<.001	.01	.01	52.14%	22.60%	74.74%
**Cultural distance**	**Socio-cultural adaptation**	Migrants	3,521	11	13	-.13	.067	[-.27, .01]	[-.53, .27]	243	<.001	.03	.02	51.89%	42.03%	93.92%
		Students	4,603	22	26	-.25	<.001	[-.34, -.16]	[-.58, .08]	232	<.001	.04	.00	82.97%	6.81%	89.78%
		Expats	12,252	61	143	-.14	<.001	[-.18, -.11]	[-.42, .13]	1,083	<.001	.01	.02	33.38%	52.27%	85.66%
	**Psychological adaptation**	Migrants	7,593	5	11	-.02	.814	[-.17, .13]	[-.32, .29]	269	<.001	.03	.00	90.10%	8.32%	98.41%
		Students	6,462	20	33	-.15	<.001	[-.22, -.09]	[-.39, .08]	120	<.001	.02	.00	86.60%	0.00%	86.60%
		Expats	1,946	14	15	.01	.879	[-.09, .11]	[-.29, .30]	73	<.001	.03	.00	80.51%	0.00%	80.51%

Supplementary Table 3.

Meta-regressions with migrant population as categorical predictor of the effect size, per broad category of correlates of adaptation and per outcome (socio-cultural and psychological adaptation). A significant result (i.e., *p(r)* < .05) means that the average effect for a given category differs significantly in size from the reference; a non-significant result indicates a lack of significant difference.

Correlate	Outcome	Predictor	*N*	*k* studies	*n* effects	*B*	*p(B)*	95% CI	*QE*	*df(QE)*	*p(QE)*	*QM*	*df(QM)*	*p(QM)*	τ^2 lv 3	τ^2 lv 2	Pseudo R^2
**Stressors**																	
	**Socio-cultural adaptation**		49,574	210	457				17,770	452	<.001	6	4	.175	.02	.02	0.67%
		Migrants (reference)				-.27	<.001	[-.33, -.21]									
		Refugees				.03	.763	[-.14, .19]									
		Expats				.05	.177	[-.02, .12]									
		Students				-.02	.550	[-.09, .05]									
		Unspecific				.03	.724	[-.15, .21]									
	**Psychological adaptation**		437,656	719	2,073				542,429	2,068	<.001	14	4	.007	.01	.02	1.32%
		Migrants (reference)				-.22	<.001	[-.23, -.20]									
		Refugees				-.02	.202	[-.05, .01]									
		Expats				.02	.630	[-.06, .09]									
		Students				-.05	<.001	[-.07, -.02]									
		Unspecific				-.04	.426	[-.13, .05]									
**Social resources**																	
	**Socio-cultural adaptation**		42,818	179	598				50,990	593	<.001	4	4	.453	.02	.01	0%
		Migrants (reference)				.24	<.001	[.17, .31]									
		Refugees				-.06	.541	[-.24, .13]									
		Expats				.02	.567	[-.05, .10]									
		Students				.06	.155	[-.02, .14]									
		Unspecific				-.02	.834	[-.24, .20]									
	**Psychological adaptation**		217,893	399	1,029				18,156	1,024	<.001	5	4	.293	.02	.01	0.07%
		Migrants (reference)				.25	<.001	[.22, .27]									
		Refugees				-.05	.062	[-.09, .00]									
		Expats				-.02	.617	[-.08, .05]									
		Students				.01	.753	[-.03, .04]									
		Unspecific				.02	.748	[-.12, .17]									

Supplementary Table 3 (cont.)

Correlate	Outcome	Predictor	*N*	*k* studies	*n* effects	*B*	*p(B)*	95% CI	*QE*	*df(QE)*	*p(QE)*	*QM*	*df(QM)*	*p(QM)*	τ^2 lv 3	τ^2 lv 2	Pseudo R^2
**Exposure**																	
	**Socio-cultural adaptation**		17,082	79	246				1,927	241	<.001	4	4	.463	.01	.02	0%
		Migrants (reference)				.21	.011	[.05, .37]									
		Refugees				-.01	.940	[-.25, .24]									
		Expats				-.06	.480	[-.24, .11]									
		Students				-.06	.454	[-.23, .10]									
		Unspecific				-.23	.078	[-.49, .03]									
	**Psychological adaptation**		145,119	144	414				2,506	409	<.001	5	4	.341	.01	.01	0%
		Migrants (reference)				.09	<.001	[.06, .12]									
		Refugees				-.01	.682	[-.08, .05]									
		Expats				.03	.507	[-.05, .11]									
		Students				.03	.112	[-.01, .07]									
		Unspecific				-.03	.529	[-.12, .06]									
**Cultural distance**																	
	**Socio-cultural adaptation**		18,824	94	182				1,558	179	<.001	6	2	.047	.01	.02	1.71%
		Migrants (reference)				-.13	.021	[-.24, -.02]									
		Expats				-.02	.784	[-.13, .10]									
		Students				-.12	.074	[-.26, .01]									
	**Psychological adaptation**		15,362	39	59				462	56	<.001	8	2	.017	.02	.00	17.94%
		Migrants (reference)				-.02	.803	[-.16, .12]									
		Expats				.03	.758	[-.14, .19]									
		Students				-.14	.094	[-.29, .02]									

Supplementary Table 4.

Meta-regressions comparing main categories of correlates as categorical predictors of the effect size within each migrant population for psychological and socio-cultural adaptation. A significant result (i.e., $p(r) < .05$) means that the average effect for a given category differs significantly in size from the reference; a non-significant result indicates a lack of significant difference.

Outcome	Group	Predictor	*N*	*k* studies	*n* effects	*B*	*p(B)*	95% CI	*QE*	*df(QE)*	*p(QE)*	*QM*	*df(QM)*	*p(QM)*	τ^2 lv 3	τ^2 lv 2	I^2 total	Pseudo R^2
**Psychological adaptation**																		
	**In migrants**		328,366	422	1,616				512,733	1,612	<.001	48	3	<.001	.01	.02	97.21%	4.63%
		Stressors (reference)				.22	<.001	[.20, .23]										
		Social resources				.01	.252	[-.01, .03]										
		Exposure				-.09	<.001	[-.12, -.06]										
		Cultural distance				-.17	.005	[-.29, -.05]										
	**In expats**		9,580	53	170				1,495	166	<.001	20	3	<.001	.01	.03	88.54%	5.61%
		Stressors (reference)				.20	<.001	[.13, .28]										
		Social resources				.03	.571	[-.06, .11]										
		Exposure				-.05	.380	[-.17, .06]										
		Cultural distance				-.24	<.001	[-.37, -.10]										
	**In students**		78,976	264	1,219				16,951	1,215	<.001	64	3	<.001	.01	.02	90.75%	8.00%
		Stressors (reference)				.27	<.001	[.25, .29]										
		Social resources				-.02	.059	[-.04, .00]										
		Exposure				-.12	<.001	[-.15, -.09]										
		Cultural distance				-.09	.002	[-.14, -.03]										
	**In refugees**		66,072	155	512				31,794	509	<.001	11	2	.004	.02	.01	94.11%	4.41%
		Stressors (reference)				.23	<.001	[.21, .26]										
		Social resources				-.04	.017	[-.08, -.01]										
		Exposure				-.09	.006	[-.16, -.03]										

Supplementary Table 4 (cont.)

Outcome	Group	Predictor	*N*	*k* studies	*n* effects	*B*	*p(B)*	95% CI	*QE*	*df(QE)*	*p(QE)*	*QM*	*df(QM)*	*p(QM)*	τ^2 lv 3	τ^2 lv 2	I^2 total	Pseudo R^2
**Socio-cultural adaptation**																		
	**In migrants**		14,160	54	162				13,844	158	<.001	4	3	.223	.02	.03	95.67%	0.33%
		Stressors (reference)				.27	<.001	[.21, .33]										
		Cultural distance				-.13	.036	[-.25, -.01]										
		Exposure				-.02	.792	[-.21, .16]										
		Social resources				-.02	.561	[-.09, .05]										
	**In expats**		27,541	151	736				11,796	732	<.001	30	3	<.001	.01	.02	88.89%	5.27%
		Stressors (reference)				.24	<.001	[.20, .27]										
		Social resources				.01	.428	[-.02, .05]										
		Exposure				-.04	.113	[-.10, .01]										
		Cultural distance				-.09	<.001	[-.13, -.05]										
	**In students**		36,706	138	535				46,299	531	<.001	57	3	<.001	.02	.02	95.46%	8.96%
		Stressors (reference)				.29	<.001	[.26, .33]										
		Social resources				-.01	.693	[-.05, .03]										
		Exposure				-.16	<.001	[-.20, -.12]										
		Cultural distance				-.07	.084	[-.15, .01]										
	**In refugees**		2,385	10	26				238	22	<.001	1	3	.839	.00	.03	89.00%	-9.94%
		Stressors (reference)				.23	<.001	[.12, .34]										
		Social resources				-.06	.438	[-.23, .10]										
		Exposure				-.02	.806	[-.20, .16]										
		Cultural distance				-.12	.536	[-.49, .26]										

Supplementary Table 5.
Subset analyses of migrant populations (alternative grouping based on wealth), per broad category of correlates of adaptation and per outcome (socio-cultural and psychological adaptation). Effects reported in the *r* column refer to the average effect size within each sub-category.

Outcome & Correlate	Predictor	*N*	*k* studies	*n* effects	*B*	*p(B)*	95% CI	90% PI	*Q*	*p(Q)*	τ^2 lv 3	τ^2 lv 2	I^2 lv 3	I^2 lv 2	I^2 total
**Socio-cultural adaptation**															
**Stressors**															
	Migrants + refugees	11046	45	82	-.26	<.001	[-.31, -.21]	[-.60, .08]	6158	<.001	.01	.03	25.75%	69.39%	95.14%
	Students + expats	39951	160	367	-.26	<.001	[-.29, -.23]	[-.59, .07]	12867	<.001	.02	.02	43.75%	50.03%	93.79%
**Social resources**															
	Migrants + refugees	7375	34	80	.23	<.001	[.17, .30]	[-.11, .58]	8891	<.001	.03	.01	62.64%	33.08%	95.73%
	Students + expats	36883	143	513	.28	<.001	[.25, .30]	[-.02, .58]	82322	<.001	.02	.01	59.37%	36.48%	95.85%
**Exposure**															
	Migrants + refugees	2074	8	12	.21	<.001	[.13, .30]	[-.02, .44]	75	<.001	.00	.02	0.00%	84.16%	84.16%
	Students + expats	15059	69	222	.14	<.001	[.11, .18]	[-.16, .45]	1818	<.001	.01	.02	36.41%	50.97%	87.38%
**Cultural distance**															
	Migrants + refugees	3735	12	14	-.13	.050	[-.26, .00]	[-.51, .25]	244	<.001	.03	.02	49.60%	43.70%	93.30%
	Students + expats	16855	83	169	-.17	<.001	[-.20, -.13]	[-.45, .12]	1370	<.001	.01	.02	37.45%	49.51%	86.96%
**Psychological adaptation**															
**Stressors**															
	Migrants + refugees	384438	476	1406	-.22	<.001	[-.24, -.21]	[-.50, .06]	561065	<.001	.01	.02	43.86%	53.70%	97.56%
	Students + expats	74396	231	642	-.26	<.001	[-.28, -.24]	[-.54, .02]	11978	<.001	.02	.01	47.76%	44.49%	92.25%
**Social resources**															
	Migrants + refugees	173831	221	537	.23	<.001	[.21, .26]	[-.05, .52]	12681	<.001	.02	.01	65.68%	29.51%	95.20%
	Students + expats	47801	175	480	.25	<.001	[.22, .27]	[-.04, .54]	5549	<.001	.01	.02	43.34%	47.14%	90.48%
**Exposure**															
	Migrants + refugees	127633	71	174	.09	<.001	[.06, .11]	[-.09, .26]	1570	<.001	.01	.00	60.69%	32.95%	93.65%
	Students + expats	18833	66	219	.12	<.001	[.09, .15]	[-.09, .33]	897	<.001	.01	.01	31.03%	48.41%	79.44%
**Cultural distance**															
	Migrants + refugees	7593	5	11	-.02	.814	[-.17, .13]	[-.32, .29]	269	<.001	.03	.00	90.10%	8.32%	98.41%
	Students + expats	8408	34	48	-.09	.004	[-.15, -.03]	[-.38, .20]	258	<.001	.03	.00	88.90%	0.00%	88.90%

Supplementary Table 6.
Meta-regressions with migrant population (alternative grouping based on wealth) as categorical predictor of the effect size, per broad category of correlates of adaptation and per outcome (socio-cultural and psychological adaptation). A significant result (i.e., *p(r)* < .05) means that the average effect for a given category differs significantly in size from the reference; a non-significant result indicates a lack of significant difference.

Outcome	Correlate	Predictor	*N*	*k* studies	*n* effects	*r*	*p(r)*	95% CI	*QE*	*df(QE)*	*p(QE)*	*QM*	*df(QM)*	*p(QM)*	τ^2 lv 3	τ^2 lv 2	Pseudo R^2
**Socio-cultural adaptation**																	
	**Cultural distance**		19038	95	183				1614	181	<.001	0	1	.496	.01	.02	0.00%
		Migrants + refugees (baseline)				-.13	.019	[-.23, -.02]									
		Students + expats				-.04	.496	[-.15, .07]									
	**Exposure**		16925	77	234				1893	232	<.001	1	1	.345	.01	.02	0.31%
		Migrants + refugees (baseline)				.20	<.001	[.09, .32]									
		Students + expats				-.06	.345	[-.19, .06]									
	**Social resources**		42651	177	593				91212	591	<.001	1	1	.222	.02	.01	0.08%
		Migrants + refugees (baseline)				.24	<.001	[.18, .30]									
		Students + expats				.04	.222	[-.03, .11]									
	**Stressors**		49949	205	449				19025	447	<.001	0	1	.829	.02	.02	0.00%
		Migrants + refugees (baseline)				-.27	<.001	[-.32, -.21]									
		Students + expats				.01	.829	[-.06, .07]									
**Psychological adaptation**																	
	**Cultural distance**		15362	39	59				527	57	<.001	1	1	.385	.03	.00	0.00%
		Migrants + refugees (baseline)				-.02	.825	[-.17, .14]									
		Students + expats				-.07	.385	[-.24, .09]									
	**Exposure**		143831	137	393				2467	391	<.001	3	1	.067	.01	.01	0.84%
		Migrants + refugees (baseline)				.09	<.001	[.06, .12]									
		Students + expats				.04	.067	[.00, .07]									
	**Social resources**		215985	396	1017				18229	1015	<.001	1	1	.357	.02	.01	0.00%
		Migrants + refugees (baseline)				.23	<.001	[.21, .26]									
		Students + expats				.02	.357	[-.02, .05]									
	**Stressors**		428397	707	2048				573043	2046	<.001	9	1	.003	.01	.02	1.16%
		Migrants + refugees (baseline)				-.22	<.001	[-.24, -.21]									
		Students + expats				-.04	.003	[-.06, -.01]									

Supplementary Table 7.

Meta-regressions with sample gender composition and mean age as predictors of the effect size. A significant result (i.e., *p(r)* < .05) means that the average effect for a given category is associated with sample mean age and gender composition; a non-significant result indicates a lack of association.

Outcome	Correlate	Predictor	*N*	*k* studies	*n* effects	*r*	*p(r)*	95% CI	*QE*	*df(QE)*	*p(QE)*	*QM*	*df(QM)*	*p(QM)*	τ^2 lv 3	τ^2 lv 2	Pseudo R^2
**Psychological adaptation**																	
	**Stressors**																
		**Gender**	437656	698	2012				484303	2010	<.001	0	1	.486	.01	.02	0.00%
		Intercept				-.23	<.001	[-.25, -.20]									
		% male				.00	.486	[.00, .00]									
		**Age**	437656	654	1903				594102	1901	<.001	9	1	.003	.01	.02	0.42%
		Intercept				-.28	<.001	[-.32, -.25]									
		Age				.00	.003	[.00, .00]									
		**Gender + age**	437656	645	1887				432139	1884	<.001	9	2	.009	.01	.02	0.60%
		Intercept				-.28	<.001	[-.33, -.24]									
		% male				.00	.968	[.00, .00]									
		Age				.00	.002	[.00, .00]									
	**Social resources**																
		**Gender**	218040	395	1012				15175	1010	<.001	0	1	.682	.02	.01	0.47%
		Intercept				.23	<.001	[.20, .27]									
		% male				.00	.682	[.00, .00]									
		**Age**	218040	362	942				17770	940	<.001	1	1	.451	.02	.01	0.00%
		Intercept				.26	<.001	[.21, .30]									
		Age				.00	.451	[.00, .00]									
		**Gender + age**	218040	358	935				13411	932	<.001	1	2	.640	.02	.01	0.00%
		Intercept				.25	<.001	[.19, .31]									
		% male				.00	.673	[.00, .00]									
		Age				.00	.434	[.00, .00]									

Supplementary Table 7 (cont.)

Outcome	Correlate	Predictor	*N*	*k* studies	*n* effects	*r*	*p(r)*	95% CI	*QE*	*df(QE)*	*p(QE)*	*QM*	*df(QM)*	*p(QM)*	τ^2 lv 3	τ^2 lv 2	Pseudo R^2
**Psychological adaptation**																	
	**Exposure**																
		**Gender**	145119	137	403				2421	401	<.001	3	1	.062	.01	.01	5.93%
		Intercept				.14	<.001	[.09, .18]									
		% male				.00	.062	[.00, .00]									
		**Age**	145119	127	381				2436	379	<.001	0	1	.873	.01	.01	0.00%
		Intercept				.11	<.001	[.06, .16]									
		Age				.00	.873	[.00, .00]									
		**Gender + age**	145119	124	376				2266	373	<.001	3	2	.198	.01	.01	5.44%
		Intercept				.14	<.001	[.08, .21]									
		% male				.00	.072	[.00, .00]									
		Age				.00	.911	[.00, .00]									
	**Cultural distance**																
		**Gender**	15362	39	59				500	57	<.001	0	1	.964	.03	.00	0.00%
		Intercept				-.08	.330	[-.23, .08]									
		% male				.00	.964	[.00, .00]									
		**Age**	15362	36	55				267	53	<.001	13	1	<.001	.01	.00	56.31%
		Intercept				-.40	<.001	[-.56, -.24]									
		Age				.01	<.001	[.00, .01]									
		**Gender + age**	15362	36	55				232	52	<.001	16	2	<.001	.01	.00	58.98%
		Intercept				-.33	<.001	[-.51, -.15]									
		% male				.00	.140	[.00, .00]									
		Age				.01	<.001	[.01, .02]									

Supplementary Table 7 (cont.)

Outcome	Correlate	Predictor	*N*	*k* studies	*n* effects	*r*	*p(r)*	95% CI	*QE*	*df(QE)*	*p(QE)*	*QM*	*df(QM)*	*p(QM)*	τ^2 lv 3	τ^2 lv 2	Pseudo R^2
**Socio-cultural adaptation**																	
	**Stressors**																
		**Gender**	49574	197	418				19165	416	<.001	0	1	.609	.02	.02	2.51%
		Intercept				-.28	<.001	[-.35, -.21]									
		% male				.00	.609	[.00, .00]									
		**Age**	49574	183	408				18268	406	<.001	5	1	.023	.02	.02	4.06%
		Intercept				-.38	<.001	[-.47, -.28]									
		Age				.00	.023	[.00, .01]									
		**Gender + age**	49574	176	381				17774	378	<.001	5	2	.073	.02	.02	0.52%
		Intercept				-.38	<.001	[-.49, -.27]									
		% male				.00	.995	[.00, .00]									
		Age				.00	.025	[.00, .01]									
	**Social resources**																
		**Gender**	42928	168	543				83642	541	<.001	0	1	.994	.02	.01	0.26%
		Intercept				.27	<.001	[.20, .35]									
		% male				.00	.994	[.00, .00]									
		**Age**	42928	143	496				52559	494	<.001	4	1	.045	.02	.01	0.00%
		Intercept				.38	<.001	[.27, .48]									
		Age				.00	.045	[-.01, .00]									
		**Gender + age**	42928	136	458				43494	455	<.001	4	2	.113	.02	.01	0.52%
		Intercept				.37	<.001	[.24, .49]									
		% male				.00	.609	[.00, .00]									
		Age				.00	.038	[-.01, .00]									

Supplementary Table 7 (cont.)

Outcome	Correlate	Predictor	*N*	*k* studies	*n* effects	*r*	*p(r)*	95% CI	*QE*	*df(QE)*	*p(QE)*	*QM*	*df(QM)*	*p(QM)*	τ^2 lv 3	τ^2 lv 2	Pseudo R^2
**Socio-cultural adaptation**																	
	**Exposure**																
		**Gender**	17082	73	228				1863	226	<.001	0	1	.557	.01	.02	0.00%
		Intercept				.17	<.001	[.08, .27]									
		% male				.00	.557	[.00, .00]									
		**Age**	17082	70	226				1793	224	<.001	0	1	.850	.01	.02	0.00%
		Intercept				.13	.021	[.02, .24]									
		Age				.00	.850	[.00, .00]									
		**Gender + age**	17082	64	208				1686	205	<.001	1	2	.567	.01	.02	0.00%
		Intercept				.16	.015	[.03, .30]									
		% male				.00	.303	[.00, .00]									
		Age				.00	.603	[.00, .00]									
	**Cultural distance**																
		**Gender**	19348	94	174				1573	172	<.001	2	1	.128	.01	.02	0.00%
		Intercept				-.23	<.001	[-.32, -.14]									
		% male				.00	.128	[.00, .00]									
		**Age**	19348	91	179				1469	177	<.001	2	1	.117	.01	.02	4.26%
		Intercept				-.28	<.001	[-.43, -.13]									
		Age				.00	.117	[.00, .01]									
		**Gender + age**	19348	87	167				1443	164	<.001	3	2	.210	.01	.02	0.00%
		Intercept				-.30	<.001	[-.46, -.14]									
		% male				.00	.403	[.00, .00]									
		Age				.00	.262	[.00, .01]									

Supplementary Table 8.
Subset analyses of sub-categories of adaptation correlates within each main category and their associations with psychological and socio-cultural adaptation. Effects reported in the *r* column refer to the average effect size within each sub-category.

	**Outcome, Correlate, & Predictor**	***N***	***k* studies**	***n* effects**	***r***	***p(r)***	**95% CI**	**90% PI**	***Q***	***p(Q)***	**τ^2 lv 3**	**τ^2 lv 2**	**I^2 lv 3**	**I^2 lv 2**	**I^2 total**
	**Psychological adaptation**														
**Stressors**	Acculturative stressors	54,872	186	334	-.35	<.001	[-.37, -.32]	[-.63, -.06]	54,185	<.001	.02	.01	53.66%	42.14%	95.81%
	Occupational stressors	16,636	40	68	-.28	<.001	[-.34, -.22]	[-.59, .03]	1,183	<.001	.03	.00	81.17%	12.18%	93.35%
	COVID-stressors	10,016	17	61	-.27	<.001	[-.35, -.19]	[-.57, .04]	1,882	<.001	.02	.01	72.97%	23.51%	96.48%
	Perceived discrimination	229,791	269	545	-.26	<.001	[-.28, -.24]	[-.52, .00]	12,041	<.001	.01	.01	52.43%	42.15%	94.59%
	General stressors	42,383	100	198	-.26	<.001	[-.29, -.23]	[-.53, .00]	2,903	<.001	.02	.01	66.77%	24.93%	91.69%
	Loss of social status	7,843	24	41	-.26	<.001	[-.32, -.19]	[-.50, -.01]	465	<.001	.02	.00	70.11%	17.94%	88.06%
	Family stressors	25,158	36	80	-.24	<.001	[-.31, -.17]	[-.60, .12]	1,851	<.001	.04	.00	92.07%	2.91%	94.97%
	Language barrier	193,372	234	362	-.15	<.001	[-.17, -.13]	[-.37, .07]	3,233	<.001	.01	.01	52.18%	38.59%	90.78%
	Visa status	24,830	47	83	-.14	<.001	[-.19, -.10]	[-.40, .11]	1,232	<.001	.02	.00	80.66%	11.21%	91.87%
	Low socio-economic status	228,602	196	301	-.14	<.001	[-.16, -.12]	[-.39, .10]	44,643	<.001	.01	.01	50.32%	45.66%	95.98%
**Social resources**	**Type**														
	Social connectedness	8,083	29	94	.33	<.001	[.27, .39]	[.05, .61]	1,018	<.001	.02	.01	54.01%	37.13%	91.15%
	Lack of loneliness	33,927	51	73	.31	<.001	[.26, .36]	[.04, .58]	1,563	<.001	.02	.00	84.60%	9.50%	94.10%
	Organizational support	5,672	30	45	.25	<.001	[.18, .32]	[-.06, .56]	459	<.001	.02	.01	53.85%	34.34%	88.19%
	Quality of social interactions	13,161	41	107	.24	<.001	[.19, .29]	[-.07, .55]	1,391	<.001	.02	.01	55.29%	37.42%	92.71%
	Social support	180,021	298	682	.23	<.001	[.21, .24]	[-.05, .50]	13,483	<.001	.02	.01	65.77%	28.25%	94.02%
	Social permeability	562	3	4	.15	<.001	[.08, .22]	[.09, .21]	2	.659	.00	.00	0.00%	0.00%	0.00%
	Friendship strength	1,552	11	24	.08	.205	[-.04, .19]	[-.25, .40]	104	<.001	.03	.01	67.61%	15.76%	83.37%
	**Source**														
	Unspecific	121,870	291	579	.27	<.001	[.25, .29]	[-.03, .56]	9,686	<.001	.02	.01	61.85%	31.42%	93.27%
	Organizations	5,547	30	45	.27	<.001	[.19, .34]	[-.06, .60]	501	<.001	.03	.01	61.75%	27.87%	89.63%
	Peers	3,678	16	19	.26	<.001	[.16, .36]	[-.07, .58]	184	<.001	.04	.00	90.24%	0.00%	90.24%
	Supervisors	2,455	12	16	.26	<.001	[.18, .34]	[.04, .48]	81	<.001	.01	.00	56.57%	22.61%	79.18%
	Local nationals	21,641	71	134	.21	<.001	[.18, .24]	[.00, .42]	952	<.001	.01	.01	47.63%	37.86%	85.49%
	Family	16,729	53	89	.17	<.001	[.13, .21]	[-.06, .41]	662	<.001	.01	.01	44.21%	42.10%	86.31%
	Spouses	73,173	13	19	.17	.002	[.06, .27]	[-.18, .52]	324	<.001	.01	.03	27.51%	71.93%	99.44%
	Other internationals	4,321	17	27	.14	<.001	[.11, .17]	[.05, .23]	46	.009	.00	.00	3.82%	36.56%	40.38%
	Co-nationals	17,102	54	101	.11	<.001	[.07, .14]	[-.10, .31]	577	<.001	.01	.01	50.36%	31.99%	82.35%

Supplementary Table 8 (cont.)

Outcome, Correlate, & Predictor		*N*	*k* studies	*n* effects	*r*	*p(r)*	95% CI	90% PI	*Q*	*p(Q)*	τ^2 lv 3	τ^2 lv 2	I^2 lv 3	I^2 lv 2	I^2 total
**Psychological adaptation**															
**Exposure**	Local nationals	22,227	61	114	.15	<.001	[.12, .17]	[-.02, .31]	635	<.001	.01	.00	54.53%	26.55%	81.08%
	Unspecific	122,555	74	159	.11	<.001	[.08, .14]	[-.09, .31]	1,163	<.001	.01	.01	61.84%	32.84%	94.68%
	Other internationals	2,641	12	32	.08	.002	[.03, .13]	[-.08, .24]	102	<.001	.00	.01	25.10%	43.49%	68.59%
	Co-nationals	15,022	50	109	.05	.002	[.02, .09]	[-.13, .24]	397	<.001	.01	.00	74.67%	4.60%	79.26%
**Cultural distance**	Self-rated measures	9,791	32	50	-.09	.009	[-.16, -.02]	[-.39, .21]	278	<.001	.03	.00	93.59%	0.00%	93.59%
	Objective measures	6,210	7	9	-.02	.595	[-.09, .05]	[-.19, .15]	84	<.001	.00	.01	0.00%	93.07%	93.07%
**Socio-cultural adaptation**															
**Exposure**	Local nationals	14,049	58	113	.22	<.001	[.17, .26]	[-.11, .54]	1,024	<.001	.02	.02	49.17%	40.58%	89.75%
	Other internationals	1,748	10	17	.10	.045	[.00, .20]	[-.17, .37]	99	<.001	.01	.01	40.56%	40.45%	81.01%
	Unspecific	4,534	26	60	.09	<.001	[.04, .15]	[-.13, .32]	228	<.001	.01	.01	51.03%	26.12%	77.15%
	Co-nationals	5,863	32	56	.05	.074	[.00, .10]	[-.19, .28]	284	<.001	.01	.01	35.32%	43.74%	79.06%
**Cultural distance**	Self-rated measures	16,992	78	143	-.20	<.001	[-.23, -.16]	[-.47, .08]	1,153	<.001	.02	.01	47.17%	40.32%	87.49%
	Objective measures	3,954	21	43	-.08	.045	[-.15, .00]	[-.40, .25]	391	<.001	.01	.03	28.58%	59.34%	87.92%

Supplementary Table 8 (cont.)

	**Outcome, Correlate, & Predictor**	***N***	***k* studies**	***n* effects**	***r***	***p(r)***	**95% CI**	**90% PI**	***Q***	***p(Q)***	**τ^2 lv 3**	**τ^2 lv 2**	**I^2 lv 3**	**I^2 lv 2**	**I^2 total**
	**Socio-cultural adaptation**														
**Stressors**	Perceived discrimination	18,899	57	82	-.38	<.001	[-.43, -.33]	[-.70, -.06]	2,107	<.001	.02	.01	63.43%	31.46%	94.89%
	Acculturative stressors	6,688	31	35	-.30	<.001	[-.40, -.20]	[-.78, .19]	4,283	<.001	.00	.08	0.00%	98.44%	98.44%
	General stressors	3,023	12	20	-.30	<.001	[-.40, -.20]	[-.59, -.01]	206	<.001	.03	.00	81.19%	7.76%	88.95%
	Language barrier	34,801	132	236	-.24	<.001	[-.26, -.21]	[-.49, .02]	2,217	<.001	.01	.01	44.09%	43.43%	87.53%
	Occupational stressors	4,404	20	39	-.17	.010	[-.30, -.04]	[-.69, .35]	3,310	<.001	.05	.04	54.75%	42.66%	97.41%
	Low socio-economic status	3,690	20	22	-.15	.002	[-.24, -.05]	[-.49, .19]	235	<.001	.04	.00	89.40%	0.00%	89.40%
	Loss of social status	1,751	11	21	-.12	.013	[-.22, -.03]	[-.41, .16]	127	<.001	.01	.02	27.38%	56.38%	83.77%
**Social resources**	**Type**														
	Social connectedness	1,846	8	12	.38	<.001	[.27, .48]	[.11, .65]	96	<.001	.01	.02	31.74%	57.48%	89.22%
	Lack of loneliness	1,635	6	10	.30	.001	[.12, .48]	[-.20, .80]	598	<.001	.00	.08	0.00%	97.75%	97.75%
	Organizational support	13,085	56	145	.28	<.001	[.24, .33]	[-.03, .60]	17,522	<.001	.02	.01	60.41%	35.43%	95.83%
	Quality of social interactions	3,736	18	39	.27	<.001	[.20, .34]	[.01, .53]	242	<.001	.01	.01	34.06%	50.22%	84.29%
	Social support	30,318	115	356	.25	<.001	[.22, .28]	[-.06, .56]	59,316	<.001	.03	.01	72.92%	23.29%	96.21%
	Friendship strength	1,096	8	28	.17	<.001	[.10, .24]	[-.04, .38]	90	<.001	.00	.01	10.36%	57.38%	67.75%
	Social permeability	576	3	8	.14	.160	[-.06, .34]	[-.18, .46]	30	<.001	.03	.00	81.89%	0.00%	81.89%
	**Source**														
	Supervisors	5,276	22	49	.32	<.001	[.25, .39]	[.04, .60]	1,140	<.001	.02	.01	71.47%	20.11%	91.57%
	Unspecific	20,394	76	147	.29	<.001	[.25, .33]	[-.02, .61]	23,714	<.001	.02	.01	58.62%	38.29%	96.91%
	Organizations	12,860	57	147	.29	<.001	[.24, .34]	[-.04, .62]	17,398	<.001	.02	.01	61.24%	34.91%	96.14%
	Local nationals	7,819	37	88	.29	<.001	[.23, .35]	[-.01, .59]	710	<.001	.03	.01	72.17%	14.97%	87.14%
	Spouses	1,746	8	11	.28	<.001	[.16, .39]	[.00, .55]	92	<.001	.02	.00	86.02%	0.00%	86.02%
	Peers	5,616	21	70	.23	<.001	[.17, .29]	[.02, .44]	366	<.001	.01	.00	75.49%	5.58%	81.07%
	Family	5,671	18	35	.15	.004	[.05, .25]	[-.21, .51]	1,146	<.001	.04	.00	87.34%	8.87%	96.21%
	Co-nationals	3,943	25	41	.11	<.001	[.06, .16]	[-.07, .28]	115	<.001	.01	.00	63.77%	0.00%	63.77%
	Other internationals	1,115	7	10	.08	.252	[-.06, .22]	[-.23, .39]	36	<.001	.03	.00	71.87%	12.63%	84.50%

Supplementary Table 9.
Meta-regressions of sub-categories of correlates within each main category as categorical predictors of psychological and socio-cultural adaptation. A significant result (i.e., *p(r)* < .05) means that the average effect for a given category differs significantly in size from the reference category; a non-significant result indicates a lack of significant difference. Note that for social resources, two models were fitted, one for the breakdown by type of resources and one by social group that served as the source of resources.

Outcome & Correlate	Predictor	*N*	*k* studies	*n* effects	*B*	*p(B)*	95% CI	*QE*	*df(QE)*	*p(QE)*	*QM*	*df(QM)*	*p(QM)*	τ^2 lv 3	τ^2 lv 2	Pseudo R^2
**Psychological adaptation**																
**Stressors**		437,656	719	2,073				123,617	2,063	<.001	307	9	<.001	.01	.01	16.38%
	Acculturative stressors (reference)				-.34	<.001	[-.36, -.32]									
	Occupational stressors				.05	.019	[.01, .09]									
	COVID19-stressors				.05	.082	[-.01, .11]									
	Discrimination				.08	<.001	[.05, .10]									
	General stressors				.08	<.001	[.05, .11]									
	Loss of social status				.10	<.001	[.05, .16]									
	Family stressors				.10	<.001	[.06, .15]									
	Language barrier				.18	<.001	[.16, .20]									
	Visa status				.16	<.001	[.12, .20]									
	Low socio-economic status				.18	<.001	[.15, .20]									
**Social resources**																
	**Types**	217,893	399	1,029				18,020	1,022	<.001	49	6	<.001	.02	.01	3.23%
	Social connectedness (reference)				.32	<.001	[.27, .37]									
	Lack of loneliness				-.01	.847	[-.07, .06]									
	Organizational support				-.09	.017	[-.16, -.02]									
	Quality of social interaction				-.08	.010	[-.15, -.02]									
	Social support				-.09	<.001	[-.15, -.04]									
	Social permeability				-.11	.233	[-.30, .07]									
	Friendship strength				-.25	<.001	[-.34, -.17]									
	**Sources**	217,893	399	1,029				13,013	1,020	<.001	67	8	<.001	.02	.01	8.16%
	Supervisors (reference)				.22	<.001	[.14, .30]									
	Unspecific				.05	.280	[-.04, .13]									
	Organizations				.02	.692	[-.08, .11]									
	Peers				.03	.592	[-.07, .13]									
	Local nationals				.00	.920	[-.08, .09]									
	Family				.00	.996	[-.09, .09]									
	Spouses				-.02	.781	[-.13, .10]									
	Other internationals				-.09	.065	[-.19, .01]									
	Co-nationals				-.09	.041	[-.18, .00]									

Supplementary Table 9 (cont.)

Outcome & Correlate	Predictor	*N*	*k* studies	*n* effects	*B*	*p(B)*	95% CI	*QE*	*df(QE)*	*p(QE)*	*QM*	*df(QM)*	*p(QM)*	τ² lv 3	τ² lv 2	Pseudo R^2
**Psychological adaptation**																
**Exposure**		145,119	144	414				2,297	410	<.001	39	3	<.001	.01	.01	7.03%
	Local nationals (reference)				.14	<.001	[.12, .17]									
	Unspecific				-.03	.103	[-.06, .01]									
	Other internationals				-.05	.030	[-.10, -.01]									
	Co-nationals				-.10	<.001	[-.13, -.07]									
**Cultural distance**		15,362	39	59				362	57	<.001	1	1	.476	.03	.00	-0.99%
	Objective measures (reference)				-.04	.605	[-.17, .10]									
	Self-rated measures				-.05	.476	[-.21, .10]									
**Socio-cultural adaptation**																
**Exposure**		17,082	79	246				1,635	242	<.001	45	3	<.001	.02	.01	9.04%
	Local nationals (reference)				.21	<.001	[.17, .25]									
	Other internationals				-.07	.126	[-.16, .02]									
	Unspecific				-.10	<.001	[-.16, -.04]									
	Co-nationals				-.18	<.001	[-.23, -.12]									
**Cultural distance**		19,348	98	186				1,544	184	<.001	11	1	<.001	.01	.02	5.71%
	Objective measures (reference)				-.06	.065	[-.13, .00]									
	Self-rated measures				-.13	<.001	[-.20, -.05]									

Supplementary Table 9 (cont.)

Outcome & Correlate	Predictor	N	k studies	n effects	B	$p(B)$	95% CI	QE	$df(QE)$	$p(QE)$	QM	$df(QM)$	$p(QM)$	τ^2 lv 3	τ^2 lv 2	Pseudo R^2
**Socio-cultural adaptation**																
	**Stressors**	49,574	210	457				12,511	450	<.001	49	6	<.001	.02	.02	10.16%
	Perceived discrimination (reference)				-.38	<.001	[-.43, -.34]									
	Acculturative stressors				.09	.017	[.02, .17]									
	General stressors				.14	.004	[.05, .24]									
	Language barrier				.14	<.001	[.09, .19]									
	Occupational stressors				.18	<.001	[.10, .26]									
	Low socio-economic status				.23	<.001	[.14, .33]									
	Loss of social status				.22	<.001	[.12, .32]									
	**Social resources**															
	**Types**	42,818	179	598				77,895	591	<.001	18	6	.008	.02	.01	1.02%
	Social connectedness (reference)				.38	<.001	[.28, .49]									
	Lack of loneliness				-.02	.797	[-.20, .16]									
	Organizational support				-.09	.123	[-.20, .02]									
	Quality of social interaction				-.10	.118	[-.23, .03]									
	Social support				-.14	.013	[-.24, -.03]									
	Friendship strength				-.17	.006	[-.28, -.05]									
	Social permeability				-.21	.018	[-.39, -.04]									
	**Sources**	42,818	179	598				44,717	589	<.001	65	8	<.001	.02	.01	7.42%
	Supervisors (reference)				.28	<.001	[.22, .33]									
	Unspecific				.01	.670	[-.05, .07]									
	Organizations				.02	.423	[-.03, .08]									
	Local nationals				.01	.774	[-.06, .07]									
	Spouses				-.06	.269	[-.16, .04]									
	Peers				.02	.612	[-.05, .09]									
	Family				-.14	<.001	[-.21, -.07]									
	Co-nationals				-.11	.003	[-.19, -.04]									
	Other internationals				-.18	<.001	[-.29, -.08]									

Supplementary Table 10.

Subset analyses of sub-categories of social support (socio-emotional, instrumental) and their associations with psychological and socio-cultural adaptation. Effects reported in the *r* column refer to the average effect size within each sub-category.

Outcome	Predictor	*N*	*k* studies	*n* effects	*B*	*p(B)*	95% CI	90% PI	*Q*	*p(Q)*	τ^2 lv 3	τ^2 lv 2	I^2 lv 3	I^2 lv 2	I^2 total
**Psychological adaptation**															
	Socio-emotional Support	7679	28	59	.19	<.001	[.11, .26]	[-.14, .52]	556	<.001	.03	.01	59.87%	32.38%	92.25%
	Instrumental Support	6558	29	52	.23	<.001	[.18, .28]	[.02, .44]	294	<.001	.01	.01	42.64%	36.44%	79.08%
**Socio-cultural adaptation**															
	Socio-emotional Support	2133	10	25	.32	<.001	[.28, .37]	[.23, .42]	46	.005	.00	.00	34.65%	12.62%	47.28%
	Instrumental Support	3882	17	61	.20	<.001	[.14, .27]	[-.02, .43]	418	<.001	.01	.01	46.46%	34.20%	80.66%

Supplementary Table 11.
Meta-regressions comparing two subcategories of social support (socio-emotional, instrumental) and their associations with psychological and socio-cultural adaptation. A significant result (i.e., *p(r)* < .05) means that the average effect for a given category differs significantly in size from the reference; a non-significant result indicates a lack of significant difference.

**Outcome**	**Predictor**	***N***	***k* studies**	***n* effects**	***r***	***p(r)***	**95% CI**	***QE***	***df(QE)***	***p(QE)***	***QM***	***df(QM)***	***p(QM)***	**τ^2 lv 3**	**τ^2 lv 2**	**Pseudo R^2**
**Socio-cultural adaptation**		4456	21	86				463	84	<.001	4	1	.041	.01	.01	11.92%
	Instrumental (baseline)				.22	<.001	[.16, .27]									
	Socio-emotional				.06	.041	[.00, .13]									
**Psychological adaptation**		9678	39	111				849	109	<.001	0	1	.654	.02	.01	0.98%
	Instrumental (baseline)				.21	<.001	[.15, .27]									
	Socio-emotional				-.01	.654	[-.06, .04]									

Supplementary Table 12.

Examples of eligible measures for each broader category and subcategory of adaptation outcomes and correlates. Please note that the list is not exhaustive. *Many acculturative stressor scales include subscales such as perceived discrimination or language barrier, which in our meta-analysis are analyzed separately. Thus, the acculturative stressor subcategory was only used in cases when a study only reported a composite score involving all subscales of an acculturative stressor scale and did not provide separate scores for the subscales of our interest. In cases when separate scores were provided (e.g., perceived discrimination), we instead coded those separate scores. Note that categorizations resulting from this coding strategy were fully independent (i.e., an effect could be coded as pertaining to acculturative stressors or perceived discrimination, but never both), ensuring that all subcategories could be used in the same meta-regression.

Broad Category	Subcategory	Examples of Included Measures	Sample Items
**Psychological Adaptation**	NA	Satisfaction with Life Scale (Diener, Emmons, Larsen, & Griffin, 1985);<br>Subjective Happiness Scale (Lyubomirsky & Lepper, 1999);<br>Brief Symptom Inventory (Derogatis & Spencer, 1982) (Reversed);<br>Hopkins Symptom Checklist (Mollica et al., 1987) (Reversed)	The conditions of my life are excellent (1 = Strongly disagree; 7 = Strongly agree).<br><br>I am satisfied with my life (1 = Strongly disagree; 7 = Strongly agree).<br><br>In general, I consider myself… (1 = Not a very happy person; 7 = Very happy person).<br><br>During the past 7 days, how much were you distressed by (0 = Not at all; 4 = Extremely): Feeling easily annoyed or irritated; Feeling no interest in things; Feeling inferior to others.
**Socio-cultural Adaptation**	NA	Sociocultural Adaptation Scale (Ward and Kennedy, 1999);<br>Brief Sociocultural Adaptation Scale (Demes & Geeraert, 2014);<br>Social Situation Questionnaire (Furnham & Bochner, 1982)	Thinking about life in [country], please rate your competence at each the following behaviours (1 = Not at all competent; 5 = Extremely competent): Adapting to the local accommodation; Dealing with the climate; Adapting to local etiquette (manners, customs); etc.<br><br>Think about living in [host country]. How easy or difficult is it for you to adapt to... (1 = very difficult; 7 = very easy): Climate (temperature, rainfall, humidity); Social environment (size of the community, pace of life, noise); Food and eating (what food is eaten, how food is eaten, time of meals); Social norms (how to behave in public, style of clothes, what people think is funny); etc.
**Stressors**	Acculturative*	Acculturation Stress Index (Noh, Wu, & Avison, 1994);<br>Riverside Acculturation Stress Inventory (Benet-Martınez & Haritatos, 2005);<br>Acculturative Hassles Scale (Pan et al., 2010)	Because of my [race/ethnic] background, I have to work harder than most [host country individuals].<br><br>I feel that my particular practices [home/host cultures] have caused conflict in my relationships.<br><br>It bothers me that I have an accent.
	Low SES	Income (reversed);<br>Two-Factor Index of Social Position (Hollingshead, 1957);	Reported parental occupation and education were coded using a seven-point scale ranging from 1 (higher executives, proprietors, major professionals; professional degree) to 7 (unskilled employees; less than seven years of school).
	COVID-19	COVID-19 Perceived Risk Scale (Yıldırım and Güler, 2020);<br>COVID-19 Traumatic Stress Scale (Kira, Shuwiekh, Rice, et al., 2020)	Worry about oneself contracting COVID-19 (1 = negligible; 5 = very large).<br><br>Worry about COVID-19 occurring in the region (1 = negligible; 5 = very large).<br><br>Perceived likelihood of acquiring COVID-19 (1 = negligible; 5 = very large).<br><br>Over the past two weeks, I have felt nervous and fearful about the future because of the coronavirus (0 = never; 4 = many times).<br><br>Over the past two weeks, my life routines have been affected by the coronavirus situation (0 = never; 4 = many times).

Supplementary Table 12 (cont.)

Broad Category	Subcategory	Examples of Included Measures	Sample Items

**Stressors**	Discrimination	Perceived Discrimination Scale (Phinney et al., 1998);<br>Perceived Racism Scale (Hocoy, 1994);<br>Perceived Ethnic Discrimination (Brondolo et al., 2005)	How often do the following people treat you unfairly or negatively because of your ethnic background (1 = almost never; 5 = very often)?<br><br>I feel that I am not wanted in [Host Country] society (1 = almost never; 5 = very often).<br><br>Because of my ethnicity... (1 = never happened; 5 = happened very often): Made you feel like an outsider because of appearance; Hinted you must be lazy; Hinted you must not be clean; Not trusted you; etc.
	General	Index of Life Stress (Yang & Clum, 1995);<br>General Hassles (Lay & Nguyen, 1998);<br>Post-Migration Living Difficulties Questionnaire (Laban et al., 2005)	Requests individuals to rate each statement from never (0) to often (3) according to how often the individual "feels the way described in each statement.": I worry about my financial situation; I worry about my academic performance; It's hard for me to develop opposite-sex relationships here; etc.<br><br>For each item, respondents were asked to rate the "frequency of experience over the past two to three weeks" (0 = Not at all part of my life, 3 = Very much part of my life): Not enough time to meet my obligations; Being let down or disappointed by friends; Lack of money; Dissatisfaction with my physical appearance; Not keeping up with assigned readings; etc.
	Language	Speaking, writing, reading, understanding, and/or listening level in host country language.	I feel confident in:(a) using English in general, (b) writing in English, (c) speaking English, (d) reading and understanding English, and (e) listening to English (0 = very uncomfortable; 5 = very comfortable).
	Occupational	Student-life Stress Inventory (Gadzella, 1991);<br>Job Security (De Witte, 2000);<br>Work Constraints (Bader et al., 2016);<br>Work-life Balance (O'Connell et al., 2004)	As a student (1 = Never; 5 = Most of the time): I have experienced frustrations due to delays in reaching my goals; I feel I was denied opportunities in spite of my qualifications; etc.<br><br>How large, in your opinion, is the probability that you will become unemployed in the near future? (0 = very small or impossible; 5 = very large).
	Status Loss	MacArthur Scale of Subjective Social Status (Adler, 2007) (reversed)<br>International Socio-Economic Index of occupational status	Respondents view a drawing of a ladder with 10 rungs, and are told: "At the top of the ladder are the people who are the best off, those who have the most money, most education, and best jobs. At the bottom are the people who are the worst off, those who have the least money, least education, worst jobs, or no job. Please place an 'X' on the rung that best represents where you think you stand on the ladder."
	Visa Status	Binary measure: Documented vs. Undocumented	NA

Supplementary Table 12 (cont.)

Broad Category	Subcategory	Examples of Included Measures	Sample Items
**Resources**	Connectedness	Social Connectedness Scale (Lee and Robbins, 1995); School Connectedness Measure (McNeely et al., 2002)	Items were placed on a 6-point Likert continuum (1 = strongly agree; 6 = strongly disagree): I feel disconnected from the world around me; I feel so distant from people; I don't feel I participate with anyone or any group; I catch myself losing all sense of connectedness with society; etc.
	Organizational Support	Survey of Perceived Organizational Support (Eisenberger et al., 1986); Psychosocial Safety Climate (Bailey, Dollard & Richards, 2015)	Employees used a 7-point Likert scale (1 = strongly disagree, 7 = strongly agree) to indicate the extent of their agreement with each item: If my home company could hire someone to replace me at a lower salary it would do so; My home company fails to appreciate any extra effort from me; My home company strongly considers my goals and values; My home company would ignore any complaint form; Help is available from my home company when I have a problem; etc.
	Family	Perceived Family Support (Nasurdin & O'Driscoll, 2012)	Responses to the items were made on a 5-point scale (1 = strongly disagree; 5 = strongly agree): My family members do their fair share of household chores; When I have a problem at work, members of my family express concern, etc.
	Friendship Strength	Strength of Support Network (Bruque et al. 2008)	My relationship with this person is very close (1 = totally disagree; 7 = totally agree).
	Loneliness (reversed)	UCLA Loneliness Scale (Russell, Peplau, and Ferguson, 1978)	Participants rate each item on a scale (1 = I often feel this way; 4 = I never feel this way): I have nobody to talk to; I feel as if nobody really understands me; There is no one I can turn to; I am no longer close to anyone; I feel left out; No one really knows me well; It is difficult for me to make friends; etc.
	Quality	The Relational Health Indices (Liang et al., 2007); Positive Intergroup Contact (Barlow et al., 2012)	Next to each statement below, please indicate the number that best applies to your relationship... (1 = Never; 5 = Always): Even when I have difficult things to share, I can be honest and real with my friend; I have a greater sense of self-worth through my relationship with my friend; I can talk to my friend about our disagreements without feeling judged; My friendship causes me to grow in important ways; etc.<br><br>On average, how frequently do you have POSITIVE/GOOD contact with [host country] people (1 = Never to 7 = Extremely Frequently)?
	Social Permeability	Perceived Permeability (Pelly, 1997); Social and Resource Permeability (Terry et al., 2001)	If you wanted to, how easy would it be for you to become involved in social activities with [host country] students (1 = Extremely easy; 7 = Extremely difficult)?<br><br>How much access do [home country] students have to the resources that are available to [host country] students (1 = None at all; 7 = A great deal)?
	Social Support	The Multidimensional Scale of Perceived Social Support (Zimet at el., 1988); Sojourner Social Support (Ong & Ward, 2005); Social Support Scale (Carver, 2006)	Each item was rated on a 5-point Likert scale (1 = strongly disagree; 5 = strongly agree): There is a special person who is around when I am in need; There is a special person with whom I can share my joys and sorrows.<br><br>Respondents were asked to read each item and consider if they knew persons in [host country] with whom they were maintaining some form of regular contact who would perform the helpful behaviors, using a 5-point, Likert-type rating scale (1 = no one would do this; 5 = many would do this): Comfort you whenever you feel homesick; Listen and talk with you whenever you feel lonely or depressed; Provide necessary information to help orient you to your new surroundings; Help you deal with some local institutions' official rules and regulations.
	Support from Supervisors	Leader-Member Exchange Scale (Liden & Maslyn, 1998)	Items were measured on a 7-point Likert scale (1 = completely disagree, 7 = completely agree): My supervisor defends my work actions to a superior, even without complete knowledge of the issue in question; My supervisor would come to my defense if I were "attacked" by others; My supervisor is a lot of fun to work with, etc.

Supplementary Table 12 (cont.)

Broad Category	Subcategory	Examples of Included Measures	Sample Items
Exposure	NA	Contact frequency (Harwood et al., 2005);<br>Network Ethnic Density (Arevalo et al., 2015);<br>Participation in activities (Elfadl et al., 2020)	How often do you interact with co-nationals? (in parties, in activity groups...)? (1-5: not often at all - very often)<br><br>Neighborhood Ethnic Density was measured using the [census] indicator of the proportion of [immigrants] within the population of a census tract. Based on this distribution, a median split was performed to create a dichotomous ethnic density variable.<br><br>How many times have you participated in the activities of the following organizations, associations, societies, or others during the last 12 months? - with seven sub-categories such as sport clubs, culture associations, religious communities, and informal hobby groups, each answered with a 5-point scale ranging from “three times per week or more” to “not taken part in any activity”.
Cultural Distance	Objective*	Kogut & Singh (1988);<br>Dow & Karunaratna (2006)	NA
	Self-rated	Perceived Cultural Distance (Babiker et al., 1980);<br>Cultural Novelty Scale (Black & Stephens, 1989);<br>Cultural Distance Scale (Bektas, 2004)	How much your background and experience in your home country differs from your experience in [host country] on: pace of life, clothing, etc.<br><br>Think about [home country] and [host country]. In your opinion, how different or similar are these two countries in terms of: Social environment (size of the community, pace of life, noise); Living (hygiene, sleeping practices, how safe you feel); Practicalities (getting around, using public transport, shopping); Food and eating (what food is eaten, how food is eaten, time of meals); Family life (how close family members are, how much time family spend together); Social norms (how to behave in public, style of clothes, what people think is funny); Values and beliefs (what people think about religion and politics, what people think is right or wrong), etc.

*Distance calculated by authors of primary studies based on external scores, such as the Hofstede cultural dimensions.

Supplementary Table 13.

Sensitivity analyses based on the criteria of risk of bias (ROB) assessment and publication status. Note that ROB1 refers to whether the criteria for inclusion in the sample were clearly defined. ROB2 refers to whether the participants and the setting were described in detail. ROB3 refers to whether the predictor variables were measured in a valid and reliable way. ROB4 refers to whether confounding bias was accounted for. ROB7 refers to whether the outcome variables were measured in a valid and reliable way. ROB8 refers to whether the statistical information was appropriate, and correlations were reported directly (rather than converted from other statistics). Outliers refer to |r| ≥ .80. More details can be found in Vu et al. (in press).

Outcome	Correlate	ROB criterion	*N*	*k* studies	*n* effects	*r*	*p(r)*	95% CI	90% PI	*Q*	*p(Q)*	τ^2 lv 3	τ^2 lv 2	I^2 lv 3	I^2 lv 2	I^2 total
**Psychological adaptation**																
	**Stressors**	Overall	470,588	719	2,073	-.23	<.001	[-.25, -.22]	[-.52, .05]	654,086	<.001	.01	.02	46.78%	50.35%	97.13%
		ROB1 biased studies removed	459,316	709	2,050	-.23	<.001	[-.25, -.22]	[-.52, .05]	82,597	<.001	.01	.02	45.89%	49.12%	95.01%
		ROB2 biased studies removed	250,351	403	1,208	-.24	<.001	[-.26, -.23]	[-.52, .04]	64,180	<.001	.01	.02	37.70%	57.41%	95.12%
		ROB3 biased studies removed	114,541	350	837	-.30	<.001	[-.31, -.28]	[-.58, -.02]	14,366	<.001	.02	.01	50.88%	41.65%	92.54%
		ROB4 biased studies removed	459,740	670	1,896	-.22	<.001	[-.23, -.21]	[-.50, .06]	80,163	<.001	.02	.01	49.83%	45.29%	95.12%
		ROB7 biased studies removed	176,730	486	1,280	-.24	<.001	[-.26, -.23]	[-.53, .04]	53,743	<.001	.01	.02	44.95%	48.07%	93.01%
		ROB8 biased studies removed	218,664	414	1,161	-.27	<.001	[-.28, -.25]	[-.55, .01]	380,034	<.001	.01	.01	48.13%	49.17%	97.30%
		Unpublished studies removed	358,987	628	1,761	-.23	<.001	[-.24, -.22]	[-.51, .05]	70,735	<.001	.01	.01	45.10%	49.15%	94.25%
		Outliers removed	470,457	719	2,065	-.23	<.001	[-.24, -.22]	[-.50, .04]	36,605	<.001	.01	.01	45.59%	48.85%	94.45%
	**Social resources**	Overall	223,687	401	1,032	.24	<.001	[.22, .26]	[-.04, .53]	20,050	<.001	.02	.01	55.35%	38.41%	93.77%
		ROB1 biased studies removed	222,109	398	1,022	.24	<.001	[.22, .26]	[-.04, .52]	19,809	<.001	.02	.01	55.24%	38.53%	93.76%
		ROB2 biased studies removed	142,539	224	589	.24	<.001	[.22, .26]	[-.04, .52]	11,577	<.001	.02	.01	54.30%	39.76%	94.06%
		ROB3 biased studies removed	76,488	251	612	.27	<.001	[.25, .29]	[.00, .53]	7,957	<.001	.02	.01	54.62%	35.96%	90.58%
		ROB4 biased studies removed	195,297	352	872	.23	<.001	[.21, .24]	[-.06, .51]	16,197	<.001	.02	.01	59.10%	34.55%	93.65%
		ROB7 biased studies removed	93,044	287	679	.25	<.001	[.24, .27]	[-.03, .53]	8,921	<.001	.02	.01	58.50%	33.16%	91.67%
		ROB8 biased studies removed	149,424	263	728	.27	<.001	[.25, .28]	[-.01, .54]	15,989	<.001	.02	.01	55.81%	37.82%	93.63%
		Unpublished studies removed	145,136	354	902	.24	<.001	[.22, .25]	[-.04, .52]	13,097	<.001	.02	.01	54.84%	37.39%	92.23%
		Outliers removed	223,687	401	1,030	.24	<.001	[.22, .26]	[-.04, .52]	18,445	<.001	.02	.01	54.50%	39.03%	93.54%
	**Exposure**	Overall	148,722	144	414	.10	<.001	[.09, .12]	[-.08, .29]	2,599	<.001	.01	.01	47.79%	42.47%	90.26%
		ROB1 biased studies removed	146,601	139	404	.10	<.001	[.09, .12]	[-.09, .29]	2,561	<.001	.01	.01	48.32%	42.18%	90.50%
		ROB2 biased studies removed	105,712	87	274	.09	<.001	[.07, .11]	[-.10, .28]	1,819	<.001	.01	.01	47.73%	43.34%	91.06%
		ROB3 biased studies removed	15,413	29	48	.14	<.001	[.09, .19]	[-.08, .36]	366	<.001	.01	.00	79.10%	12.19%	91.29%
		ROB4 biased studies removed	148,623	143	408	.10	<.001	[.09, .12]	[-.09, .29]	2,596	<.001	.01	.01	47.94%	42.52%	90.46%
		ROB7 biased studies removed	30,449	85	244	.10	<.001	[.08, .12]	[-.09, .29]	1,383	<.001	.01	.01	48.65%	34.28%	82.94%
		ROB8 biased studies removed	101,145	72	215	.13	<.001	[.11, .16]	[-.05, .31]	1,685	<.001	.01	.01	47.92%	43.87%	91.80%
		Unpublished studies removed	71,753	128	348	.10	<.001	[.08, .12]	[-.09, .30]	2,288	<.001	.01	.01	45.65%	40.46%	86.11%
		Outliers removed	148,722	144	414	.10	<.001	[.09, .12]	[-.08, .29]	2,599	<.001	.01	.01	47.79%	42.47%	90.26%

Supplementary Table 13 (cont.)

Outcome	Correlate	ROB criterion	*N*	*k* studies	*n* effects	*r*	*p(r)*	95% CI	90% PI	*Q*	*p(Q)*	τ^2 lv 3	τ^2 lv 2	I^2 lv 3	I^2 lv 2	I^2 total
**Psychological adaptation**																
	**Cultural distance**	Overall	16,001	39	59	-.08	.006	[-.14, -.02]	[-.36, .20]	575	<.001	.03	.00	89.51%	5.29%	94.80%
		ROB1 biased studies removed	16,001	39	59	-.08	.006	[-.14, -.02]	[-.36, .20]	575	<.001	.03	.00	89.51%	5.29%	94.80%
		ROB2 biased studies removed	10,781	26	36	-.09	.005	[-.16, -.03]	[-.36, .17]	341	<.001	.02	.00	82.31%	11.36%	93.68%
		ROB3 biased studies removed	5,762	19	26	-.14	.003	[-.22, -.05]	[-.45, .18]	120	<.001	.03	.00	91.53%	0.00%	91.53%
		ROB4 biased studies removed	16,001	39	59	-.08	.006	[-.14, -.02]	[-.36, .20]	575	<.001	.03	.00	89.51%	5.29%	94.80%
		ROB7 biased studies removed	12,891	31	42	-.08	.010	[-.15, -.02]	[-.36, .19]	399	<.001	.02	.00	85.49%	8.51%	94.00%
		ROB8 biased studies removed	8,973	26	36	-.09	.016	[-.17, -.02]	[-.40, .21]	224	<.001	.03	.00	94.72%	0.00%	94.72%
		Unpublished studies removed	15,380	35	53	-.08	<.001	[-.13, -.04]	[-.30, .14]	465	<.001	.02	.00	83.14%	9.04%	92.18%
		Outliers removed	16,001	39	59	-.08	.006	[-.14, -.02]	[-.36, .20]	575	<.001	.03	.00	89.51%	5.29%	94.80%
**Socio-cultural adaptation**																
	**Cultural distance**	Overall	20,900	98	186	-.16	<.001	[-.20, -.13]	[-.45, .13]	1,620	<.001	.01	.02	39.68%	48.11%	87.79%
		ROB1 biased studies removed	20,780	97	185	-.16	<.001	[-.20, -.13]	[-.45, .13]	1,616	<.001	.01	.02	39.68%	48.14%	87.82%
		ROB2 biased studies removed	9,908	59	107	-.18	<.001	[-.22, -.13]	[-.49, .14]	1,056	<.001	.02	.02	37.44%	50.54%	87.98%
		ROB3 biased studies removed	9,662	42	70	-.18	<.001	[-.23, -.14]	[-.42, .05]	390	<.001	.01	.01	32.34%	51.20%	83.55%
		ROB4 biased studies removed	20,900	98	186	-.16	<.001	[-.20, -.13]	[-.45, .13]	1,620	<.001	.01	.02	39.68%	48.11%	87.79%
		ROB7 biased studies removed	13,828	67	117	-.14	<.001	[-.18, -.11]	[-.38, .10]	622	<.001	.01	.01	29.17%	53.31%	82.49%
		ROB8 biased studies removed	16,760	71	153	-.16	<.001	[-.19, -.12]	[-.42, .10]	1,153	<.001	.01	.02	31.96%	53.93%	85.89%
		Unpublished studies removed	19,328	90	172	-.17	<.001	[-.21, -.14]	[-.46, .12]	1,471	<.001	.01	.02	36.65%	51.17%	87.82%
		Outliers removed	20,900	98	185	-.16	<.001	[-.19, -.13]	[-.44, .12]	1,395	<.001	.01	.02	39.55%	47.43%	86.97%

Supplementary Table 13 (cont.)

Outcome	Correlate	ROB criterion	*N*	*k* studies	*n* effects	*r*	*p(r)*	95% CI	90% PI	*Q*	*p(Q)*	τ^2 lv 3	τ^2 lv 2	I^2 lv 3	I^2 lv 2	I^2 total
**Socio-cultural adaptation**																
	**Stressors**	Overall	52,149	210	457	-.26	<.001	[-.28, -.23]	[-.59, .07]	20,435	<.001	.02	.02	41.08%	53.11%	94.19%
		ROB1 biased studies removed	51,324	206	450	-.26	<.001	[-.28, -.23]	[-.59, .07]	14,472	<.001	.02	.02	40.06%	53.42%	93.48%
		ROB2 biased studies removed	25,351	116	258	-.27	<.001	[-.31, -.24]	[-.60, .05]	7,846	<.001	.02	.02	39.36%	53.52%	92.89%
		ROB3 biased studies removed	23,481	92	196	-.28	<.001	[-.32, -.23]	[-.63, .08]	6,621	<.001	.02	.02	50.16%	44.14%	94.30%
		ROB4 biased studies removed	51,262	202	436	-.25	<.001	[-.28, -.23]	[-.57, .06]	11,403	<.001	.02	.02	42.06%	50.80%	92.86%
		ROB7 biased studies removed	37,419	144	298	-.24	<.001	[-.26, -.21]	[-.54, .07]	8,047	<.001	.01	.02	36.50%	55.84%	92.34%
		ROB8 biased studies removed	43,762	173	379	-.26	<.001	[-.29, -.24]	[-.59, .06]	18,252	<.001	.02	.02	49.66%	44.52%	94.17%
		Unpublished studies removed	43,728	171	368	-.26	<.001	[-.29, -.23]	[-.58, .06]	9,515	<.001	.02	.02	40.78%	52.14%	92.92%
		Outliers removed	51,567	208	451	-.25	<.001	[-.27, -.23]	[-.56, .06]	6,626	<.001	.01	.02	34.74%	56.89%	91.62%
	**Social resources**	Overall	44,535	180	599	.27	<.001	[.24, .29]	[-.04, .57]	92,939	<.001	.02	.01	61.89%	35.92%	97.80%
		ROB1 biased studies removed	44,352	178	593	.27	<.001	[.25, .30]	[-.03, .58]	92,441	<.001	.02	.01	62.00%	35.80%	97.81%
		ROB2 biased studies removed	17,731	86	310	.28	<.001	[.24, .32]	[-.04, .60]	40,674	<.001	.03	.01	68.68%	27.57%	96.25%
		ROB3 biased studies removed	32,330	120	366	.28	<.001	[.25, .32]	[-.02, .59]	61,984	<.001	.02	.01	66.49%	31.87%	98.36%
		ROB4 biased studies removed	41,820	168	579	.26	<.001	[.23, .28]	[-.04, .56]	90,503	<.001	.02	.01	61.37%	36.37%	97.74%
		ROB7 biased studies removed	33,136	127	395	.27	<.001	[.24, .30]	[-.05, .59]	68,920	<.001	.02	.01	64.40%	34.06%	98.45%
		ROB8 biased studies removed	36,175	141	499	.28	<.001	[.25, .30]	[-.01, .56]	43,925	<.001	.02	.01	61.49%	32.65%	94.14%
		Unpublished studies removed	36,952	150	469	.27	<.001	[.24, .29]	[-.02, .56]	73,437	<.001	.02	.01	54.79%	42.93%	97.73%
		Outliers removed	44,212	178	595	.26	<.001	[.24, .29]	[-.03, .55]	11,791	<.001	.02	.01	54.74%	36.31%	91.05%
	**Exposure**	Overall	17,290	79	246	.14	<.001	[.11, .18]	[-.16, .44]	1,966	<.001	.01	.02	35.65%	51.47%	87.13%
		ROB1 biased studies removed	17,290	79	246	.14	<.001	[.11, .18]	[-.16, .44]	1,966	<.001	.01	.02	35.65%	51.47%	87.13%
		ROB2 biased studies removed	7,852	46	141	.16	<.001	[.11, .21]	[-.16, .49]	1,316	<.001	.02	.02	43.16%	44.95%	88.12%
		ROB3 biased studies removed	6,873	19	38	.12	.001	[.05, .20]	[-.19, .43]	290	<.001	.01	.02	25.70%	64.13%	89.83%
		ROB4 biased studies removed	17,090	77	241	.14	<.001	[.11, .18]	[-.15, .44]	1,905	<.001	.01	.02	34.24%	52.62%	86.86%
		ROB7 biased studies removed	12,666	51	164	.11	<.001	[.07, .14]	[-.16, .37]	1,136	<.001	.01	.02	23.24%	61.88%	85.12%
		ROB8 biased studies removed	11,501	43	156	.14	<.001	[.10, .18]	[-.11, .39]	952	<.001	.01	.02	27.29%	56.01%	83.30%
		Unpublished studies removed	15,874	71	214	.15	<.001	[.11, .18]	[-.16, .45]	1,812	<.001	.01	.02	34.33%	53.42%	87.76%
		Outliers removed	17,290	79	246	.14	<.001	[.11, .18]	[-.16, .44]	1,966	<.001	.01	.02	35.65%	51.47%	87.13%

Supplementary Table 14.

Multilevel Egger's regressions for the average associations of main categories of correlates with psychological and socio-cultural adaptation.

Outcome	Correlate Category	*N*	*k* studies	*n* effects	intercept	moderator	*p*(intercept)	*p*(moderator)	95% CI (intercept)	95% CI (moderator)	*Q*	*p(Q)*	τ^2 lv3	τ^2 lv2
**Psychological adaptation**														
	Stressors	470,588	719	2,073	-.39	2.50	<.001	<.001	[-.42, -.36]	[2.09, 2.91]	341,294	<.001	.02	.01
	Social resources	223,540	399	1,029	.39	-2.30	<.001	<.001	[.35, .43]	[-2.86, -1.74]	18,472	<.001	.02	.01
	Exposure	148,722	144	414	.11	-.12	<.001	.698	[.07, .16]	[-.74, .49]	2,580	<.001	.01	.01
	Cultural distance	16,001	39	59	-.15	.91	.074	.395	[-.31, .01]	[-1.18, 3.00]	568	<.001	.03	.00
**Socio-cultural adaptation**														
	Stressors	52,149	210	457	-.57	4.60	<.001	<.001	[-.64, -.50]	[3.60, 5.60]	11,391	<.001	.02	.02
	Social resources	44,425	179	598	.89	-9.20	<.001	<.001	[.82, .96]	[-10.19, -8.21]	12,164	<.001	.04	.00
	Exposure	17,290	79	246	.47	-4.15	<.001	<.001	[.36, .59]	[-5.56, -2.73]	1,844	<.001	.02	.01
	Cultural distance	20,900	98	186	-.44	3.66	<.001	<.001	[-.55, -.33]	[2.26, 5.06]	1,538	<.001	.02	.01

Supplementary Table 15.
Multilevel PET-PEESE analyses for the average associations of main categories of correlates with psychological and socio-cultural adaptation.

Outcome	Model	Predictor	*N*	*k* studies	*n* effects	*r*	*p*	95% CI	τ^2 lv 3	τ^2 lv 2
		**Sample-size variant of PET-PEESE (Pustejovsky & Rodgers)**								
Psychological adaptation	Precision-effect test (PET)	**Stressors**	437656	719	2073				.01	.01
		Intercept				-.15	<.001	[-.17, -.12]		
		Bias				-1.24	<.001	[-1.60, -.89]		
		**Social resources**	218040	401	1032				.02	.01
		Intercept				.22	<.001	[.17, .26]		
		Bias				.30	.280	[-.24, .84]		
		**Exposure**	145119	144	414				.01	.01
		Intercept				.07	<.001	[.03, .12]		
		Bias				.40	.185	[-.19, .98]		
		**Cultural distance**	15362	39	59				.02	.00
		Intercept				**-.12**	**.121**	**[-.27, .03]**		
		Bias				.52	.593	[-1.38, 2.41]		
	Precision-effect estimate with standard errors (PEESE)	**Stressors**	437656	719	2073				.01	.01
		Intercept				**-.19**	**<.001**	**[-.21, -.18]**		
		Bias				-6.51	<.001	[-8.60, -4.41]		
		**Social resources**	218040	401	1032				.02	.01
		Intercept				**.23**	**<.001**	**[.21, .26]**		
		Bias				.79	.616	[-2.30, 3.88]		
		**Exposure**	145119	144	414				.01	.01
		Intercept				**.08**	**<.001**	**[.06, .11]**		
		Bias				3.19	.103	[-.64, 7.02]		
		**Cultural distance**	15362	39	59				.02	.00
		Intercept				-.11	.023	[-.20, -.01]		
		Bias				4.08	.496	[-7.68, 15.85]		
Socio-cultural adaptation	Precision-effect test (PET)	**Stressors**	49574	210	457				.02	.02
		Intercept				-.22	<.001	[-.30, -.14]		
		Bias				-.44	.395	[-1.47, .58]		
		**Social resources**	42928	180	599				.02	.01
		Intercept				.33	<.001	[.25, .42]		
		Bias				-.95	.099	[-2.07, .18]		
		**Exposure**	17082	79	246				.01	.02
		Intercept				.21	<.001	[.09, .33]		
		Bias				-.86	.221	[-2.23, .52]		
		**Cultural distance**	19348	98	186				.01	.01
		Intercept				**-.05**	**.409**	**[-.15, .06]**		
		Bias				-1.41	.035	[-2.73, -.10]		
	Precision-effect estimate with standard errors (PEESE)	**Stressors**	49574	210	457				.02	.02
		Intercept				**-.24**	**<.001**	**[-.28, -.19]**		
		Bias				-2.33	.459	[-8.49, 3.83]		
		**Social resources**	42928	180	599				.02	.01
		Intercept				**.30**	**<.001**	**[.25, .35]**		
		Bias				-5.80	.094	[-12.59, .98]		
		**Exposure**	17082	79	246				.01	.02
		Intercept				**.18**	**<.001**	**[.11, .25]**		
		Bias				-5.14	.187	[-12.79, 2.50]		
		**Cultural distance**	19348	98	186				.01	.01
		Intercept				-.10	.001	[-.15, -.04]		
		Bias				-8.91	.016	[-16.18, -1.64]		

Supplementary Table 15 (cont.)

Outcome	Model	Predictor	*N*	*k* studies	*n* effects	*r*	*p*	95% CI	τ^2 lv 3	τ^2 lv 2
		**Variance-based variant of PET-PEESE (Stanley & Doucouliagos)**								
Psychological adaptation	Precision-effect test (PET)	**Stressors**	437656	719	2073				.02	.01
		Intercept				-.39	<.001	[-.42, -.36]		
		Bias				2.50	<.001	[2.09, 2.91]		
		**Social resources**	218040	401	1032				.02	.01
		Intercept				.39	<.001	[.35, .43]		
		Bias				-2.30	<.001	[-2.85, -1.74]		
		**Exposure**	145119	144	414				.01	.01
		Intercept				.11	<.001	[.07, .16]		
		Bias				-.12	.698	[-.74, .49]		
		**Cultural distance**	15362	39	59				.03	.00
		Intercept				-.15	.074	[-.31, .01]		
		Bias				.91	.395	[-1.18, 3.00]		
	Precision-effect estimate with standard errors (PEESE)	**Stressors**	437656	719	2073				.02	.01
		Intercept				**-.29**	**<.001**	**[-.31, -.27]**		
		Bias				12.26	<.001	[9.82, 14.71]		
		**Social resources**	218040	401	1032				.02	.01
		Intercept				**.30**	**<.001**	**[.28, .32]**		
		Bias				-11.92	<.001	[-15.16, -8.69]		
		**Exposure**	145119	144	414				.01	.01
		Intercept				**.11**	**<.001**	**[.08, .13]**		
		Bias				-.52	.804	[-4.60, 3.56]		
		**Cultural distance**	15362	39	59				.03	.00
		Intercept				**-.12**	**.015**	**[-.21, -.02]**		
		Bias				6.41	.335	[-6.61, 19.43]		
Socio-cultural adaptation	Precision-effect test (PET)	**Stressors**	49574	210	457				.02	.02
		Intercept				-.57	<.001	[-.64, -.50]		
		Bias				4.60	<.001	[3.60, 5.60]		
		**Social resources**	42928	180	599				.03	.00
		Intercept				.89	<.001	[.81, .96]		
		Bias				-9.12	<.001	[-10.11, -8.13]		
		**Exposure**	17082	79	246				.02	.01
		Intercept				.47	<.001	[.36, .59]		
		Bias				-4.15	<.001	[-5.56, -2.73]		
		**Cultural distance**	19348	98	186				.02	.01
		Intercept				-.44	<.001	[-.55, -.33]		
		Bias				3.66	<.001	[2.26, 5.06]		
	Precision-effect estimate with standard errors (PEESE)	**Stressors**	49574	210	457				.02	.02
		Intercept				**-.39**	**<.001**	**[-.44, -.35]**		
		Bias				26.41	<.001	[19.40, 33.42]		
		**Social resources**	42928	180	599				.02	.01
		Intercept				**.48**	**<.001**	**[.43, .52]**		
		Bias				-40.37	<.001	[-46.77, -33.98]		
		**Exposure**	17082	79	246				.02	.02
		Intercept				**.30**	**<.001**	**[.24, .37]**		
		Bias				-23.09	<.001	[-31.26, -14.92]		
		**Cultural distance**	19348	98	186				.02	.01
		Intercept				**-.29**	**<.001**	**[-.35, -.23]**		
		Bias				20.56	<.001	[12.62, 28.50]		

Supplementary Table 16.

Results before and after correcting for *p*-hacking using the RTMA method for the average associations of main categories of correlates with psychological and socio-cultural adaptation. As per Mathur's recommendation[1028], mode r and τ from RTMA model are reported. Bold font indicates cases in which the corrected effect is larger than the uncorrected effect, suggesting that the core results were underestimated. Because RTMA does not account for correlated effects, the above analysis ignores the hierarchical structure of the data. For the sake of comparability, uncorrected effects were obtained using a standard random effects model without specifying a three-level structure, hence minor discrepancies from the results reported in the main manuscript. Please note that this analysis violates RTMA's assumptions, as indicated by the diagnostic quantile-quantile plots, and the results should be taken with extreme caution. *With convergent results the value of $\hat{r}$ should not exceed 1.01, and the effective sample size n_{eff} should be in the hundreds. However, for cultural distance and psychological adaptation these estimates were $\hat{r}$ = 2.92, n_{eff} = 2.27, and for stressors and socio-cultural adaptation $\hat{r}$ = 1.13, n_{eff} = 29.90, suggesting that these results are not valid and should not be interpreted.

**Outcome**	**Correlate category**	**Uncorrected estimates**		**Corrected estimates**		**Change (absolute value)**	
		r	**τ**	***r***	**τ**	***Δr***	***Δτ***
Psychological Adaptation							
	Cultural distance*	-.09	.14	-.23	.21	**.14**	**.07**
	Exposure	.10	.11	.07	.09	.03	.02
	Resources	.22	.17	.37	.23	**.15**	**.06**
	Stressors	-.23	.17	-.17	.12	.06	.05
Socio-cultural Adaptation							
	Cultural distance	-.16	.17	-.51	.28	**.35**	**.11**
	Exposure	.12	.18	.06	.14	.06	.04
	Resources	.26	.19	.15	.10	.11	.09
	Stressors*	-.25	.20	-.25	.20	.00	.00

Supplementary Table 17.
PRISMA 2020 checklist. This table was created using the PRISMA 2020 template (https://www.prisma-statement.org/prisma-2020-checklist) developed by Page, M.J, McKenzie, J.E., Bossuyt, P.M., Boutron, I., Hoffmann, T.C., Mulrow, C.D., et al. The PRISMA 2020 statement: an updated guideline for reporting systematic reviews. *BMJ* **372**, n71 (2021). https:/doi.org/10.1136/bmj.n71.[1027] The template is licensed under CC BY 4.0. The license is available via https://creativecommons.org/licenses/by/4.0/ .

Section and Topic	Item #	Checklist item	Location where item is reported
**TITLE**			
Title	1	Identify the report as a systematic review.	Title
**ABSTRACT**			
Abstract	2	See the PRISMA 2020 for Abstracts checklist.	Abstract
**INTRODUCTION**			
Rationale	3	Describe the rationale for the review in the context of existing knowledge.	Introduction, 2nd paragraph
Objectives	4	Provide an explicit statement of the objective(s) or question(s) the review addresses.	Introduction, last paragraph
**METHODS**			
Eligibility criteria	5	Specify the inclusion and exclusion criteria for the review and how studies were grouped for the syntheses.	Subsection Eligibility Criteria
Information sources	6	Specify all databases, registers, websites, organisations, reference lists and other sources searched or consulted to identify studies. Specify the date when each source was last searched or consulted.	Subsection Literature Search and Prisma graph (Fig.9)
Search strategy	7	Present the full search strategies for all databases, registers and websites, including any filters and limits used.	Supplementary Methods
Selection process	8	Specify the methods used to decide whether a study met the inclusion criteria of the review, including how many reviewers screened each record and each report retrieved, whether they worked independently, and if applicable, details of automation tools used in the process.	Subsection Data Screening, Coding; Supplementary Methods
Data collection process	9	Specify the methods used to collect data from reports, including how many reviewers collected data from each report, whether they worked independently, any processes for obtaining or confirming data from study investigators, and if applicable, details of automation tools used in the process.	Subsection Coding; Supplementary Methods
Data items	10a	List and define all outcomes for which data were sought. Specify whether all results that were compatible with each outcome domain in each study were sought (e.g. for all measures, time points, analyses), and if not, the methods used to decide which results to collect.	Subsection Eligibility Criteria; Supplementary Table 12; Preregistration, section Data Items
	10b	List and define all other variables for which data were sought (e.g. participant and intervention characteristics, funding sources). Describe any assumptions made about any missing or unclear information.	Supplementary Data (OSF file), tab Variable List; Preregistration,

Section and Topic	Item #	Checklist item	Location where item is reported
			section Data Items
Study risk of bias assessment	11	Specify the methods used to assess risk of bias in the included studies, including details of the tool(s) used, how many reviewers assessed each study and whether they worked independently, and if applicable, details of automation tools used in the process.	Subsection Risk of Bias and Sensitivity Analyses; Supplementary Methods, Risk of Bias Assessment; Supplementary Table 13
Effect measures	12	Specify for each outcome the effect measure(s) (e.g. risk ratio, mean difference) used in the synthesis or presentation of results.	Supplementary data (OSF file), tab Data
Synthesis methods	13a	Describe the processes used to decide which studies were eligible for each synthesis (e.g. tabulating the study intervention characteristics and comparing against the planned groups for each synthesis (item #5)).	Subsection Eligibility Criteria; Supplementary Methods
	13b	Describe any methods required to prepare the data for presentation or synthesis, such as handling of missing summary statistics, or data conversions.	Supplementary Methods
	13c	Describe any methods used to tabulate or visually display results of individual studies and syntheses.	Captions of Figure 1—8 and Table 1. Note that individual studies were not tabulated in the main paper due to high *k*.
	13d	Describe any methods used to synthesize results and provide a rationale for the choice(s). If meta-analysis was performed, describe the model(s), method(s) to identify the presence and extent of statistical heterogeneity, and software package(s) used.	Subsection Analytical Strategy
	13e	Describe any methods used to explore possible causes of heterogeneity among study results (e.g. subgroup analysis, meta-regression).	Section Results, especially: Subsection Heterogeneity and further subsections.
	13f	Describe any sensitivity analyses conducted to assess robustness of the synthesized results.	Subsection Risk of Bias and Sensitivity Analyses
Reporting bias assessment	14	Describe any methods used to assess risk of bias due to missing results in a synthesis (arising from reporting biases).	Subsection Risk of Bias and Sensitivity Analyses; Supplementary Methods, Risk of Bias Assessment
Certainty assessment	15	Describe any methods used to assess certainty (or confidence) in the body of evidence for an outcome.	Subsection Risk of Bias and Sensitivity Analyses

Section and Topic	Item #	Checklist item	Location where item is reported
**RESULTS**			
Study selection	16a	Describe the results of the search and selection process, from the number of records identified in the search to the number of studies included in the review, ideally using a flow diagram.	PRISMA graph, Figure 9
	16b	Cite studies that might appear to meet the inclusion criteria, but which were excluded, and explain why they were excluded.	Supplementary Methods
Study characteristics	17	Cite each included study and present its characteristics.	Data (OSF file), tab Data. Note that individual studies were not described in the main manuscript due to high *k*.
Risk of bias in studies	18	Present assessments of risk of bias for each included study.	Subsection Risk of Bias and Sensitivity Analyses; Supplementary Table 13
Results of individual studies	19	For all outcomes, present, for each study: (a) summary statistics for each group (where appropriate) and (b) an effect estimate and its precision (e.g. confidence/credible interval), ideally using structured tables or plots.	Results, all subsections; Supplementary Table 1-11, 13-16
Results of syntheses	20a	For each synthesis, briefly summarise the characteristics and risk of bias among contributing studies.	Subsection Risk of Bias and Sensitivity Analyses; Supplementary Table 13
	20b	Present results of all statistical syntheses conducted. If meta-analysis was done, present for each the summary estimate and its precision (e.g. confidence/credible interval) and measures of statistical heterogeneity. If comparing groups, describe the direction of the effect.	Results, all subsections; Supplementary Table 1-11, 13-16
	20c	Present results of all investigations of possible causes of heterogeneity among study results.	Results, all subsections; Supplementary Table 1-11, 13-16
	20d	Present results of all sensitivity analyses conducted to assess the robustness of the synthesized results.	Subsection Risk of Bias and Sensitivity Analyses, Supplementary Table 13
Reporting biases	21	Present assessments of risk of bias due to missing results (arising from reporting biases) for each synthesis assessed.	Subsection Risk of Bias and Sensitivity Analyses; Supplementary Table 13

Section and Topic	Item #	Checklist item	Location where item is reported
Certainty of evidence	22	Present assessments of certainty (or confidence) in the body of evidence for each outcome assessed.	Subsection Risk of Bias and Sensitivity Analyses; Supplementary Table 13; Publication Bias and P-Hacking, Supplementary Table 14-16
**DISCUSSION**			
Discussion	23a	Provide a general interpretation of the results in the context of other evidence.	Discussion, several paragraphs
	23b	Discuss any limitations of the evidence included in the review.	Discussion, several paragraphs
	23c	Discuss any limitations of the review processes used.	Discussion, several paragraphs
	23d	Discuss implications of the results for practice, policy, and future research.	Discussion, last paragraph
**OTHER INFORMATION**			
Registration and protocol	24a	Provide registration information for the review, including register name and registration number, or state that the review was not registered.	Subsection Transparency of Methods and Materials
	24b	Indicate where the review protocol can be accessed, or state that a protocol was not prepared.	Subsection Transparency of Methods and Materials
	24c	Describe and explain any amendments to information provided at registration or in the protocol.	Subsection Transparency of Methods and Materials
Support	25	Describe sources of financial or non-financial support for the review, and the role of the funders or sponsors in the review.	Acknowledgements
Competing interests	26	Declare any competing interests of review authors.	Competing Interests Statement
Availability of data, code and other materials	27	Report which of the following are publicly available and where they can be found: template data collection forms; data extracted from included studies; data used for all analyses; analytic code; any other materials used in the review.	Subsection Transparency of Methods and Materials

Supplementary References

Reference List of Reports Included in The Meta-Analysis

1. Abarca, G., Jr. Social Stressors and Severity of Somatic Symptoms among Latina Immigrant Women. (ProQuest Dissertations and Theses, 2020).
2. Abdul Malek, M., Budhwar, P. & Reiche, B. S. Sources of support and expatriation: a multiple stakeholder perspective of expatriate adjustment and performance in Malaysia. *The International Journal of Human Resource Management* **26**, 258-276 (2015).
3. Abdul-Malak, Y. Healthy Immigrants? Exploring Depressive Symptoms Among Caribbean and Mexican Immigrants. *Journal of Racial and Ethnic Health Disparities* **7**, 488-497 (2020).
4. Acarturk, C.*, et al.* Prevalence and predictors of common mental disorders among Syrian refugees in Istanbul, Turkey: a cross-sectional study. *Social psychiatry and psychiatric epidemiology* **56**, 475-484 (2021).
5. Acharya, L., Jin, L. & Collins, W. College life is stressful today–Emerging stressors and depressive symptoms in college students. *Journal of American college health* **66**, 655-664 (2018).
6. Adedeji, A. & Bullinger, M. Subjective integration and quality of life of Sub-Saharan African migrants in Germany. *Public Health* **174**, 134-144 (2019).
7. Adewale, O. R. Psychological Stress of Nigerian Immigrants in the Greater Toronto Area. (ProQuest Dissertations and Theses, 2017).
8. Afsharian, A.*, et al.* Refugees at Work: The Preventative Role of Psychosocial Safety Climate against Workplace Harassment, Discrimination and Psychological Distress. *International journal of environmental research and public health* **18**(2021).
9. Agadjanian, V., Oh, B. & Menjivar, C. (Il)legality and psychosocial well-being: Central Asian migrant women in Russia. *Journal of ethnic and migration studies* **48**, 53-73 (2022).
10. Ahmad, I. & Smetana, J. Palestinian refugee youth in Jordan: parental practices, neighborhood cohesion and assistance, and adolescent wellbeing. *International journal of environmental research and public health* **18**, 3649 (2021).

11. Ahmed, S. & Rasmussen, A. Changes in social status and postmigration mental health among West African immigrants. *American Journal of Orthopsychiatry* **90**, 171 (2020).

12. Ahmmad, Z., Wen, M. & Li, K. Self-rated health disparities among Asian Americans: mediating roles of education level and household income. *Journal of Immigrant and Minority Health* **23**, 583-590 (2021).

13. Ahorsu, D. K.*, et al.* COVID-19-Related Variables and Its Association with Anxiety and Suicidal Ideation: Differences Between International and Local University Students in Taiwan. *Psychology research and behavior management* **14**, 1857-1866 (2021).

14. Aikawa, M. & Kleyman, K. Immigration, coping, and well-being: Implications for communities' roles in promoting the well-being of immigrants and refugees. *Journal of Prevention & Intervention in the Community* **49**, 81-92 (2021).

15. Akhal, K. & Liu, S. Cultural intelligence effects on expatriates' adjustment and turnover intentions in Mainland China. *Management Research Review* **42**, 818-836 (2019).

16. Akhimien, O. G. & Adekunle, S. A. Cultural distance and expatriates' psychological adjustment: the moderating role of perceived social supports. *International Journal of Organizational Analysis* **31**, 1841-1866 (2021).

17. Akhtar, M. & Herwig, B. K. Psychosomatic distress symptoms among international students in Germany: Role of academic stress and socio-demographic factors. *Journal of the Pakistan Medical Association* **70**, 1119-1124 (2020).

18. Akhtar, M. & Kroener-Herwig, B. Coping Styles and Socio-demographic Variables as Predictors of Psychological Well-Being among International Students Belonging to Different Cultures. *Current Psychology* **38**, 618-626 (2017).

19. Akhtar, N., Pratt, C. B. & Bo, S. Factors in the cross-cultural adaptation of African students in Chinese universities. *Journal of Research in International Education* **14**, 98-113 (2015).

20. Al Mahrouqi, A. Cross-Cultural Communication and the Adjustment of Western and Non-Western Expatriates in Multicultural Companies: Investigating Operations in Oman. (University of Glasgow, 2018).

21. Al-Sharideh, K. A. & Goe, W. R. Ethnic communities within the university: An examination of factors influencing the personal adjustment of international students. *Research in Higher Education* **39**, 699-725 (1998).

22. Alam, M. D., Lu, J., Ni, L., Hu, S. & Xu, Y. Psychological Outcomes and Associated Factors Among the International Students Living in China During the COVID-19 Pandemic. *Frontiers in psychiatry* **12**, 707342 (2021).

23. Alcantara, C., Chen, C. N. & Alegria, M. Do post-migration perceptions of social mobility matter for Latino immigrant health? *Social Science & Medicine* **101**, 94-106 (2014).

24. Alcantara, C., Chen, C. N. & Alegria, M. Transnational ties and past-year major depressive episodes among Latino immigrants. *Cultural Diversity and Ethnic Minority Psychology* **21**, 486-495 (2015).

25. Aldawsari, N. F., Adams, K.S., Grimes, L. E. & Kohn, S. The Effects of Cross-Cultural Competence and Social Support on International Students' Psychological Adjustment: Autonomy and Environmental Mastery. *Journal of International Students* **8.0**, 901-924 (2018).

26. Alduraidi, H. Social Determinants of Health-related Quality of Life in Palestinian Refugees in Jordan. (University of California, 2016).

27. Alemi, Q., James, S., Siddiq, H. & Montgomery, S. Correlates and Predictors of Psychological Distress among Afghan Refugees in San Diego County. *International journal of culture and mental health* **8**, 274-288 (2015).

28. Alemi, Q. & Stempel, C. Discrimination and distress among Afghan refugees in northern California: The moderating role of pre- and post-migration factors. *PLoS One* **13**, e0196822 (2018).

29. Alemi, Q., *et al.* Impact of Postmigration Living Difficulties on the Mental Health of Afghan Migrants Residing in Istanbul. *International Journal of Population Research* **2016**, 1-8 (2016).

30. Alemu, A. M. & Cordier, J. Factors influencing international student satisfaction in Korean universities. *International Journal of Educational Development* **57**, 54-64 (2017).

31. Algarni, M. A., *et al.* Perception of Threat and Psychological Impact of COVID-19 among Expatriates in Makkah Region, Saudi Arabia. *International Journal of Environmental Research and Public Health*

18(2021).

32. Alharbi, E. Away from home: A mixed-methods study of well-being of international students in the UK. (Cardiff University, 2020).

33. Ali, A., Van der Zee, K. & Sanders, G. Determinants of intercultural adjustment among expatriate spouses. *International Journal of Intercultural Relations* **27**, 563-580 (2003).

34. Ali, A. S. M. & Ahmad, R. The impact of social media on international students: Cultural and academic adaptation. *Journal of Engineering and Applied Sciences* **13**, 546-551 (2018).

35. Ali, S. Z., Mubashar, T., Khan, S. & Akhtar, T. Psychosocial Aspects of Adaptation of International Students in Pakistan. *Pakistan Journal of Psychological Research* **36**, 135-156 (2021).

36. Alpay, E. H*., et al.* The effects of COVID-19 continuous traumatic stress on mental health: The case of Syrian refugees in Turkey. *Traumatology* **27**, 375-387 (2021).

37. Alsaad, K. S. M. Psychosocial and Mental Health Challenges of International Students compared to British Students in UK Universities. (University of Bedfordshire, 2017).

38. Amado, S., Snyder, H. R. & Gutchess, A. Mind the Gap: The Relation Between Identity Gaps and Depression Symptoms in Cultural Adaptation. *Frontiers in Psychology* **11**, 1156 (2020).

39. Amini, S. S. & Nguyen, A.-M. D. Muslim and Jewish Immigrants' Adjustment: The Role of Religious-American Harmony, Religious-American Identity Centrality, and Discrimination. *Journal of Cross-Cultural Psychology* **52**, 259-274 (2021).

40. Amirkhan, J. H. & Velasco, S. E. Stress Overload and the New Nightmare for Dreamers. *Journal of American College Health* (2020).

41. Amit, K. Determinants of Life Satisfaction Among Immigrants from Western Countries and from the FSU in Israel. *Social Indicators Research* **96**, 515-534 (2010).

42. Ammigand, R., Drexlerd, M. L., Williamson, A. A. & Guerra, N. G. Prevalence and Correlates of Depressive Symptoms Among International Students: Implications for University Support Offices. *Journal of International Students* **9**, 129-149 (2019).

43. Amoah, P. A. & Mok, E. W. C. COVID-19 and well-being of non-local students: Implications for

international higher education governance. *Higher Education Policy* **35**, 651-672 (2022).

44. An, J. Y., Cha, S., Moon, H., Ruggiero, J. S. & Jang, H. Factors Affecting Job Satisfaction of Immigrant Korean Nurses. *Journal of Transcultural Nursing* **27**, 126-135 (2016).

45. An, J. Y., Moon, H. & Cha, S. Health literacy, depression, and stress among Chinese immigrants in South Korea. *Public Health Nursing* **36**, 603-614 (2019).

46. An, T., Hamamura, T., Kishimoto, T. & Mearns, J. Negative Mood Regulation Expectancies Moderate the Effects of Acculturative Stress on Affective Symptoms Among Chinese International Students in Japan. *Japanese Psychological Research* (2022).

47. Anderson, J. R. & Guan, Y. Implicit acculturation and the academic adjustment of Chinese student sojourners in Australia. *Australian Psychologist* **53**, 444-453 (2018).

48. Anderson, Y. H. East Asian International Students' Interdependent Happiness: The Role of Acculturative Stress, Dialectical Thinking, and Collectivistic Coping. (Purdue University, 2018).

49. Anderzén, I. & Arnetz, B. B. Psychophysiological reactions during the year of a foreign assignment: results of a controlled longitudinal study. *Psychotherapy and Psychosomatics* **68**, 67-75 (1999).

50. Andresen, M., Goldmann, P. & Volodina, A. Do Overwhelmed Expatriates Intend to Leave? The Effects of Sensory Processing Sensitivity, Stress, and Social Capital on Expatriates' Turnover Intention. *European Management Review* **15**, 315-328 (2018).

51. Andrews, C. Culture's Role in Immigrant Health: How Cultural Consonance Shapes Diabetes and Depression among Mexican Women in Alabama. (ProQuest Dissertations and Theses, 2018).

52. Anjara, S. G., Nellums, L. B., Bonetto, C. & Van Bortel, T. Stress, health and quality of life of female migrant domestic workers in Singapore: a cross-sectional study. *BMC Womens Health* **17**, 98 (2017).

53. Arellano-Morales, L., Liang, C. T., Ruiz, L. & Rios-Oropeza, E. Perceived racism, gender role conflict, and life satisfaction among Latino day laborers. *Journal of Latina/o Psychology* **4**, 32-42 (2016).

54. Arenas, E., Yahirun, J., Teruel, G., Rubalcava, L. & Gaitán-Rossi, P. Gender, family separation, and negative emotional well-being among recent Mexican migrants. *Journal of Marriage and Family* **83**, 1401-1419 (2021).

55. Arevalo, S. P., Tucker, K. L. & Falcon, L. M. Beyond cultural factors to understand immigrant mental health: Neighborhood ethnic density and the moderating role of pre-migration and post-migration factors. *Social Science & Medicine* **138**, 91-100 (2015).

56. Aroian, K., Uddin, N. & Blbas, H. Longitudinal study of stress, social support, and depression in married Arab immigrant women. *Health care for women international* **38**, 100-117 (2017).

57. Aryee, S. & Stone, R. J. Work experiences, work adjustment and psychological well-being of expatriate employees in Hong Kong. *The International Journal of Human Resource Management* **7**, 150-164 (1996).

58. Asfar, D., Born, M. P., Oostrom, J. K. & van Vugt, M. Psychological individual differences as predictors of refugees' local language proficiency. *European Journal of Social Psychology* **49**, 1385-1400 (2019).

59. Ataca, B. & Berry, J. W. Psychological, sociocultural, and marital adaptation of Turkish immigrant couples in Canada. *International Journal of Psychology* **37**, 13-26 (2002).

60. Atri, A., Sharma, M. & Cottrell, R. Role of social support, hardiness, and acculturation as predictors of mental health among international students of Asian Indian origin. *International Quarterly of Community Health Education* **27**, 59-73 (2007).

61. Atrooz, F*., et al.* Displacement and Isolation: Insights from a Mental Stress Survey of Syrian Refugees in Houston, Texas, USA. *International Journal of Environmental Research and Public Health* **19**(2022).

62. Atteraya, M. S. Acculturation Stressors and Academic Adjustment among Nepalese Students in South Korean Higher Education Institutions. *International Journal of Environmental Research and Public Health* **18**(2021).

63. Ayala, Y., Bayona, J. A., Karaeminogullari, A., Perdomo-Ortiz, J. & Ramos-Mejia, M. We Are Very Similar but Not Really: The Moderating Role of Cultural Identification for Refugee Resettlement of Venezuelans in Colombia. *Frontiers in Psychology* **11**, 569394 (2020).

64. Aycan, Z. & Berry, J. W. Impact of employment-related experiences on immigrants' psychological well-being and adaptation to Canada. *Canadian Journal of Behavioural Science / Revue canadienne des sciences du comportement* **28**, 240–251 (1996).

65. Baba, Y. & Hosoda, M. Home away home: Better understanding of the role of social support in predicting

cross-cultural adjustment among international students. *College Student Journal* (2014).

66. Bader, B., Berg, N. & Holtbrügge, D. Expatriate performance in terrorism-endangered countries: The role of family and organizational support. *International Business Review* **24**, 849-860 (2015).

67. Bader, B. & Schuster, T. Expatriate Social Networks in Terrorism-Endangered Countries: An Empirical Analysis in Afghanistan, India, Pakistan, and Saudi Arabia. *Journal of International Management* **21**, 63-77 (2015).

68. Bagci, S. C. & Canpolat, E. Group efficacy as a moderator on the associations between perceived discrimination, acculturation orientations, and psychological well-being. *Journal of Community & Applied Social Psychology* **30**, 45-58 (2019).

69. Bai, J. Development and validation of the Acculturative Stress Scale for Chinese College Students in the United States (ASSCS). *Psychological Assessment* **28**, 443-447 (2016).

70. Bailey, C. A., *et al.* Risk and protective markers for well-being in Latinx immigrants in removal proceedings. *Law and Human Behavior* **45**, 179-196 (2021).

71. Bak-Klimek, A. The level and determinants of well-being among polish economic migrants in scotland: testing the sustainable happiness model: a sequential explanatory mixed-methods study. (Edinburgh Napier University, 2015).

72. Baker, C. J. Long-Term Depressive Symptoms and Acculturative Stress Issues Among Immigrants From the Former Soviet Union. *Journal of the American Psychiatric Nurses Association* **22**, 368-377 (2016).

73. Bakker, W., van Oudenhoven, J. P. & van der Zee, K. I. Attachment styles, personality, and Dutch emigrants' intercultural adjustment. *European Journal of Personality* **18**, 387-404 (2004).

74. Bao, J. Influences of Stress, Individual and Family Processes on Rural Low-Income Children's Internalizing and Externalizing Behaviors. (ProQuest Dissertations and Theses, 2019).

75. Baranik, L. E., Hurst, C. S. & Eby, L. T. The stigma of being a refugee: A mixed-method study of refugees' experiences of vocational stress. *Journal of Vocational Behavior* **105**, 116-130 (2018).

76. Barry, D. T. & Grilo, C. M. Cultural, self-esteem, and demographic correlates of perception of personal and group discrimination among East Asian immigrants. *Am J Orthopsychiatry* **73**, 223-229 (2003).

77. Basow, S. A. & Gaugler, T. Predicting adjustment of U.S. college students studying abroad: Beyond the multicultural personality. *International Journal of Intercultural Relations* **56**, 39-51 (2017).

78. Bastien, G., Seifen-Adkins, T. & Johnson, L. R. Striving for success: Academic adjustment of international students in the U.S. *Journal of International Students* **8.0**, 1198-1219 (2018).

79. Becerra, D.*, et al.* Immigration policies and mental health: examining the relationship between immigration enforcement and depression, anxiety, and stress among Latino immigrants. *Journal of Ethnic & Cultural Diversity in Social Work* **29**, 43-59 (2020).

80. Beiser, M., Goodwill, A. M., Albanese, P., McShane, K. & Nowakowski, M. Predictors of immigrant children's mental health in Canada: selection, settlement contingencies, culture, or all of the above? *Social Psychiatry and Psychiatric Epidemiology* **49**, 743-756 (2014).

81. Beiser, M. & Hou, F. Predictors of positive mental health among refugees: Results from Canada's General Social Survey. *Transcultural psychiatry* **54**, 675-695 (2017).

82. Bektaş, D. Y. Psychological adaptation and acculturation of the Turkish students in the United States. (2004).

83. Bektaş, Y., Demir, A. & Bowden, R. Psychological Adaptation of Turkish Students at U.S. Campuses. *International Journal for the Advancement of Counselling* **31**, 130-143 (2009).

84. Bekteshi, V. & van Hook, M. Contextual Approach to Acculturative Stress Among Latina Immigrants in the U.S. *Journal of Immigrant and Minority Health* **17**, 1401-1411 (2015).

85. Bekteshi, V., Van Hook, M. & Matthew, L. Puerto Rican-Born Women in the United States: Contextual Approach to Immigration Challenges. *Health & Social Work* **40**, 298-306 (2015).

86. Belau, M. H., Becher, H. & Kraemer, A. Impact of Family Separation on Subjective Time Pressure and Mental Health in Refugees from the Middle East and Africa Resettled in North Rhine-Westphalia, Germany: A Cross-Sectional Study. *International journal of environmental research and public health* **18**(2021).

87. Benita, M. S. Are the student migrants satisfied with life Effect of acculturative stress and perceived discrimination. *International Journal of Education Economics and Development* **12**, 79-96 (2021).

88. Bentley, J. A. & Dolezal, M. L. Does Time in Migration Exacerbate Posttraumatic Symptoms Among Internationally Displaced East African Refugees? *Journal of Immigrant & Refugee Studies* **17**, 371-388 (2018).

89. Bentley, J. A., Dolezal, M. L. & Alsubaie, M. K. Does Duration of Residency in the United States Influence Psychological Symptoms and Postmigration Stressors Among Refugees? Potential Implications of Populism for Refugee Mental Health. *International Perspectives in Psychology* **8**, 161-176 (2019).

90. Benyamini, Y., Boyko, V., Blumstein, T. & Lerner-Geva, L. Health, cultural and socioeconomic factors related to self-rated health of long-term Jewish residents, immigrants, and Arab women in midlife in Israel. *Women Health* **54**, 402-424 (2014).

91. Berger, R., Safdar, S., Spiess, E., Bekk, M. & Font, A. Acculturation of Erasmus students: Using the multidimensional individual difference acculturation model framework. *International Journal of Psychology* **54**, 739-749 (2019).

92. Berrios-Riquelme, J., Maluenda-Albornoz, J. & Castillo-Rozas, G. Perceived discrimination and mental health of South American immigrants in Chile: the mediator role of the self-esteem in four nationalities. *Social Work in Mental Health* **20**, 282-298 (2022).

93. Berthold, S. M. War traumas and community violence: Psychologieal, behavioral, and academic outcomes among Khmer refugee adolescents. in *Journal of Multicultural Social Work* 15-46 (2000).

94. Bethel, A., Ward, C. & Fetvadjiev, V. H. Cross-Cultural Transition and Psychological Adaptation of International Students: The Mediating Role of Host National Connectedness. *Frontiers in Education* **5**(2020).

95. Bhuian, S. N. & Al-Jabri, I. M. Expatriate turnover tendencies in Saudi Arabia: An empirical examination. *The International Journal of Organizational Analysis* **4**, 393-407 (1996).

96. Bierwiaczonek, K., Waldzus, S. & van der Zee, K. Protective or harmful? Exploring the ambivalent role of social identification as a moderator of intergroup stress in sojourners. *International Journal of Intercultural Relations* **60**, 1-11 (2017).

97. Bigler, M. *Exploratory study of distress among spouses of international students*, (ProQuest, 2007).

98. Billedo, C. J., Kerkhof, P. & Finkenauer, C. More facebook, less homesick? Investigating the short-term and long-term reciprocal relations of interactions, homesickness, and adjustment among international students. *International Journal of Intercultural Relations* **75**, 118-131 (2020).

99. Birman, D., Simon, C. D., Chan, W. Y. & Tran, N. A life domains perspective on acculturation and psychological adjustment: a study of refugees from the former Soviet Union. *American Journal of Community Psychology* **53**, 60-72 (2014).

100. Bissram, J. The effects of acculturative factors and academic self-efficacy on international students' psychological adjustment. (University at Albany, 2015).

101. Black, J. S. Work Role Transitions: A Study of American Expatriate Managers in Japan. *Journal of International Business Studies* **19**, 277-294 (1988).

102. Black, J. S. Locus of control, social support, stress, and adjustment in international transfers. *Asia Pacific Journal of Management* **7**, 1-29 (1990).

103. Black, J. S. & Gregersen, H. B. Antecedents to Cross-Cultural Adjusment for Expatriates in Pacific Rim Assignments. *Human Relations* **44**, 497-515 (1991).

104. Black, J. S. & Stephens, G. K. The Influence of the Spouse on American Expatriate Adjustment and Intent to Stay in Pacific Rim Overseas Assignments. *Journal of Management* **15**, 529-544 (1989).

105. Blackwell, M. A., Lardier, D. T., Choe, R. & Goodkind, J. R. Geocultural variation in correlates of psychological distress among refugees resettled in the United States. *Journal of Traumatic Stress* **35**, 1472-1483 (2022).

106. Bobowik, M., Basabe, N. & Paez, D. The bright side of migration: hedonic, psychological, and social well-being in immigrants in Spain. *Social science research* **51**, 189-204 (2015).

107. Boge, K., Karnouk, C., Hahn, E., Demir, Z. & Bajbouj, M. On Perceived Stress and Social Support: Depressive, Anxiety and Trauma-Related Symptoms in Arabic-Speaking Refugees in Jordan and Germany. *Frontiers in Public Health* **8**, 239 (2020).

108. Boiger, M. Adaptation of International Students in Japan: The cultural fit of control orientation. (2008).

109. Borho, A., Morawa, E., Schmitt, G. M. & Erim, Y. Somatic distress among Syrian refugees with residence

permission in Germany: analysis of a cross-sectional register-based study. *BMC Public Health* **21**, 896 (2021).

110. Borho, A., *et al.* The prevalence and risk factors for mental distress among Syrian refugees in Germany: a register-based follow-up study. *BMC Psychiatry* **20**, 362 (2020).

111. Bozionelos, N. Expatriation Outside the Boundaries of the Multinational Corporation: A Study with Expatriate Nurses in Saudi Arabia. *Human Resource Management* **48**, 111-134 (2009).

112. Brailovskaia, J., Schönfeld, P., Kochetkov, Y. & Margraf, J. What Does Migration Mean to Us? USA and Russia: Relationship Between Migration, Resilience, Social Support, Happiness, Life Satisfaction, Depression, Anxiety and Stress. *Current Psychology* **38**, 421-431 (2017).

113. Bridekirk, J., Hynie, M. & SyRia.lth. The Impact of Education and Employment Quality on Self-rated Mental Health Among Syrian Refugees in Canada. *Journal of Immigrant and Minority Health* **23**, 290-297 (2021).

114. Briones, E., Verkuyten, M., Cosano, J. & Tabernero, C. Psychological adaptation of Moroccan and Ecuadorean immigrant adolescents in Spain. *International Journal of Psychology* **47**, 28-38 (2012).

115. Brisset, C., Safdar, S., Lewis, J. R. & Sabatier, C. Psychological and sociocultural adaptation of university students in France: The case of Vietnamese international students. *International Journal of Intercultural Relations* **34**, 413-426 (2010).

116. Brown, R., *et al.* Acculturation attitudes and social adjustment in British South Asian children: A longitudinal study. *Personality and Social Psychology Bulletin* **39**, 1656-1667 (2013).

117. Browne, D. T., *et al.* Emotional problems among recent immigrants and parenting status: Findings from a national longitudinal study of immigrants in Canada. *PLoS One* **12**, e0175023 (2017).

118. Browne, S. T. Black Caribbeans' Post-migration Wellbeing: Are Religiosity and Social Support Enough to Protect against Acculturative Stress? (ProQuest Dissertations and Theses, 2018).

119. Bruning, N. S., Sonpar, K. & Wang, X. Host-country national networks and expatriate effectiveness: A mixed-methods study. *Journal of International Business Studies* **43**, 444-450 (2012).

120. Brunnet, A. E., Bolasell, L. T., Weber, J. & Kristensen, C. H. Prevalence and factors associated with

PTSD, anxiety and depression symptoms in Haitian migrants in southern Brazil. *International Journal of Social Psychiatry* **64**, 17-25 (2018).

121. Brunsting, N. C.*, et al.* Sources of Perceived Social Support, Social-Emotional Experiences, and Psychological Well-Being of International Students. *The Journal of Experimental Education* **89**, 95-111 (2019).

122. Buber-Ennser, I., Kohlenberger, J., Landesmann, M., Leitner, S. & Rengs, B. The Effect of Stressors and Resilience Factors on Mental Health of Recent Refugees in Austria. *IDEAS Working Paper Series from RePEc* (2019).

123. Buchanan, Z. E., Abu-Rayya, H. M., Kashima, E., Paxton, S. J. & Sam, D. L. Perceived discrimination, language proficiencies, and adaptation: Comparisons between refugee and non-refugee immigrant youth in Australia. *International Journal of Intercultural Relations* **63**, 105-112 (2018).

124. Buckingham, S. L. & Suarez-Pedraza, M. C. "It has cost me a lot to adapt to here": The divergence of real acculturation from ideal acculturation impacts Latinx immigrants' psychosocial wellbeing. *American Journal of Orthopsychiatry* **89**, 406-419 (2019).

125. Budak, F., Özer, Ö. & Özkan, O. Investigation of COVID-19 Risk Perception and Psychological Well-being in Syrian Refugees. *Smith College Studies in Social Work* **91**, 309-323 (2021).

126. Bulgan, G. & Çiftçi, A. Work-family balance and psychosocial adjustment of married international students. *Journal of International Students* **8.0**, 1079-1107 (2018).

127. Bulut, E. & Gayman, M. D. A latent class analysis of acculturation and depressive symptoms among Latino immigrants: Examining the role of social support. *International Journal of Intercultural Relations* **76**, 13-25 (2020).

128. Caligiuri, P. M. Selecting expatriates for personality characteristics: A moderating effect of personality on the relationship between host national contact and cross-cultural adjustment. in *International Human Resource Management: From Cross-Cultural Management to Managing a Diverse Workforce* 33-52 (2017).

129. Caligiuri, P. M., Joshi, A. & Lazarova, M. Factors influencing the adjustment of women on global

assignments. *The International Journal of Human Resource Management* **10**, 163-179 (1999).

130. Calvo, R. & Cheung, F. Does Money Buy Immigrant Happiness? *Journal of Happiness Studies* **19**, 1657-1672 (2017).

131. Camacho de Anda, A. & Becerra, D. Hoping for a Better Tomorrow: Do Hope and Optimism Serve as Protective Factors Against Discrimination in Latinx Immigrants. *Journal of Human Behavior in the Social Environment* **33**, 143-162 (2021).

132. Can, A. An Examination of the Relationship Between Adjustment Problems, Homesickness, Perceived Discrimination and Psychological Wellbeing among International Students (Ohio University, 2015).

133. Can, A., Poyrazl, S. & Pillay, Y. Eleven Types of Adjustment Problems and Psychological Well-Being among International Students. *Eurasian Journal of Educational Research* **91**, 1-20 (2021).

134. Cantekin, D. & Gençöz, T. Mental Health of Syrian Asylum Seekers in Turkey: The Role of Pre-Migration and Post-Migration Risk Factors. *Journal of Social and Clinical Psychology* **36**, 835-859 (2017).

135. Cantonis, A. M. Predictors of Parenting Stress in Hispanic Immigrant Mothers in New York City: The Roles of Risk Factors, Social Support, and Length of Time Living in the United States. (Florida State University, 2016).

136. Cao, C. & Meng, Q. Mapping the Paths from Language Proficiency to Adaptation for Chinese Students in a Non-English Speaking Country: an Integrative Model of Mediation. *Current Psychology* **38**, 1564-1575 (2019).

137. Cao, C., Zhang, J. & Meng, Q. A social cognitive model predicting international students' cross-cultural adjustment in China. *Current Psychology* **42**, 14529-14541 (2022).

138. Cao, C., Zhu, D. C. & Meng, Q. An exploratory study of inter-relationships of acculturative stressors among Chinese students from six European union (EU) countries. *International Journal of Intercultural Relations* **55**, 8-19 (2016).

139. Caplan, S. & Buyske, S. Depression, Help-Seeking and Self-Recognition of Depression among Dominican, Ecuadorian and Colombian Immigrant Primary Care Patients in the Northeastern United States. *International journal of environmental research and public health* **12**, 10450-10474 (2015).

140. Carella, M., García-Pereiro, T. & Pace, R. Subjective Well-Being, Transnational Families and Social Integration of Married Immigrants in Italy. *Social Indicators Research* **161**, 785-816 (2020).

141. Cariello, A. N., Perrin, P. B. & Morlett-Paredes, A. Influence of resilience on the relations among acculturative stress, somatization, and anxiety in latinx immigrants. *Brain and behavior* **10**, e01863 (2020).

142. Cariello, A. N.*, et al.* Moderating influence of enculturation on the relations between minority stressors and physical health via anxiety in Latinx immigrants. *Cultural Diversity and Ethnic Minority Psychology* **26**, 356-366 (2020).

143. Carrero Pinedo, A. Latinx Immigrant Health and Well-Being in the United States: Examining Legal Status as a Social Determinant of Health. (ProQuest Dissertations and Theses, 2021).

144. Casado, B. L. & Leung, P. Migratory Grief and Depression Among Elderly Chinese American Immigrants. in *Social Work Practice with the Asian American Elderly* 5-26 (2018).

145. Castellanos, R. Acculturative Stress and Depression Among Latinos|as: Investigating the Role of Neighborhood Context. (ProQuest Dissertations and Theses, 2017).

146. Cemalcilar, Z., Falbo, T. & Stapleton, L. M. Cyber communication: A new opportunity for international students' adaptation? *International Journal of Intercultural Relations* **29**, 91-110 (2005).

147. Cerezo, A. The impact of discrimination on mental health symptomatology in sexual minority immigrant Latinas. *Psychology of Sexual Orientation and Gender Diversity* **3**, 283-292 (2016).

148. Cetinkaya-Yildiz, E., Cakir, S. G. & Kondakci, Y. Psychological distress among international students in Turkey. *International Journal of Intercultural Relations* **35**, 534-539 (2011).

149. Chadwick, K. A. & Collins, P. A. Examining the relationship between social support availability, urban center size, and self-perceived mental health of recent immigrants to Canada: a mixed-methods analysis. *Social Science & Medicine* **128**, 220-230 (2015).

150. Chae, S. M., Park, J. W. & Kang, H. S. Relationships of acculturative stress, depression, and social support to health-related quality of life in Vietnamese immigrant women in South Korea. *Journal of Transcultural Nursing* **25**, 137-144 (2014).

151. Chai, D. S. The impact of positive psychological capital and perceived support on work performance of Korean expatriates: The mediating effects of cross-cultural adjustment and work engagement. (Texas A & M University, 2016).

152. Chai, D. S., Van, H. T. M., Wang, C.-W., Lee, J. & Wang, J. What Do International Students Need? The Role of Family and Community Supports for Adjustment, Engagement, and Organizational Citizenship Behavior. *Journal of International Students* **10**, 571-589 (2020).

153. Chamberlain-Gordon, L. E. Psychological Relationship between Acculturation and Caribbean Immigrants in the Northeast United States. (ProQuest Dissertations and Theses, 2019).

154. Chan, H. L., Toh, P. S., Ng, S. I. & Zawawi, D. I love my career, don't I? The influence of organisation-based support on expatriates' adjustment and occupational withdrawal intention. *International Journal of Business Science and Applied Management* **16.0**, 1-17 (2021).

155. Chan, H. L., Yaakob, A. R. & Pinjaman, S. Effect of Support from Lecturers and Host Country Nationals on Cross-Cultural Adjustment among International Students: Evidence from a Malaysian University. *Pertanika Journal of Social Sciences and Humanities* **29**, 1213-1229 (2021).

156. Chan, H. L., Zawawi, D. & Ng, S. I. Effects of primary stakeholders' support on expatriate adjustment and performance in Malaysia. *Jurnal Pengurusan* **56.0**(2019).

157. Chan, H. L., Zawawi, D., Ng, S. I. & Nipo, D. T. A. No expatriate is an island: the role of salient stakeholders' support in international assignments. *International Journal of Manpower* **43**, 1001-1018 (2021).

158. Chang, E., Chin, H. & Kwon, J. Inclusive, supportive, and fair workplaces for all: workplace satisfaction of low-skilled migrant workers. *The International Journal of Human Resource Management* **34**, 2202-2234 (2022).

159. Chang, L. C., Dattilo, J. & Huang, F. H. Relationships of Leisure Social Support and Flow with Loneliness in International Students in Taiwan: Implications during the COVID-19 Pandemic. *Leisure Sciences*, 1-18 (2022).

160. Chang, M. Cross-cultural comparative study of psychological distress between older Korean immigrants

in the United States and older Koreans in South Korea. *Aging & Mental Health* **23**, 1234-1245 (2019).

161. Chang, M. & Moon, A. Correlates and Predictors of Psychological Distress Among Older Asian Immigrants in California. *Journal of gerontological social work* **59**, 77-97 (2016).

162. Chang, W. Predictors of international students' socio-cultural adjustment. (ProQuest Dissertations and Theses, 2016).

163. Chapdelaine, R. F. & Alexitch, L. Social Skills Difficulty: Model of Culture Shock for International Graduate Students. *Journal of College Student Development* **45**, 167-184 (2004).

164. Chavanovanich, J. The associations between acculturation orientations and attitudinal outcomes among immigrant employees and international students. (Brunel University London, 2016).

165. Chayinska, M. & Mari, S. Paying attention to international students in Italy: The role of acculturative stress in the affective evaluations of cross-cultural transition. *Psicologia Sociale* **9.0**, 177-201 (2014).

166. Cheah, C. S.*, et al.* Parenting hassles mediate predictors of Chinese and Korean immigrants' psychologically controlling parenting. *Journal of Applied Developmental Psychology* **47**, 13-22 (2016).

167. Chen, A. S. CQ at work and the impact of intercultural training: An empirical test among foreign laborers. *International Journal of Intercultural Relations* **47**, 101-112 (2015).

168. Chen, A. S., Lin, G. & Yang, H. Staying connected: Effects of social connectedness, cultural intelligence, and socioeconomic status on overseas students' life satisfaction. *International Journal of Intercultural Relations* **83**, 151-162 (2021).

169. Chen, A. S., Wu, I. & Bian, M. The moderating effects of active and agreeable conflict management styles on cultural intelligence and cross-cultural adjustment. *International Journal of Cross Cultural Management* **14**, 270-288 (2014).

170. Chen, G., Kirkman, B. L., Kim, K., Farh, C. I. C. & Tangirala, S. When Does Cross-Cultural Motivation Enhance Expatriate Effectiveness? A Multilevel Investigation of the Moderating Roles of Subsidiary Support and Cultural Distance. *Academy of Management Journal* **53**, 1110-1130 (2010).

171. Chen, H.-F. The relationships of organizational justice, social exchange, psychological contract, and expatriate adjustment: an example of Taiwanese business expatriates. *The International Journal of Human*

Resource Management **21**, 1090-1107 (2010).

172. Chen, H.-J., Mallinckrodt, B. & Mobley, M. Attachment Patterns of East Asian International Students and Sources of Perceived Social Support as Moderators of the Impact of U.S. Racism and Cultural Distress. *Asian Journal of Counselling* **9**, 27–48 (2002).

173. Chen, I. H., Niu, S. F., Yeh, Y. C., Chen, I. J. & Kuo, S. F. Psychological distress among immigrant women who divorced: Resilience as a mediator. *Archives of Psychiatric Nursing* **39**, 1-6 (2022).

174. Chen, J., Li, Z., Xu, D. & Wu, X. Effects of Neighborhood Discrimination Towards Mainland Immigrants on Mental Health in Hong Kong. *International journal of environmental research and public health* **16**(2019).

175. Chen, K.-H., Yien, J.-M., Huang, C.-J. & Huang, K.-P. Social support, overseas adjustments and work performance of foreign labors in Taiwan. *African Journal of Business Management* (2011).

176. Chen, S. H., Zhang, E., Liu, C. H. & Wang, L. K. Depressive symptoms in Chinese immigrant mothers: Relations with perceptions of social status and interpersonal support. *Cultural Diversity and Ethnic Minority Psychology* **27**, 72-81 (2021).

177. Chen, S. X., Benet-Martínez, V. & Harris Bond, M. Bicultural Identity, Bilingualism, and Psychological Adjustment in Multicultural Societies: Immigration-Based and Globalization-Based Acculturation. *Journal of Personality* **76**, 803-838 (2008).

178. Chen, W., Hall, B. J., Ling, L. & Renzaho, A. M. Pre-migration and post-migration factors associated with mental health in humanitarian migrants in Australia and the moderation effect of post-migration stressors: findings from the first wave data of the BNLA cohort study. *Lancet Psychiatry* **4**, 218-229 (2017).

179. Chen, W., Ling, L. & Renzaho, A. M. Building a new life in Australia: an analysis of the first wave of the longitudinal study of humanitarian migrants in Australia to assess the association between social integration and self-rated health. *BMJ Open* **7**, e014313 (2017).

180. Chen, Y.-P. A three-stage process model of self-initiated expatriate career transitions: A self-determination theory perspective. (ProQuest Dissertations and Theses, 2012).

181. Chen, Y.-P. & Shaffer, M. The influence of expatriate spouses' coping strategies on expatriate and spouse adjustment. *Journal of Global Mobility: The Home of Expatriate Management Research* **6**, 20-39 (2018).

182. Cheng, Y., Meng, J. & Liu, S. Personal Network Structure and Perceived Social Support in the Context of Intercultural Adjustment. *Communication Quarterly* **66**, 576-594 (2018).

183. Chirkov, V. I., Safdar, S., de Guzman, D. J. & Playford, K. Further examining the role motivation to study abroad plays in the adaptation of international students in Canada. *International Journal of Intercultural Relations* **32**, 427-440 (2008).

184. Chiu, Y.-P., Wu, M., Zhuang, W.-L. & Hsu, Y.-Y. Influences on expatriate social networks in China. *The International Journal of Human Resource Management* **20**, 790-809 (2009).

185. Cho, H. J., Levesque-Bristol, C. & Yough, M. How autonomy-supportive learning environments promote Asian international students' academic adjustment: a self-determination theory perspective. *Learning Environments Research* **26**, 51-76 (2022).

186. Cho, J. & Yu, H. Roles of University Support for International Students in the United States. *Journal of Studies in International Education* **19**, 11-27 (2014).

187. Cho, S.*, et al.* Health-related quality of life among migrant workers: The impact of health-promoting behaviors. *Nurs Health Sci* **22**, 318-327 (2020).

188. Cho, Y. J., Jang, Y., Ko, J. E., Lee, S. H. & Moon, S. K. Acculturation, Acculturative Stress, and Depressive Symptoms in International Migrants: A Study with Vietnamese Women in South Korea. *Journal of Immigrant and Minority Health* **20**, 1103-1108 (2018).

189. Cho, Y. J., Jang, Y., Ko, J. E., Lee, S. H. & Moon, S. K. Perceived discrimination and depressive symptoms: a study of Vietnamese women who migrated to South Korea due to marriage. *Women Health* **60**, 863-871 (2020).

190. Choi, J. & Chung, W. Communicating in a different culture: Identifying acculturative stress among international students in South Korea. *Communication and Medicine* **11**, 249-261 (2016).

191. Choi, S., Lee, S., Kim, J. H. & Na, J. Effects of community integration on quality of life among Asian Americans. *International social work* **62**, 1404-1415 (2019).

192. Choi, Y.-J. & Park, G.-H. Associations among acculturation stress, mental health literacy, and mental health of married immigrant women in Korea. *International Journal of Mental Health Promotion* **18**, 234-246 (2016).

193. Chou, K.-L. Perceived discrimination and depression among new migrants to Hong Kong: The moderating role of social support and neighborhood collective efficacy. *Journal of Affective Disorders* **138**, 63-70 (2012).

194. Chu, H. & Lu, H. Acculturation, Bilateral Hostility, and Psychological Wellbeing of U.S.-dwelling Chinese during the COVID-19 Pandemic. *Health Communication* **38**, 1281-1292 (2023).

195. Chu, J. J., Khan, M. H., Jahn, H. J. & Kraemer, A. Only-Child Status in Relation to Perceived Stress and Studying-Related Life Satisfaction among University Students in China: A Comparison with International Students. *PLoS One* **10**, e0144947 (2015).

196. Chu, Y., Shen, C. & Yang, J. Country-level bonding, bridging, and linking social capital and immigrants' life satisfaction. *Applied Research in Quality of Life* **13**, 745-759 (2018).

197. Chung, G. H. & Lim, J. Y. Marriage Immigrant Mothers' Experience of Perceived Discrimination, Maternal Depression, Parenting Behaviors, and Adolescent Psychological Adjustment Among Multicultural Families in South Korea. *Journal of Child and Family Studies* **25**, 2894-2903 (2016).

198. Chung, H. & Epstein, N. B. Perceived racial discrimination, acculturative stress, and psychological distress among Asian immigrants: The moderating effects of support and interpersonal strain from a partner. *International Journal of Intercultural Relations* **42**, 129-139 (2014).

199. Chung, R. Y.-N. & Mak, J. K.-L. Physical and Mental Health of Live-In Female Migrant Domestic Workers: A Randomly Sampled Survey in Hong Kong. *American Behavioral Scientist* **64**, 802-822 (2020).

200. Claus, L., Maletz, S., Casoinic, D. & Pierson, K. Social capital and cultural adjustment of international assignees in NGOs: do support networks really matter? *The International Journal of Human Resource Management* **26**, 2523-2542 (2015).

201. Cloos, P., *et al.* The negative self-perceived health of migrants with precarious status in Montreal, Canada:

A cross-sectional study. *PLoS One* **15**, e0231327 (2020).

202. Cobb, C. L.*, et al.* Perceived discrimination and well-being among unauthorized Hispanic immigrants: The moderating role of ethnic/racial group identity centrality. *Cultural Diversity and Ethnic Minority Psychology* **25**, 280-287 (2019).

203. Cobb, C. L., Meca, A., Xie, D., Schwartz, S. J. & Moise, R. K. Perceptions of legal status: Associations with psychosocial experiences among undocumented Latino/a immigrants. *Journal of Counseling Psychology* **64**, 167-178 (2017).

204. Cole, N. D. Managing global talent: solving the spousal adjustment problem. *The International Journal of Human Resource Management* **22**, 1504-1530 (2011).

205. Cong, C. *International Students' Satisfaction with Educational Service Augmenters and Their Adjustment to the US Higher Education Institutions*, (Old Dominion University, 2017).

206. Conghui, S. A comparative study of intercultural adjustment of international students from the perspective of cultural distance. (Shanghai International Studies University, 2012).

207. Constantine, M. G., Okazaki, S. & Utsey, S. O. Self-Concealment, Social Self-Efficacy, Acculturative Stress, and Depression in African, Asian, and Latin American International College Students. *American Journal of Orthopsychiatry* **74**, 230-241 (2004).

208. Copeland, A. P. & Norell, S. K. Spousal adjustment on international assignments: the role of social support. *International Journal of Intercultural Relations* **26**, 255-272 (2002).

209. Cordeu Cuccia, C. Child and parental acculturation attitudes and child well-being: concurrent and longitudinal relationships among children in immigrant contexts. (University of Sussex, 2016).

210. Correa-Velez, I.*, et al.* Social Context Matters: Predictors of Quality of Life among Recently Arrived Refugee Women-at-Risk Living in Australia. *Journal of Immigrant & Refugee Studies* **18**, 498-514 (2020).

211. Counted, V. Attachment and quality of life in Australian religious African diasporas: A mixed methods study. (ProQuest Dissertations and Theses, 2019).

212. Cross, S. E. Self-Construals, Coping, and Stress in Cross-Cultural Adaptation. *Journal of Cross-Cultural*

Psychology **26**, 673-697 (1995).

213. Cura, Ü. & Işık, A. N. Impact of Acculturative Stress and Social Support on Academic Adjustment of International Students. *Education & Science/Egitim ve Bilim* **41**(2016).

214. Da Silva, N., Dillon, F. R., Rose Verdejo, T., Sanchez, M. & De La Rosa, M. Acculturative Stress, Psychological Distress, and Religious Coping Among Latina Young Adult Immigrants. *The Counseling Psychologist* **45**, 213-236 (2017).

215. Dadouch, Z. Adult Syrian Refugees Resettled in the United States: Social Support, Personality, Somatic Complaints, and Posttraumatic Stress Disorder. (ProQuest Dissertations and Theses, 2019).

216. Dang, V. T. & Chou, Y.-C. Extrinsic motivation, workplace learning, employer trust, self-efficacy and cross-cultural adjustment. *Personnel Review* **49**, 1232-1253 (2019).

217. David, E. M., Volpone, S. D. & Nandialath, A. M. Fostering longevity attitudes in women expatriates: the role of general and targeted types of organizational support. *The International Journal of Human Resource Management* **32**, 3833-3861 (2019).

218. Davies, S. E., Stoermer, S. & Froese, F. J. When the going gets tough: the influence of expatriate resilience and perceived organizational inclusion climate on work adjustment and turnover intentions. *The International Journal of Human Resource Management* **30**, 1393-1417 (2019).

219. Davis, A. N*., et al.* The Longitudinal Associations Between Discrimination, Depressive Symptoms, and Prosocial Behaviors in U.S. Latino/a Recent Immigrant Adolescents. *Journal of youth and adolescence* **45**, 457-470 (2016).

220. Dawson, A. Z., Walker, R. J., Gregory, C. & Egede, L. E. Examination of the Association Between Latent Variables for Social Determinants of Health and Blood Pressure Control in Immigrants using Structural Equation Modeling. *Journal of the National Medical Association* **112**, 186-197 (2020).

221. De Cieri, H., Dowling, P. J. & Taylor, K. F. The psychological impact of expatriate relocation on partners. *The International Journal of Human Resource Management* **2**, 377-414 (1991).

222. De Paul, N. F. & Bikos, L. H. Perceived organizational support: A meaningful contributor to expatriate development professionals' psychological well-being. *International Journal of Intercultural Relations* **49**,

25-32 (2015).

223. Demes, K. A. & Geeraert, N. Measures Matter. *Journal of Cross-Cultural Psychology* **45**, 91-109 (2013).

224. Dentakos, S., Wintre, M., Chavoshi, S. & Wright, L. Acculturation Motivation in International Student Adjustment and Permanent Residency Intentions. *Emerging Adulthood* **5**, 27-41 (2016).

225. Di Napoli, A.*, et al.* Self-perceived workplace discrimination and mental health among immigrant workers in Italy: a cross-sectional study. *BMC Psychiatry* **21**, 85 (2021).

226. Dillon, F. R.*, et al.* A Social Ecological Study of Psychological Distress among Recently Immigrated, Latina Young Adults. *Journal of Latinx Psychology* **7**, 39-58 (2018).

227. Dimitrova, M., Chia, S. I., Shaffer, M. A. & Tay-Lee, C. Forgotten travelers: Adjustment and career implications of international business travel for expatriates. *Journal of International Management* **26**(2020).

228. Dito, B. B., Mazzucato, V. & Schans, D. The Effects of Transnational Parenting on the Subjective Health and Well-Being of Ghanaian Migrants in the Netherlands. *Population, Space and Place* **23**(2017).

229. Do, K. A. T. Into the gap: A mixed methods study of acculturation, stress, and refugee family functioning. (ProQuest Dissertations and Theses, 2016).

230. Dominguez-Fuentes, J. M. & Hombrados-Mendieta, M. I. Social support and happiness in immigrant women in Spain. *Psychological reports* **110**, 977-990 (2012).

231. du Plooy, D. R., Lyons, A. & Kashima, E. S. Links between well-being and communication with friends and family in one's country of origin amongst migrants in Australia. *International Journal of Migration, Health and Social Care* **16**, 429-442 (2020).

232. Du, Y. & Wei, M. Acculturation, Enculturation, Social Connectedness, and Subjective Well-Being Among Chinese International Students. *The Counseling Psychologist* **43**, 299-325 (2015).

233. Duru, E. & Poyrazli, S. Personality Dimensions, Psychosocial- Demographic Variables, and English Language Competency in Predicting Level of Acculturative Stress Among Turkish International Students. *International Journal of Stress Management* **14**, 99-110 (2007).

234. Duru, E. & Poyrazli, S. Perceived discrimination, social connectedness, and other predictors of adjustment

difficulties among Turkish international students. *International Journal of Psychology* **46**, 446-454 (2011).

235. East, P. L., Gahagan, S. & Al-Delaimy, W. K. The Impact of Refugee Mothers' Trauma, Posttraumatic Stress, and Depression on Their Children's Adjustment. *Journal of Immigrant and Minority Health* **20**, 271-282 (2018).

236. Ekmen, E.*, et al.* How Does the Social Support Affect Refugees' Life Satisfaction in Turkey? Stress as a Mediator, Social Aids and Coronavirus Anxiety as Moderators. *Sustainability* **13**(2021).

237. El Khoury, S. J. Factors that impact the sociocultural adjustment and well-being of Syrian refugees in Stuttgart – Germany. *British Journal of Guidance & Counselling* **47**, 65-80 (2018).

238. Elgorriaga, E., Ibabe, I. & Arnoso, A. Spanish migrants to European Union countries: predictors of psychological adjustment /Españoles que emigran a países de la Unión Europea: predictores de su ajuste psicológico. *Revista de Psicología Social* **31**, 317-351 (2016).

239. English, A. S. & Zhang, R. Coping with perceived discrimination: A longitudinal study of sojourners in China. *Current Psychology* **39**, 854-869 (2019).

240. English, A. S.*, et al.* Ethnic Stereotype Formation and Its Impact on Sojourner Adaptation: A Case of "Belt and Road" Chinese Migrant Workers in Montenegro. *International journal of environmental research and public health* **18**(2021).

241. Enriquez, L. E., Morales Hernandez, M. & Ro, A. Deconstructing Immigrant Illegality: A Mixed-Methods Investigation of Stress and Health Among Undocumented College Students. *Race and Social Problems* **10**, 193-208 (2018).

242. Ercan, S. Antecedents of expatriates' organizational citizenship behavior: expatriate adjustment and job attitudes as mediators and cultural similarity as the moderator. (Rice University, 2014).

243. Ergin, D. A. The effects of perceived discrimination, social support and ethnic identity on mental health of immigrant adolescents. *Scandinavian journal of child and adolescent psychiatry and psychology* **9**, 127-136 (2021).

244. Espeleta, H. C., Beasley, L., Bohora, S., Ridings, L. E. & Silovsky, J. F. Depression in Latina mothers:

Examining the roles of acculturation, enculturation, social support, and family resources. *Cultural Diversity and Ethnic Minority Psychology* **25**, 527-538 (2019).

245. Espinoza-Castro, B., Vasquez Rueda, L. E., Mendoza Lopez, R. V. & Radon, K. Working Below Skill Level as Risk Factor for Distress Among Latin American Migrants Living in Germany: A Cross-Sectional Study. *Journal of Immigrant and Minority Health* **21**, 1012-1018 (2019).

246. Espinoza-Castro, B., Weinmann, T., Mendoza Lopez, R. & Radon, K. Working Conditions as Risk Factors for Depressive Symptoms among Spanish-Speaking Au Pairs Living in Germany-Longitudinal Study. *International journal of environmental research and public health* **18**(2021).

247. Estrada, F., Cerezo, A. & Ramirez, A. An Examination of Posttraumatic Stress Disorder-Related Symptoms Among a Sample of Latinx Sexual- and Gender-Minority Immigrants. *Journal of Traumatic Stress* **34**, 967-976 (2021).

248. Ezeofor, I. A social cognitive approach to coping with acculturative stress in international students. (University of Maryland, 2016).

249. Faeth, P. C. Working in Dangerous Contexts : Advancing the Conceptual and Empirical Approach to Work in Hostile Environments. (University of Stirling, 2019).

250. Falavarjani, M. F., Yeh, C. J. & Brouwers, S. A. Exploring the Effects of Acculturative Stress and Social Support on the Acculturation-Depression Relationship in Two Countries of Similar Social Status. *Journal of International Migration and Integration* **21**, 509-528 (2019).

251. Fanfan, D., Rodriguez, C. S., Groer, M., Weaver, M. & Stacciarini, J. R. Stress and depression in the context of migration among Haitians in the United States. *Health & Social Care in the Community* **28**, 1795-1806 (2020).

252. Fang, C. Y., Handorf, E. A., Rao, A. D., Siu, P. T. & Tseng, M. Acculturative Stress and Depressive Symptoms Among Chinese Immigrants: the Role of Gender and Social Support. *Journal of Racial and Ethnic Health Disparities* **8**, 1130-1138 (2021).

253. Fang, K., Friedlander, M. & Pieterse, A. L. Contributions of acculturation, enculturation, discrimination, and personality traits to social anxiety among Chinese immigrants: A context-specific assessment.

Cultural Diversity and Ethnic Minority Psychology **22**, 58-68 (2016).

254. Faran, Y. & Vered, S.-N. Perceived Discrimination as a Moderator between Living Difficulties and Psychological Distress among Asylum Seekers from Darfur. *Journal of Refugee Studies* **35**, 36-50 (2022).

255. Farcas, D. & Gonçalves, M. What is the effect of stressors and resources on the;expatriates' perception of the bidirectional work-;family conflict and cross-cultural adjustment? *The Second World Congress on Resilience:* (2014).

256. Fernández, I., Silván-Ferrero, P., Molero, F., Gaviria, E. & García-Ael, C. Perceived Discrimination and Well-Being in Romanian Immigrants: The Role of Social Support. *Journal of Happiness Studies* **16**, 857-870 (2014).

257. Fernandez-Esquer, M. E., Gallardo, K. R. & Diamond, P. M. Predicting the Influence of Situational and Immigration Stress on Latino Day Laborers' Workplace Injuries: An Exploratory Structural Equation Model. *Journal of Immigrant and Minority Health* **21**, 364-371 (2019).

258. Feyera, F., Mihretie, G., Bedaso, A., Gedle, D. & Kumera, G. Prevalence of depression and associated factors among Somali refugee at Melkadida camp, Southeast Ethiopia: a cross-sectional study. *BMC Psychiatry* **15**, 171 (2015).

259. Filipic Sterle, M., Vervoort, T. & Verhofstadt, L. L. Social Support, Adjustment, and Psychological Distress of Help-Seeking Expatriates. *Psychologica Belgica* **58**, 297-317 (2018).

260. Firth, B. M., Chen, G., Kirkman, B. L. & Kim, K. Newcomers Abroad: Expatriate Adaptation during Early Phases of International Assignments. *Academy of Management Journal* **57**, 280-300 (2014).

261. Fisher, U. M. The Moderating Effect of Family Functioning on the Well-Being of Adolescent Immigrants Who Experience Acculturation Distress. (University of Akron, 2017).

262. Florkowski, G. W. & Fogel, D. S. Expatriate adjustment and commitment: the role of host-unit treatment. *International Journal of Human Resource Management* **10**, 783-807 (1999).

263. Fogden, G., Berle, D. & Steel, Z. The Impact of Family Separation and Worry About Family on Psychological Adjustment in Refugees Resettled in Australia. *Journal of Traumatic Stress* **33**, 894-907 (2020).

264. Fontinha, R., De Cuyper, N., Williams, S. & Scott, P. The impact of HRM, perceived employability, and job insecurity on self-initiated expatriates' adjustment to the host country. *Thunderbird International Business Review* **60**, 861-871 (2018).

265. Forbush, E. & Foucault-Welles, B. Social media use and adaptation among Chinese students beginning to study in the United States. *International Journal of Intercultural Relations* **50**, 1-12 (2016).

266. Froese, F. J. & Peltokorpi, V. Organizational expatriates and self-initiated expatriates: differences in cross-cultural adjustment and job satisfaction. *The International Journal of Human Resource Management* **24**, 1953-1967 (2013).

267. Frost, D. M. Hostile and harmful: Structural stigma and minority stress explain increased anxiety among migrants living in the United Kingdom after the Brexit referendum. *Journal of Community Psychology* **88**, 75-81 (2020).

268. Fu, C., Hsu, Y.-S., Shaffer, M. A. & Ren, H. A longitudinal investigation of self-initiated expatriate organizational socialization. *Personnel Review* **46**, 182-204 (2017).

269. Furukawa, T., Sarason, I. G. & Sarason, B. R. Social Support and Adjustment to a Novel Social Environment. *International Journal of Social Psychiatry* **44**, 56-70 (1998).

270. Furukawa, T. & Shibayama, T. Predicting maladjustment of exchange students in different cultures: a prospective study. *Social Psychiatry and Psychiatric Epidemiology* **28**, 142-146 (1993).

271. Galchenko, I. & van de Vijver, F. J. R. The role of perceived cultural distance in the acculturation of exchange students in Russia. *International Journal of Intercultural Relations* **31**, 181-197 (2007).

272. Galvan, T., Rusch, D., Domenech Rodriguez, M. M. & Garcini, L. M. Familias Divididas [divided families]: Transnational family separation and undocumented Latinx immigrant health. *Journal of Family Psychology* **36**, 513-522 (2022).

273. Gao, G. & Gudykunst, W. B. Uncertainty, anxiety, and adaptation. *International Journal of Intercultural Relations* **14**, 301-317 (1990).

274. Garcia, E. The Role of Acculturative Stress in the Psychological Adjustment of Immigrant Hispanic Parents. (ProQuest Dissertations and Theses, 2016).

275. Garcia-Cid, A., Gomez-Jacinto, L., Hombrados-Mendieta, I., Millan-Franco, M. & Moscato, G. Discrimination and Psychosocial Well-Being of Migrants in Spain: The Moderating Role of Sense of Community. *Frontiers in Psychology* **11**, 2235 (2020).

276. Garcia-Cid, A., Hombrados-Mendieta, I., Gomez-Jacinto, L., Millan-Franco, M. & Del Pino-Brunet, N. The moderating effect of gender as a protective factor against discrimination in migrants from Latin America and China. *Journal of Community Psychology* **48**, 1964-1984 (2020).

277. Garcini, L. M.*, et al.* Kicks Hurt Less: Discrimination Predicts Distress Beyond Trauma among Undocumented Mexican Immigrants. *Psychology of violence* **8**, 692-701 (2018).

278. Garcini, L. M., Renzaho, A. M. N., Molina, M. & Ayala, G. X. Health-related quality of life among Mexican-origin Latinos: the role of immigration legal status. *Ethnicity & health* **23**, 566-581 (2018).

279. Gatina, L. Does money buy happiness? Financial and general well-being of immigrants in Australia. *Journal of Behavioral and Experimental Economics* **63**, 91-105 (2016).

280. Gebregergis, W. T., Huang, F. & Hong, J. The impact of emotional intelligence on depression among international students studying in China: The mediating effect of acculturative stress. *International Journal of Intercultural Relations* **79**, 82-93 (2020).

281. Genkova, A. G., Trickett, E. J., Birman, D. & Vinokurov, A. Acculturation and adjustment of elderly émigrés from the former Soviet Union: A life domains perspective. *Psychosocial Intervention* **23**, 83-93 (2014).

282. Georgiadou, E., Schmitt, G. M. & Erim, Y. Does the separation from marital partners of Syrian refugees with a residence permit in Germany have an impact on their quality of life? *Journal of psychosomatic research* **130**, 109936 (2020).

283. Gerega, M. The Impacts of Acculturation Styles and Social Support on Depression in Older Russian Immigrants. (ProQuest Dissertations and Theses, 2017).

284. Getnet, B., Medhin, G. & Alem, A. Symptoms of post-traumatic stress disorder and depression among Eritrean refugees in Ethiopia: identifying direct, meditating and moderating predictors from path analysis. *BMJ Open* **9**, e021142 (2019).

285. Ghaffari, A. & Çiftçi, A. Religiosity and Self-Esteem of Muslim Immigrants to the United States: The Moderating Role of Perceived Discrimination. *The International Journal for the Psychology of Religion* **20**, 14-25 (2010).

286. Gibbs, R., Güneri, O. Y., Pankau, T. & Bikos, L. Birds of a Feather Fare Less Well Together: Modeling Predictors of International Student Adaptation. *Sustainability* **12**(2020).

287. Giorgi, G*., et al.* The dark and the light side of the expatriate's cross-cultural adjustment: a novel framework including perceived organizational support, work related stress and innovation. *Sustainability* **12**, 2969 (2020).

288. Giuliani, C., Tagliabue, S. & Regalia, C. Psychological Well-Being, Multiple Identities, and Discrimination Among First and Second Generation Immigrant Muslims. *Europe's journal of psychology* **14**, 66-87 (2018).

289. Goede, J. Do they really want to leave? A (re)-evaluation of expatriates' and spouses' premature return intention. *Journal of Global Mobility: The Home of Expatriate Management Research* **8**, 209-228 (2020).

290. Gonçalves, M. & Matos, M. Mental health of multiple victimized immigrant women in Portugal: Does resilience make a difference? *Journal of Human Behavior in the Social Environment* **30**, 353-368 (2020).

291. Gong, S., Xu, P. & Wang, S. Social Capital and Psychological Well-Being of Chinese Immigrants in Japan. *International journal of environmental research and public health* **18**, 1-12 (2021).

292. Gong, Y. Goal orientations and cross-cultural adjustment: an exploratory study. *International Journal of Intercultural Relations* **27**, 297-305 (2003).

293. Gong, Y. & Fan, J. Longitudinal examination of the role of goal orientation in cross-cultural adjustment. *Journal of Applied Psychology* **91**, 176-184 (2006).

294. Gonzalez-Castro, J. L., Ubillos Landa, S., Puente Martinez, A. & Vera Perea, M. The Role of Emotional Intelligence and Sociocultural Adjustment on Migrants' Self-reported Mental Well-Being in Spain: A 14 Month Follow-Up Study. *International journal of environmental research and public health* **17**(2020).

295. Gopalan, N., Beutell, N. J. & Middlemiss, W. International students' academic satisfaction and turnover intentions. *Quality Assurance in Education* **27**, 533-548 (2019).

296. Gorodzeisky, A., Sarid, O., Mirsky, J. & Slonim-Nevo, V. Immigrant Families. *Journal of Cross-Cultural Psychology* **45**, 713-727 (2014).

297. Gottvall, M., Sjolund, S., Arwidson, C. & Saboonchi, F. Health-related quality of life among Syrian refugees resettled in Sweden. *Quality of Life Research* **29**, 505-514 (2020).

298. Grant-Vallone, E. J. & Ensher, E. A. An examination of work and personal life conflict, organizational support, and employee health among international expatriates. *International Journal of Intercultural Relations* **25**, 261-278 (2001).

299. Greene, R. N. Kinship, friendship, and service provider social ties and how they influence well-being among newly resettled refugees. *Socius* **5**(2019).

300. Gregersen, H. B. & Black, J. S. Antecedents to Commitment to a Parent Company and a Foreign Operation. *The Academy of Management Journal* **35**, 65-90 (1992).

301. Groen, S. P. N., Richters, A. J. M., Laban, C. J., van Busschbach, J. T. & Deville, W. Cultural Identity Confusion and Psychopathology: A Mixed-Methods Study Among Refugees and Asylum Seekers in the Netherlands. *The Journal of Nervous and Mental Disease* **207**, 162-170 (2019).

302. Gudmundsdottir, S., Gudlaugsson, T. O. & Adalsteinsson, G. D. The diplomatic spouse. *Journal of Global Mobility: The Home of Expatriate Management Research* **7**, 103-122 (2019).

303. Guerra, R., & Pires França, A. Adaptação de imigrantes Portugueses em Angola: estatuto, indispensabilidade funcional e estratégias de aculturação. (ISCTE-IUL, 2015).

304. Guerra, R*., et al.* School achievement and well-being of immigrant children: The role of acculturation orientations and perceived discrimination. *Journal of school psychology* **75**, 104-118 (2019).

305. Guillaume, M. The Influence of Person-environment Culture Value Fit on Cross-cultural Adaptation. (National Taiwan Normal University, 2019).

306. Gunasekara, A., Grant, S. & Rajendran, D. Years since migration and wellbeing among Indian and Sri Lankan skilled migrants in Australia: Mediating effects of acculturation. *International Journal of Intercultural Relations* **70**, 42-52 (2019).

307. Guo, Y., Li, Y. & Ito, N. Exploring the predicted effect of social networking site use on perceived social

capital and psychological well-being of Chinese international students in Japan. *Cyberpsychology, Behavior, and Social Networking* **17**, 52-58 (2014).

308. Ha, J. T. Mediating Effect of Acculturation Strategy on the Relationship between Acculturation Stress Factors and Global Psychological Distress: A Path Model. (ProQuest Dissertations and Theses, 2021).

309. Haagsman, K., Mazzucato, V. & Dito, B. B. Transnational families and the subjective well-being of migrant parents: Angolan and Nigerian parents in the Netherlands. *Ethnic and Racial Studies* **38**, 2652-2671 (2015).

310. Haase, A., Rohmann, A. & Hallmann, K. An ecological approach to psychological adjustment: A field survey among refugees in Germany. *International Journal of Intercultural Relations* **68**, 44-54 (2019).

311. Haer, R., Scharpf, F. & Hecker, T. The social legacies of conflict: The mediating role of mental health with regard to the association between war exposure and social capital of Burundian refugees. *Psychology of Violence* **11**, 40-49 (2021).

312. Hahm, S.-W. Effects of Social and Organizational Support on the Satisfaction of International Students and Career Commitment. *Turkish Journal of Computer and Mathematics Education* **12.0**, 3032-3038 (2021).

313. Hahn, E., Richter, D., Schupp, J. & Back, M. D. Predictors of Refugee Adjustment: The Importance of Cognitive Skills and Personality. *Collabra: Psychology* **5**(2019).

314. Haldorai, K., Kim, W. G., Seo, W. S. & Cai, X. Learning orientation and self-initiated expatriates' work performance: a moderated-mediation model. *International Journal of Hospitality Management* **94**(2021).

315. Halim, H., Abu Bakar, H. & Mohamad, B. Measuring multicultural effectiveness among self-initiated academic expatriates in Malaysia. *Jurnal Komunikasi Malaysian Journal of Communication* **34**, 1-17 (2018).

316. Halim, H., Bakar, H. A. & Mustaffa, C. S. The mediating roles of communication in expatriate adjustment. *Journal of telecommunication, electrnic and computer engineering* **8.0**, 129-132 (2016).

317. Halim, H., Mustaffa, C. S. & Azizan, F. L. Measuring work-role transitions: the cross-cultural experience of hotel expatriates in Malaysia. *Journal of media and communication research* **12.0**, 1-16 (2020).

318. Hall, B. J., *et al.* Exploring correlates of depression, quality of life and alcohol misuse: a nationwide cross-sectional study of international migrants during the COVID-19 epidemic in China. *BMJ Open* **11**, e048012 (2021).

319. Haro, A. Y., *et al.* Beyond Occupational Hazards: Abuse of Day Laborers and Health. *Journal of Immigrant and Minority Health* **22**, 1172-1183 (2020).

320. Hashemi, N., Marzban, M., Sebar, B. & Harris, N. Acculturation and psychological well-being among Middle Eastern migrants in Australia: The mediating role of social support and perceived discrimination. *International Journal of Intercultural Relations* **72**, 45-60 (2019).

321. Hashemi, N., Marzban, M., Sebar, B. & Harris, N. Religious identity and psychological well-being among middle-eastern migrants in Australia: The mediating role of perceived social support, social connectedness, and perceived discrimination. *Psychology of Religion and Spirituality* **12**, 475-486 (2020).

322. Haslam, C., *et al.* Ageing well in a foreign land: group memberships protect older immigrants' wellbeing through enabling social support and integration. *Ageing and Society* **42**, 1710-1732 (2020).

323. Hassan, Z., Jianxun, C., Qaisar, S., Shah, Z. & Ram, M. Exploring the effect of WeChat on adjustment of international students in China. *Cogent Psychology* **8**(2021).

324. Hechanova-Alampay, R., Beehr, T. A., Christiansen, N. D. & Van Horn, R. K. Adjustment and Strain among Domestic and International Student Sojourners: A Longitudinal Study. *School Psychology International* **23**, 458-474 (2002).

325. Helms, H. M., *et al.* Economic pressure, cultural adaptation stress, and marital quality among Mexican-origin couples. *Journal of Family Psychology* **28**, 77-87 (2014).

326. Hendrickson, B., Rosen, D. & Aune, R. K. An analysis of friendship networks, social connectedness, homesickness, and satisfaction levels of international students. *International Journal of Intercultural Relations* **35**, 281-295 (2011).

327. Herleman, H. A., Britt, T. W. & Hashima, P. Y. Ibasho and the adjustment, satisfaction, and well-being of expatriate spouses. *International Journal of Intercultural Relations* **32**, 282-299 (2008).

328. Herrero, J., Fuente, A. & Gracia, E. Covariates of subjective well-being among Latin American

immigrants in Spain: The role of social integration in the community. *Journal of Community Psychology* **39**, 761-775 (2011).

329. Hill, C. M., Williams, E. C. & Ornelas, I. J. Help Wanted: Mental Health and Social Stressors Among Latino Day Laborers. *American journal of men's health* **13**, 1557988319838424 (2019).

330. Hippler, T., Caligiuri, P. M., Johnson, J. E. & Baytalskaya, N. The development and validation of a theory-based expatriate adjustment scale. *The International Journal of Human Resource Management* **25**, 1938-1959 (2014).

331. Hirai, R., Frazier, P. & Syed, M. Psychological and sociocultural adjustment of first-year international students: Trajectories and predictors. *Journal of Counseling Psychology* **62**, 438-452 (2015).

332. Hofhuis, J., Hanke, K. & Rutten, T. Social network sites and acculturation of international sojourners in the Netherlands: The mediating role of psychological alienation and online social support. *International Journal of Intercultural Relations* **69**, 120-130 (2019).

333. Holliman, A.*, et al.* Examining the Relationship Between Adaptability, Social Support, and Psychological Wellbeing Among Chinese International Students at UK Universities. *Frontiers in Education* **7**(2022).

334. Holmes, L. M. & Marcelli, E. A. Neighborhood Social Cohesion and Serious Psychological Distress Among Brazilian Immigrants in Boston. *Community mental health journal* **56**, 149-156 (2020).

335. Hombrados-Mendieta, I.*, et al.* Positive influences of social support on sense of community, life satisfaction and the health of immigrants in Spain. *Frontiers in psychology* **10**, 2555 (2019).

336. Hosseini, A.*, et al.* Migration experience, resilience and depression: a study of Iranian immigrants living in Australia. *International Journal of Culture and Mental Health* **10**, 108-120 (2017).

337. Hovey, J. D. Acculturative stress, depression, and suicidal ideation in Mexican immigrants. *Cultural Diversity and Ethnic Minority Psychology* **6**, 134-151 (2000).

338. Htay, M. N. N., Latt, S. S., Maung, K. S., Myint, W. W. & Moe, S. Mental Well-Being and Its Associated Factors Among Myanmar Migrant Workers in Penang, Malaysia. *Asia Pacific Journal of Public Health* **32**, 320-327 (2020).

339. Hu, J. & Wang, Z. Exploring the associated factors of elevated psychological distress in a community

residing sample of Australian Chinese migrants. *Australian Journal of Psychology* **68**, 116-122 (2020).

340. Hu, S., Liu, H., Zhang, S. & Wang, G. Proactive personality and cross-cultural adjustment: Roles of social media usage and cultural intelligence. *International Journal of Intercultural Relations* **74**, 42-57 (2020).

341. Hu, Y. L., Roberts, A., Ching, G. S. & Chao, P. C. Moderating Effects of Intercultural Social Efficacy and the Role of Language in the Context of Coping Strategies in Study Abroad Depression. *International journal of environmental research and public health* **19**(2022).

342. Hua, J., *et al.* Examinations of the Role of Individual Adaptability in Cross-Cultural Adjustment. *Journal of Career Assessment* **27**, 490-509 (2018).

343. Hua, J., Zheng, L., Zhang, G. & Fan, J. Proactive personality and cross-cultural adjustment: A moderated mediation model. *International Journal of Intercultural Relations* **72**, 36-44 (2019).

344. Huang, H., Liu, H., Zhao, X., He, H. & Ding, Y. How simulated home influences Chinese expatriates: the partial mediating role of organizational embeddedness and organizational identification. *Employee Relations: The International Journal* **44**, 1290-1317 (2022).

345. Huang, S. L. & Mussap, A. J. Maladaptive Perfectionism, Acculturative Stress and Depression in Asian International University Students. *Journal of Psychologists and Counsellors in Schools* **28**, 185-196 (2016).

346. Huang, S. S. & Yang, H. J. Is There a Healthy Immigrant Effect Among Women Through Transnational Marriage? Results from Immigrant Women from Southeast Asian Countries in Taiwan. *Journal of Immigrant and Minority Health* **20**, 178-187 (2018).

347. Hudson-Gayle, D. M. College life in America: Perceived stress among English-speaking Caribbean students studying in American universities. (ProQuest Dissertations and Theses, 2015).

348. Huff, K. C., Song, P. & Gresch, E. B. Cultural intelligence, personality, and cross-cultural adjustment: A study of expatriates in Japan. *International Journal of Intercultural Relations* **38**, 151-157 (2014).

349. Huisman, J., Vlegels, J., Daenekindt, S., Seeber, M. & Laufer, M. How satisfied are international students? The role of town, gown and motivations. *Compare: A Journal of Comparative and International Education* **52**, 1332-1350 (2021).

350. Hung, C. C., Huan, T. C., Lee, C. H., Lin, H. M. & Zhuang, W. L. To adjust or not to adjust in the host country? Perspective of interactionism. *Employee Relations* **40**, 329-345 (2018).

351. Hussain, T. Employee turnover intentions of self-initiated expatriates in healthcare organisations in the United Arab Emirates. (King's College London, 2016).

352. Hussain, T. & Deery, S. Why do self-initiated expatriates quit their jobs: The role of job embeddedness and shocks in explaining turnover intentions. *International Business Review* **27**, 281-288 (2018).

353. Hwang, K. P., Wang, M.-K. & Sodanine, S. The Effects of Stressors, Living Support, and Adjustment on Learning Performance of International Students in Taiwan. *Social Behavior and Personality: An International Journal* **39**, 333-344 (2011).

354. Im, M. H., Choi, M. N. & Seo, J. B. A study on the mediating effect of acculturation on life satisfaction of marriage-based female immigrants. *Information (Japan)* **20.0**, 5857-5865 (2017).

355. Imai, T. & Imai, A. Cross-Ethnic Self-Disclosure Buffering Negative Impacts of Prejudice on International Students' Psychological and Social Well-Being. *Journal of International Students* **9**, 66-83 (2019).

356. Ismail, A. & Kahwa, K. M. Prevalence, Associated Factors, and Help Seeking Behavior Related to Psychological Distress among International Students at Universiti Kebangsaan Malaysia. *Malaysian Journal of Public Health Medicine* **20**, 215-223 (2020).

357. Jackson, M., Ray, S. & Bybell, D. International Students in the U.S.: Social and Psychological Adjustment. *Journal of International Students* **3**, 17-28 (2013).

358. Jakobsen, M., Meyer DeMott, M. A., Wentzel-Larsen, T. & Heir, T. The impact of the asylum process on mental health: a longitudinal study of unaccompanied refugee minors in Norway. *BMJ Open* **7**, e015157 (2017).

359. Jamaludin, N. L., Sam, D. L., Sandal, G. M. & Adam, A. A. The influence of perceived discrimination, orientation to mainstream culture and life satisfaction on destination loyalty intentions: the case of international students. *Current Issues in Tourism* **21**, 934-949 (2016).

360. James, P., Iyer, A. & Webb, T. L. The impact of post-migration stressors on refugees' emotional distress

and health: A longitudinal analysis. *European Journal of Social Psychology* **49**, 1359-1367 (2019).

361. Jamil, R. Mental Health and Arab Canadian Immigrants: Risks, Protective Factors, and Resilience. (ProQuest Dissertations and Theses, 2020).

362. Jamsiah, M. & Taher, S. Stress among international postgraduate students at universiti kebangsaan Malaysia medical centre (UKMMC). *Malaysian Journal of Public Health Medicine* **14.0**, 21-33 (2014).

363. Jang, H. & Tang, F. Loneliness, age at immigration, family relationships, and depression among older immigrants: A moderated relationship. *Journal of social and personal relationships* **39**, 1602-1622 (2022).

364. Jang, Y*., et al.* Perceived racial discrimination and mental distress in older Korean Americans: the moderating role of ethnic resources. *Ethnicity & health* **28**, 1-11 (2023).

365. Jang, Y*., et al.* Social capital in ethnic communities and mental health: a study of older Korean immigrants. *Journal of cross-cultural gerontology* **30**, 131-141 (2015).

366. Jankovic-Rankovic, J*., et al.* Transient refugees' social support, mental health, and physiological markers: Evidence from Serbian asylum centers. *American Journal of Human Biology* **34**, e23747 (2022).

367. Jannesari, M. & Sullivan, S. E. Career adaptability and the success of self-initiated expatriates in China. *Career Development International* **24**, 331-349 (2019).

368. Jannesari, M., Wang, Z., McCall, J. & Zheng, B. Psychological availability between self-initiated expatriates and host country nationals during their adjustment: the moderating role of supportive supervisor relations. *Frontiers in Psychology* **8**, 2049 (2017).

369. Jannesari, M. T. & Sullivan, S. E. How relationship quality, autonomous work motivation and socialization experience influence the adjustment of self-initiated expatriates in China. *Cross Cultural & Strategic Management* **28**, 309-331 (2021).

370. Jasinskaja-Lahti, I. & Liebkind, K. Perceived discrimination and psychological adjustment among Russian-speaking immigrant adolescents in Finland. *International Journal of Psychology* **36**, 174-185 (2001).

371. Jasinskaja-Lahti, I., Liebkind, K., Jaakkola, M. & Reuter, A. Perceived Discrimination, Social Support Networks, and Psychological Well-being Among Three Immigrant Groups. *Journal of Cross-Cultural*

Psychology **37**, 293-311 (2006).

372. Jasinskaja-Lahti, I., Liebkind, K. & Perhoniemi, R. Perceived discrimination and well-being: a victim study of different immigrant groups. *Journal of Community & Applied Social Psychology* **16**, 267-284 (2006).

373. Jeannite, S. Exploring the relationship between psychological symptoms and ethnic connectedness among the New York Metropolitan Area Haitian community to the 2010 earthquake in Haiti. (Pace University, 2015).

374. Jenkins, E. M. & Mockaitis, A. I. You're from where? The influence of distance factors on New Zealand expatriates' cross-cultural adjustment. *The International Journal of Human Resource Management* **21**, 2694-2715 (2010).

375. Jensen, T. K., Skar, A. S., Andersson, E. S. & Birkeland, M. S. Long-term mental health in unaccompanied refugee minors: pre- and post-flight predictors. *European child & adolescent psychiatry* **28**, 1671-1682 (2019).

376. Jeon, H. & Lubben, J. The Influence of Social Networks and Supports on Depression Symptoms: Differential Pathways for Older Korean Immigrants and Non-Hispanic White Americans. *Care Management Journals* **17**, 13-23 (2016).

377. Jhutty, S. P. Emotional intelligence and expatriate cross-cultural adjustment. (ProQuest Dissertations and Theses, 2007)

378. Ji, P. & Duan, C. The Relationship Among Acculturation, Acculturation Stress, and Depression for a Korean and a Korean-American Sample. *Asian Journal of Counselling* **13**, 235–270 (2006).

379. Ji, X. Family Communication Patterns and Mental Well-Being of Chinese International Students During Covid-19 Pandemic: Examining Effects of Family Mobile Communication. (ProQuest Dissertations and Theses, 2021).

380. Jia, H. Investigating the acculturation strategies and adaptation outcomes of Chinese students in Germany. (Shanghai International Studies University, 2009).

381. Jiang, M. Chinese students' adjustment to studying in UK Higher Education : academic self-efficacy and

psychological well-being. (University of York, 2018).

382. Jibeen, T. Subjective Well-Being of Afghan Refugees in Pakistan: The Moderating Role of Perceived Control in Married Men. *Community mental health journal* **55**, 144-155 (2019).

383. Jibeen, T. & Khalid, R. Predictors of Psychological well-being of Pakistani Immigrants in Toronto, Canada. *International Journal of Intercultural Relations* **34**, 452-464 (2010).

384. Johnson, E. C., Kristof-brown, A. L., Van vianen, A. E. M., De pater, I. E. & Klein, M. R. Expatriate Social Ties: Personality Antecedents and Consequences for Adjustment. *International Journal of Selection and Assessment* **11**, 277-288 (2003).

385. Jore, T., Oppedal, B. & Biele, G. Social anxiety among unaccompanied minor refugees in Norway.The association with pre-migration trauma and post-migration acculturation related factors. *Journal of psychosomatic research* **136**, 110175 (2020).

386. Jorgenson, K. C. & Nilsson, J. E. The Relationship Among Trauma, Acculturation, and Mental Health Symptoms in Somali Refugees. *The Counseling Psychologist* **49**, 196-232 (2021).

387. Jou, Y. H. & Fukada, H. Stress and social support in mental and physical health of Chinese students in Japan. *Psychological Reports* **81**, 1303-1312 (1997).

388. Jung, E., Hecht, M. L. & Wadsworth, B. C. The role of identity in international students' psychological well-being in the United States: A model of depression level, identity gaps, discrimination, and acculturation. *International Journal of Intercultural Relations* **31**, 605-624 (2007).

389. Jung, E., Zhang, Y., Hwang, W. & Zhang, Y. Parental Health and Children's Functioning in Immigrant Families: Family Roles and Perceived Treatment at School. *Journal of Child and Family Studies* **27**, 1899-1913 (2018).

390. Jung, M. Y., *et al.* Racial Discrimination and Health-Related Quality of Life: An Examination Among Asian American Immigrants. *Journal of Racial and Ethnic Health Disparities* **9**, 1262-1275 (2022).

391. Jurcik, T., *et al.* Unraveling Ethnic Density Effects, Acculturation, and Adjustment: The Case of Russian-Speaking Immigrants from the Former Soviet Union. *Journal of Community Psychology* **43**, 628-648 (2015).

392. Kabir, R., *et al.* Depression among the Non-Native International Undergraduate Students Studying Dentistry in Bangladesh. *International journal of environmental research and public health* **18**(2021).

393. Kaduvettoor-Davidson, A. & Inman, A. G. South Asian Americans: Perceived Discrimination, Stress, and Well-Being. *Asian American Journal of Psychology* **4**, 155–165 (2013).

394. Kahn, S. V. The influence of pre-migration factors and post-migration climate of the receiving community on the psychological distress of Latino immigrants. (University of Maryland, 2016).

395. Kaiser, B. N., Keys, H. M., Foster, J. & Kohrt, B. A. Social stressors, social support, and mental health among Haitian migrants in the Dominican Republic. *Revista Panamericana de Salud Publica|Pan American Journal of Public Health* **38.0**, 157-162 (2015).

396. Kaiyong, C. A study on acculturation strategies of international students in China. (Shanghai International Studies University, 2016).

397. Kamardeen, I. & Sunindijo, R. Y. Stressors Impacting the Performance of Graduate Construction Students: Comparison of Domestic and International Students. *Journal of Professional Issues in Engineering Education and Practice* **144**(2018).

398. Karatas, S., Crocetti, E., Schwartz, S. J. & Rubini, M. Psychological and social adjustment in refugee adolescents: The role of parents' and adolescents' friendships. *New Dir Child Adolesc Dev* **2021**, 123-139 (2021).

399. Kartal, D., Alkemade, N., Eisenbruch, M. & Kissane, D. Traumatic exposure, acculturative stress and cultural orientation: the influence on PTSD, depressive and anxiety symptoms among refugees. *Social Psychiatry and Psychiatric Epidemiology* **53**, 931-941 (2018).

400. Kartal, D., Alkemade, N. & Kiropoulos, L. Trauma and Mental Health in Resettled Refugees: Mediating Effect of Host Language Acquisition on Posttraumatic Stress Disorder, Depressive and Anxiety Symptoms. *Transcultural psychiatry* **56**, 3-23 (2019).

401. Kashima, E. S. & Abu-Rayya, H. M. Longitudinal Associations of Cultural Distance With Psychological Well-Being Among Australian Immigrants From 49 Countries. *Journal of Cross-Cultural Psychology* **45**, 587-600 (2014).

402. Kashima, E. S. & Loh, E. International students' acculturation: Effects of international, conational, and local ties and need for closure. *International Journal of Intercultural Relations* **30**, 471-485 (2006).

403. Kateri, E., Papastylianou, D. & Karademas, E. Perceived Discrimination and Psychological Well-Being Among Immigrants Living in Greece: Separation as Mediator and Interdependence as Moderator. *Europe's journal of psychology* **18**, 70-83 (2022).

404. Kateri, E. V., Tsouvelas, G. & Karademas, E. C. The role of acculturation attitudes and social support in anxiety and depression of Indian immigrants in Greece. *Psychiatriki* **30**, 311-319 (2019).

405. Kawai, N. & Mohr, A. The Contingent Effects of Role Ambiguity and Role Novelty on Expatriates' Work-related Outcomes. *British Journal of Management* **26**, 163-181 (2015).

406. Kawai, N. & Strange, R. Perceived organizational support and expatriate performance: understanding a mediated model. *The International Journal of Human Resource Management* **25**, 2438-2462 (2014).

407. Kegel, K. Homesickness and psychological distress in Asian international students: The potential mediating roles of social connectedness and Universal-Diverse orientation. (ProQuest Dissertations and Theses, 2015).

408. Keles, S., Friborg, O., Idsøe, T., Sirin, S. & Oppedal, B. Resilience and acculturation among unaccompanied refugee minors. *International Journal of Behavioral Development* **42**, 52-63 (2016).

409. Keles, S., Idsoe, T., Friborg, O., Sirin, S. & Oppedal, B. The Longitudinal Relation between Daily Hassles and Depressive Symptoms among Unaccompanied Refugees in Norway. *Journal of Abnormal Child Psychology* **45**, 1413-1427 (2017).

410. Khaled, S. M. & Gray, R. Depression in migrant workers and nationals of Qatar: An exploratory cross-cultural study. *International Journal of Social Psychiatry* **65**, 354-367 (2019).

411. Khamis, V. Psychological distress and neuroticism among Syrian refugee parents in post-resettlement contexts. *Journal of health psychology* **27**, 1149-1164 (2022).

412. Khan, S. & Haque, S. Trauma, mental health, and everyday functioning among Rohingya refugee people living in short- and long-term resettlements. *Social Psychiatry and Psychiatric Epidemiology* **56**, 497-512 (2021).

413. Khatiwada, J., Muzembo, B. A., Wada, K. & Ikeda, S. The effect of perceived social support on psychological distress and life satisfaction among Nepalese migrants in Japan. *PLoS One* **16**, e0246271 (2021).

414. Khawaja, N. G., Ibrahim, O. & Schweitzer, R. D. Mental Wellbeing of Students from Refugee and Migrant Backgrounds: The Mediating Role of Resilience. *School Mental Health* **9**, 284-293 (2017).

415. Khwaja, N. G. & Dempsey, J. Psychological Distress in;International University Students:;An Australian Study. *Australian Journal of Guidance and Counselling* **17**, 13-27 (2007).

416. Kiang, M. A School-Based Group Intervention for Immigrant Adolescents: Addressing Acculturative Stress and Facilitating Ethnic Identity Development, School Connectedness, and Related Psychosocial Outcomes. (ProQuest Dissertations and Theses, 2020).

417. Killian, K. D., Lehr, S. & Lehr, S. The resettlement blues: The role of social support in newcomer women's mental health. in *Springer international publishing* 97-109 (2016).

418. Kim, B. J., Chen, L., Lee, Y. & Xu, L. Quality of life of elderly Chinese immigrants: Focusing on living arrangements and social capital. *Educational Gerontology* **45**, 377-389 (2019).

419. Kim, B. J., Harris, L. M. & Cha, Y. Differences in the relationship between depression and self-rated life satisfaction in older Korean and Chinese immigrants. *Social Work in Mental Health* **15**, 555-566 (2017).

420. Kim, B. J., Linton, K. F. & Lum, W. Social capital and life satisfaction among Chinese and Korean elderly immigrants. *Journal of Social Work* **15**, 87-100 (2015).

421. Kim, E., Hogge, I. & Salvisberg, C. Effects of self-esteem and ethnic identity: Acculturative stress and psychological well-being among Mexican immigrants. *Hispanic Journal of Behavioral Sciences* **36**, 144-163 (2014).

422. Kim, E., Yun, M., Jun, J. Y. & Park, W.-S. Pre-migration trauma, repatriation experiences, and PTSD among North Korean refugees. *Journal of Immigrant and Minority Health* **21**, 466-472 (2019).

423. Kim, H. J., Pearce, M. & Choi-Kwon, S. Religious attendance, health-promoting lifestyle behaviors, and depressive symptoms among Koreans in the United Arab Emirates (UAE). *Journal of immigrant and minority health* **17**, 1098-1104 (2015).

424. Kim, I. Beyond Trauma: Post-resettlement Factors and Mental Health Outcomes Among Latino and Asian Refugees in the United States. *Journal of Immigrant and Minority Health* **18**, 740-748 (2016).

425. Kim, I. Behavioral health symptoms among refugees from Burma: Examination of sociodemographic and migration-related factors. *Asian American Journal of Psychology* **9**, 179 (2018).

426. Kim, I., Kang, S.-Y. & Kim, W. The effects of religious participation and familial assistance on mental health among older Chinese and Korean immigrants: Multiple mediator analyses. *Journal of Cross-Cultural Gerontology* **33**, 411-425 (2018).

427. Kim, I., Keovisai, M., Kim, W., Richards-Desai, S. & Yalim, A. C. Trauma, Discrimination, and Psychological Distress Across Vietnamese Refugees and Immigrants: A Life Course Perspective. *Community mental health journal* **55**, 385-393 (2019).

428. Kim, I.-H. & Noh, S. Changes in life satisfaction among Korean immigrants in Canada. *International Journal of Culture and Mental Health* **8**, 60-71 (2015).

429. Kim, I.-H. & Noh, S. Racial/ethnic variations in the main and buffering effects of ethnic and nonethnic supports on depressive symptoms among five ethnic immigrant groups in Toronto. *Ethnicity & Health* **21**, 215-232 (2016).

430. Kim, J. The role of social ecological factors in shaping leisure time physical activity and mental health among Asian immigrants in the United States. (Penn State University, 2018).

431. Kim, K. & Slocum, J. W., Jr. Individual differences and expatriate assignment effectiveness: The case of U.S.-based Korean expatriates. *Journal of World Business* **43**, 109-126 (2008).

432. Kim, M. A., Ham, O. K., Cho, I., Lee, E. J. & Lee, B. G. Level of Acculturation and Acculturative Stress Perceived by Asian Immigrant Women Married to South Korean Men. *Journal of Transcultural Nursing* **33**, 49-56 (2022).

433. Kim, S. The effect of social network sites use on international students' identity management and cross-cultural adjustment in the US. (ProQuest Dissertations and Theses, 2020).

434. Kim, S. J. & Yoo, I. Y. Health Promotion Behavior of Chinese International Students in Korea Including Acculturation Factors: A Structural Equation Model. *Asian nursing research* **10**, 25-31 (2016).

435. Kim, W., Kim, I., Lin, L., Baltimore, K. & Lin, L. Social Determinants of Mental Health Among Karen Refugees from Burma. *Community mental health journal* **58**, 749-760 (2022).

436. Kim, Y. Testing the mediating effects of resilience and mental health on the relationship between acculturative stress and binge drinking among international students. (The University of Texas at Arlington, 2016).

437. Kim, Y.*, et al.* Don't ask for fair treatment? A gender analysis of ethnic discrimination, response to discrimination, and self-rated health among marriage migrants in South Korea. *International journal for equity in health* **15**, 112 (2016).

438. Kim, Y. J., Cho, Y.-A. & Kim, H. A. A mediation effect of ego resiliency between stresses and mental health of North Korean refugee youth in South Korea. *Child and Adolescent Social Work Journal* **32**, 481-490 (2015).

439. Kim, Y. K., Maleku, A., Lemieux, C. M., Du, X. & Chen, Z. Behavioral Health Risk and Resilience among International Students in the United States: A Study of Socio-demographic Differences. *Journal of International Students* **9**, 282-305 (2019).

440. Kim, Y. Y. & McKay-Semmler, K. Social engagement and cross-cultural adaptation: An examination of direct- and mediated interpersonal communication activities of educated non-natives in the United States. *International Journal of Intercultural Relations* **37**, 99-112 (2013).

441. Kline, S. L. & Liu, F. The influence of comparative media use on acculturation, acculturative stress, and family relationships of Chinese international students. *International Journal of Intercultural Relations* **29**, 367-390 (2005).

442. Klokgieters, S. S., van Tilburg, T. G., Deeg, D. J. H. & Huisman, M. Do religious activities among young–old immigrants act as a buffer against the effect of a lack of resources on well-being? *Aging & Mental Health* **23**, 625-632 (2019).

443. Knipscheer, J. W., Sleijpen, M., Mooren, T., Ter Heide, F. J. J. & Van der Aa, N. Trauma exposure and refugee status as predictors of mental health outcomes in treatment-seeking refugees. *BJPsych bulletin* **39**, 178-182 (2015).

444. Ko, J., Frey, J. J., Osteen, P. & Ahn, H. Moderating effects of immigrant status on determinants of job satisfaction: Implications for occupational health. *Journal of Career Development* **42**, 396-411 (2015).

445. Kogan, I., Shen, J. & Siegert, M. What makes a satisfied immigrant? Host-country characteristics and immigrants' life satisfaction in eighteen European countries. *Journal of Happiness Studies* **19**, 1783-1809 (2018).

446. Koo, K., Nyunt, G. & Wang, B. Who Spends Too Much Time Online? *Journal of International Students* **11**, 122-143 (2021).

447. Koo Moon, H., Kwon Choi, B. & Shik Jung, J. Previous International Experience, Cross-Cultural Training and Expatriates' Cross-Cultural Adjustment. *HUMAN RESOURCE DEVELOPMENT QUARTERLY* **23**, 285-330 (2012).

448. Koveshnikov, A., Lehtonen, M. J. & Wechtler, H. Expatriates on the run: The psychological effects of the COVID-19 pandemic on expatriates' host country withdrawal intentions. *International Business Review* **31**, 102009 (2022).

449. Koveshnikov, A., Wechtler, H. & Dejoux, C. Cross-cultural adjustment of expatriates: The role of emotional intelligence and gender. *Journal of World Business* **49**, 362-371 (2014).

450. Kraimer, M. L. & Wayne, S. J. An Examination of Perceived Organizational Support as a Multidimensional Construct in the Context of an Expatriate Assignment. *Journal of Management* **30**, 209-237 (2004).

451. Kraimer, M. L., Wayne, S. J. & Jaworski, R. A. A. Sources Of Support and Expatriate Performance: The Mediating Role of Expatriate Adjustment. *Personnel Psychology* **54**, 71-99 (2001).

452. Kumpikaite-Valiuniene, V., Zickute, I., Baneviciene, I., Gao, J. & Torres, D. The Impact That Different Types of Organizational Cultures Have on the Adjustment of Self-Initiated Expatriates. *Frontiers in Psychology* **12**, 804947 (2021).

453. Kurt, G.*, et al.* The psychological impacts of COVID-19 related stressors on Syrian refugees in Turkey: The role of resource loss, discrimination, and social support. *International Journal of Intercultural Relations* **85**, 130-140 (2021).

454. Kwak, K. An evaluation of the healthy immigrant effect with adolescents in Canada: Examinations of gender and length of residence. *Social Science & Medicine* **157**, 87-95 (2016).

455. Ladum, A. & Burkholder, G. J. Psychological Adaptation of International Students in the Northern Part of Cyprus. *Higher Learning Research Communications* **9**, n1 (2019).

456. Lam, B. T. Impact of Perceived Racial Discrimination and Collective Self-Esteem on Psychological Distress Among Vietnamese-American College Students: Sense of Coherence as Mediator. *American Journal of Orthopsychiatry* **77**, 370-376 (2007).

457. Lashari, S. A., Awang-Hashim, R., Lashari, T. A. & Kaur, A. Acculturation stress and social support for international students' adjustment in Malaysia: does language proficiency matter? *Journal of Applied Research in Higher Education* **15**, 496-508 (2022).

458. Lashari, S. A., Kaur, A. & Awang-Hashim, R. Home Away from Home - The Role of Social Support for International Students' Adjustment. *Malaysian Journal of Learning and Instruction* **15**, 33-54 (2018).

459. Lauring, J. & Selmer, J. Adjustment of spouses of self-initiated expatriates: feeling different vs. feeling welcome. *Work and Family Interface in the International Career Context*, 117-138 (2015).

460. Le, H., Jiang, Z. & Nielsen, I. Cognitive Cultural Intelligence and Life Satisfaction of Migrant Workers: The Roles of Career Engagement and Social Injustice. *Social Indicators Research* **139**, 237-257 (2016).

461. Le, H., Jiang, Z. & Radford, K. Leader-member exchange and subjective well-being: the moderating role of metacognitive cultural intelligence. *Personnel Review* **50**, 954-970 (2020).

462. Le, Y. K., Snodgrass, J. L., Fenzel, L. M. & Tran, T. V. Acculturative stress and coping processes among middle-aged Vietnamese-born American Catholics: The roles of spirituality, religiosity, and resilience on well-being. *Asian American Journal of Psychology* **12**, 100-109 (2021).

463. Lecerof, S. S., Stafstrom, M., Westerling, R. & Ostergren, P. O. Does social capital protect mental health among migrants in Sweden? *Health promotion international* **31**, 644-652 (2016).

464. Lee, C., Sung, Y. T., Zhou, Y. & Lee, S. The relationships between the seriousness of leisure activities, social support and school adaptation among Asian international students in the U.S. *Leisure Studies* **37**, 197-210 (2017).

465. Lee, E.-J., Lee, L. & Jang, J. Internet for the internationals: effects of internet use motivations on international students' college adjustment. *Cyberpsychology, behavior and social networking* **14**, 433-437 (2011).

466. Lee, J. & Ciftci, A. Asian international students' socio-cultural adaptation: Influence of multicultural personality, assertiveness, academic self-efficacy, and social support. *International Journal of Intercultural Relations* **38**, 97-105 (2014).

467. Lee, J.-S., Koeske, G. F. & Sales, E. Social support buffering of acculturative stress: a study of mental health symptoms among Korean international students. *International Journal of Intercultural Relations* **28**, 399-414 (2004).

468. Lee, J. E., Kim, M. T. & Han, H. R. Correlates of Health-Related Quality of Life Among Korean Immigrant Elders. *Journal of Applied Gerontology* **34**, 844-857 (2015).

469. Lee, J. H. Depression among Korean Immigrants: The influence of acculturation and social support. (ProQuest Dissertations and Theses, 2016).

470. Lee, J. Y., Shin, S. S. & Lee, S. H. Quality of Life for North Korean Female Refugees: The Influence of Physical Health, PTSD, and Social Support. *Journal for Social Action in Counseling and Psychology* **11.0**, 2-13 (2019).

471. Lee, K. H. & Hwang, M. J. Private religious practice, spiritual coping, social support, and health status among older Korean adult immigrants. *Social work in public health* **29**, 428-443 (2014).

472. Lee, L. Y. & Kartika, N. The influence of individual, family, and social capital factors on expatriate adjustment and performance: The moderating effect of psychology contract and organizational support. *Expert Systems with Applications* **41**, 5483-5494 (2014).

473. Lee, L. Y., Veasna, S. & Wu, W. Y. The effects of social support and transformational leadership on expatriate adjustment and performance. *Career Development International* **18**, 377-415 (2013).

474. Lee, M., Nezu, A. M. & Nezu, C. M. Acculturative Stress, Social Problem Solving, and Depressive Symptoms among Korean American Immigrants. *Transcultural psychiatry* **55**, 710-729 (2018).

475. Lee, M. C., *et al.* Psychological distress among Chinese immigrants to the USA. *International Journal of*

Culture and Mental Health **8**, 150-161 (2014).

476. Lee, M. J. Acculturation-related stressors and Latino immigrant adolescent depressive symptoms: A multiple mediation analysis of stressors and resources by gender and immigrant generational status. (University of Illinois, 2016).

477. Lee, M. K., Kim, O., Kim, K. A. & Chu, S. H. Factors associated with posttraumatic growth among North Korean defectors in South Korea. *Scientific reports* **12**, 3989 (2022).

478. Lee, S. A., Park, H. S. & Kim, W. Gender differences in international students' adjustment. *College Student Journal* **43**, 1217-1227 (2009).

479. Lee, Y. & Im, E. O. A path analysis of stress and premenstrual symptoms in Korean international and Korean domestic students. *Journal of Advanced Nursing* **72**, 3045-3059 (2016).

480. Leiler, A., Bjarta, A., Ekdahl, J. & Wasteson, E. Mental health and quality of life among asylum seekers and refugees living in refugee housing facilities in Sweden. *Social Psychiatry and Psychiatric Epidemiology* **54**, 543-551 (2019).

481. Leong, C.-H. Predictive validity of the Multicultural Personality Questionnaire: A longitudinal study on the socio-psychological adaptation of Asian undergraduates who took part in a study-abroad program. *International Journal of Intercultural Relations* **31**, 545-559 (2007).

482. Leong, C.-H. & Ward, C. Identity conflict in sojourners. *International Journal of Intercultural Relations* **24**, 763-776 (2000).

483. Leshchyna, I. V., Asieieva, Y. O., Vasylieva, O. V., Strelnikova, I. M. & Kovalska, N. A. Adjustment disorders in international students studying in English during a pandemic. *Revista Amazonia Investiga* **10**, 200-208 (2021).

484. Letiecq, B. L., Grzywacz, J. G., Gray, K. M. & Eudave, Y. M. Depression among Mexican men on the migration frontier: the role of family separation and other structural and situational stressors. *Journal of Immigrant and Minority Health* **16**, 1193-1200 (2014).

485. Letiecq, B. L., *et al.* Central American Immigrant Mothers' Mental Health in the Context of Illegality: Structural Stress, Parental Concern, and Trauma. *Family & Community Health* **42**, 271-282 (2019).

486. Leung, C. The psychological adaptation of overseas and migrant students in Australia. *International Journal of Psychology* **36**, 251-259 (2001).

487. Li, A. & Gasser, M. B. Predicting Asian international students' sociocultural adjustment: A test of two mediation models. *International Journal of Intercultural Relations* **29**, 561-576 (2005).

488. Li, J. M., Froese, F. J. & Schmid, J. S. All or nothing: ambivalent acculturation strategies and job satisfaction of bicultural migrants in South Korea. *Asia Pacific Business Review* **29**, 719-739 (2021).

489. Li, M. The role of adul the role of adult attachment in in t in international students' acculturation process. (University of Kentucky, 2016).

490. Li, M. & Anderson, J. G. Pre-migration Trauma Exposure and Psychological Distress for Asian American Immigrants: Linking the Pre- and Post-migration Contexts. *Journal of Immigrant and Minority Health* **18**, 728-739 (2016).

491. Li, Q., Chi, P., Hall, B. J., Wu, Q. & Du, H. Job stress and depressive symptoms among migrant workers in Macau: A moderated mediation model of self-esteem and perceived social support. *PsyCh journal* **8**, 307-317 (2019).

492. Li, Y., Hofstetter, C. R., Irving, V., Chhay, D. & Hovell, M. F. Stress, illness, and the social environment: depressive symptoms among first generation mandarin speaking Chinese in greater Los Angeles. *Journal of Immigrant and Minority Health* **16**, 1035-1044 (2014).

493. Li, Y.*, et al.* Social Support, Attachment Closeness, and Self-Esteem Affect Depression in International Students in China. *Frontiers in Psychology* **12**, 618105 (2021).

494. Li, Y. Q. Perceived social support and psychological well-being of international students: The mediating effects of resiliency and spirituality. *Qi, Li Yue and Roslan, Samsilah and Zaremohzzabieh, Zeinab (2021) Perceived social support and psychological well-being of international students: the mediating effects of resiliency and spirituality/Li Yue Qi, Samsilah Roslan and Zeinab Zaremohzzabieh. Asian Journal of University Education (AJUE)* (2021).

495. Lian, Y. & Tsang, K.-K. The Impacts of Acculturation Strategies and Social Support on the Cross-cultural Adaptation of Mainland Chinese Students in Hong Kong. *Educational Research Journal* **25**, 81-102

(2010).

496. Liao, K. Y. & Wei, M. Academic stress and positive affect: Asian value and self-worth contingency as moderators among Chinese international students. *Cultural Diversity and Ethnic Minority Psychology* **20**, 107-115 (2014).

497. Liebkind, K. & Jasinskaja-Lahti, I. The influence of experiences of discrimination on psychological stress: a comparison of seven immigrant groups. *Journal of Community & Applied Social Psychology* **10**, 1-16 (2000).

498. Lim, Y. Relationship between Marriage Immigrant Mothers' Acculturative Stress and Their Adolescent Children's Career Decidedness in South Korea: Mediating Roles of Parenting and School Adjustment. *Sustainability* **13**(2021).

499. Lin, C.*, et al.* Prevalence and correlates of depression and anxiety among Chinese international students in US colleges during the COVID-19 pandemic: A cross-sectional study. *Plos One* **17**, e0267081 (2022).

500. Lin, E.-Y. Family and social influences on identity conflict in overseas Chinese. *International Journal of Intercultural Relations* **32**, 130-141 (2008).

501. Lin, J.-H., Peng, W., Kim, M., Kim, S. Y. & LaRose, R. Social networking and adjustments among international students. *New Media & Society* **14**, 421-440 (2011).

502. Lincoln, A. K., Lazarevic, V., White, M. T. & Ellis, B. H. The Impact of Acculturation Style and Acculturative Hassles on the Mental Health of Somali Adolescent Refugees. *Journal of Immigrant and Minority Health* **18**, 771-778 (2016).

503. Lippke, S., Schalk, T. M., Kuhnen, U. & Shang, B. Pace of life and perceived stress in international students. *PsyCh journal* **10**, 425-436 (2021).

504. Littrell, L. Mentoring expatriate employees: The influence of multiple mentors on overseas experiences. (2007).

505. Liu, S., He, L., Wei, M., Du, Y. & Cheng, D. Depression and anxiety from acculturative stress: Maladaptive perfectionism as a mediator and mindfulness as a moderator. *Asian American Journal of Psychology* **13**, 207 (2022).

506. Liu, T., Wong, Y. J. & Tsai, P. C. Conditional Mediation Models of Intersecting Identities Among Female Asian International Students. *The Counseling Psychologist* **44**, 411-441 (2016).

507. Liu, X. & Shaffer, M. A. An Investigation of Expatriate Adjustment and Performance: A Social Capital Perspective. *International Journal of Cross Cultural Management* **5**, 235-254 (2005).

508. Liu, Y. Expatriates' dual commitment: a serial multiple mediation model of workplace friendships and adjustment. *Personnel Review* **51**, 1690-1706 (2021).

509. Liu, Y.*, et al.* Path Analysis of Acculturative Stress Components and Their Relationship with Depression Among International Students in China. *Stress Health* **32**, 524-532 (2016).

510. Lo, S. F., Chang, L.-J., Hayter, M., & O Yang, A.-C. An Exploration of Factors Related to Quality of Life in Indonesian Care Workers in Home-Based Care Settings. *The Journal of Nursing Research* **27**, e47 (2019).

511. Loayza-Rivas, J. & Fernández-Castro, J. Perceived stress and well-being: The role of social support as a protective factor among Peruvian immigrants in Spain. *Ansiedad y Estrés* **26**, 67-72 (2020).

512. Löbel, L. M. Family separation and refugee mental health–A network perspective. *Social Networks* **61**, 20-33 (2020).

513. Logan, J. G., Barksdale, D. J., James, S. A. & Chien, L. C. John Henryism Active Coping, Acculturation, and Psychological Health in Korean Immigrants. *Journal of Transcultural Nursing* **28**, 168-178 (2017).

514. Loi, R., Ao, O. K. Y. & Xu, A. J. Perceived organizational support and coworker support as antecedents of foreign workers' voice and psychological stress. *International Journal of Hospitality Management* **36**, 23-30 (2014).

515. Lommel, L. L. Applying intersectionality and acculturation theories to explain disparities in self-rated health among Asian and Hispanic immigrants in the U.S. (ProQuest Dissertations and Theses, 2016).

516. Lopez-Rodriguez, L., Navas, M., Cuadrado, I. & Tatar, M. Adjustment Outcomes of Native and Immigrant Youth in Spain: A Mediation Model. *The Spanish Journal of Psychology* **21**, E19 (2018).

517. Lorenzo-Blanco, E. I.*, et al.* Longitudinal Effects of Latino Parent Cultural Stress, Depressive Symptoms, and Family Functioning on Youth Emotional Well-Being and Health Risk Behaviors. *Family process* **56**,

981-996 (2017).

518. Lorenzo-Blanco, E. I., *et al.* Cultural Stress, Emotional well-being, and Health Risk Behaviors among Recent Immigrant Latinx families: The Moderating Role of Perceived Neighborhood Characteristics. *Journal of youth and adolescence* **48**, 114-131 (2019).

519. Lou, N. M. Acculturation in a postcolonial context: Language, identity, cultural adaptation, and academic achievement of Macao students in Mainland China. *International Journal of Intercultural Relations* **85**, 213-225 (2021).

520. Lou, N. M. & Noels, K. A. Sensitivity to Language-based Rejection in Intercultural Communication: The Role of Language Mindsets and Implications for Migrants' Cross-cultural Adaptation. *Applied Linguistics* **40**, 478-505 (2019).

521. Lowinger, R. J., *et al.* Predictors of Academic Procrastination in Asian International College Students. *Journal of Student Affairs Research and Practice* **53**, 90-104 (2016).

522. Lu, L., Wang, X., Wang, X., Guo, X. & Pan, B. Association of Covid-19 pandemic-related stress and depressive symptoms among international medical students. *BMC Psychiatry* **22**, 20 (2022).

523. Luksyte, A., Spitzmueller, C. & Rivera-Minaya, C. Y. Factors relating to wellbeing of foreign-born Hispanic workers. *Journal of Managerial Psychology* **29**, 685-704 (2014).

524. Lumley, M., Katsikitis, M. & Statham, D. Depression, anxiety, and acculturative stress among resettled Bhutanese refugees in Australia. *Journal of Cross-Cultural Psychology* **49**, 1269-1282 (2018).

525. Luo, Y. & Sato, Y. Health-Related Quality of Life and Risk Factors among Chinese Women in Japan Following the COVID-19 Outbreak. *International journal of environmental research and public health* **18**(2021).

526. Luo, Z., Wu, S., Fang, X. & Brunsting, N. C. International Students' Perceived Language Competence, Domestic Student Support, and Psychological Well-Being at a U.S. University. *Journal of International Students* **9.0**, 954-971 (2019).

527. Ma, C. & Zhou, C. Understanding and Managing the Belt and Road International Students in China. in *Proceedings of the 2020 4th International Conference on Deep Learning Technologies (ICDLT)* 84-88

(2020).

528. Ma, K. Acculturation stress and depression among first-year international graduate students from China and India at the University of South Carolina. (ProQuest Dissertations and Theses, 2017).

529. Ma, S., Zhu, Y. & Bresnahan, M. Chinese International Students' Face Concerns, Self-Stigma, Linguistic Factors, and Help-Seeking Intentions for Mental Health. *Health Communication* **37**, 1631-1639 (2022).

530. Machul, M*., et al.* Lifestyle Practices, Satisfaction with Life and the Level of Perceived Stress of Polish and Foreign Medical Students Studying in Poland. *International journal of environmental research and public health* **17**(2020).

531. Madi, D., Bobowik, M., Verkuyten, M. & Basabe, N. Social intergroup and temporal intrapersonal comparisons: Responses to perceived discrimination and protective mechanisms of eudaimonic well-being. *International Journal of Intercultural Relations* **86**, 74-84 (2022).

532. Mahajan, A. & Toh, S. M. Facilitating expatriate adjustment: The role of advice-seeking from host country nationals. *Journal of World Business* **49**, 476-487 (2014).

533. Maharjan, M. P., Stoermer, S. & Froese, F. J. Research productivity of self-initiated expatriate academics: Influences of job demands, resources and cross-cultural adjustment. *European Management Review* **19**, 285-298 (2021).

534. Mahmood, H. & Burke, M. G. Analysis of Acculturative Stress and Sociocultural Adaptation Among International Students at a Non-Metropolitan University. *Journal of International Students* **8.0**, 284-307 (2018).

535. Maiya, S., Carlo, G., Davis, A. N. & Streit, C. Relations among acculturative stress, internalizing symptoms, and prosocial behaviors in Latinx college students. *Journal of Latinx Psychology* **9**, 77-91 (2021).

536. Mak, A. S., Bodycott, P. & Ramburuth, P. Beyond Host Language Proficiency. *Journal of Studies in International Education* **19**, 460-475 (2015).

537. Mak, A. S. & Kim, I. Korean International Students' Coping Resources and Psychological;Adjustment in Australia. *OMNES: The Journal of Multicultural Society* **2**(2011).

538. Mak, A. S. & Nesdale, D. Migrant Distress: The Role of Perceived Racial Discrimination and Coping Resources. *Journal of Applied Social Psychology* **31**, 2632-2647 (2001).

539. Maldonado, A. I., Cunradi, C. B. & Napoles, A. M. Racial/Ethnic Discrimination and Intimate Partner Violence Perpetration in Latino Men: The Mediating Effects of Mental Health. *International journal of environmental research and public health* **17**(2020).

540. Malek, M. A. Effect of support and cultural intelligence on the adjustment and performance of expatriates and their family members in Malaysia. (Aston University, 2011).

541. Maleku, A., *et al.* The hidden minority: Discrimination and mental health among international students in the US during the COVID-19 pandemic. *Health & Social Care in the Community* **30**, e2419-e2432 (2022).

542. Malm, A., Tinghog, P., Narusyte, J. & Saboonchi, F. The refugee post-migration stress scale (RPMS) - development and validation among refugees from Syria recently resettled in Sweden. *Conflict and health* **14**, 2 (2020).

543. Manzoor, F., Wei, L. & Haq, M. Z. U. Effect of Coronavirus-19 on Mental Condition of International Students in China. *Front Psychiatry* **12**, 738828 (2021).

544. Mao, W., Guo, M., Xu, L., Liu, J. & Chi, I. Intergenerational support and self-rated health among older Chinese immigrants: Do depressive symptoms play a mediating role? *China Journal of Social Work* **14**, 17-33 (2020).

545. Marchante-Hoffman, A. N. Giving Voice to Underserved, Foreign-Born Latino Youth: Trauma, Stress, and Health in the Primary Care Setting. (ProQuest Dissertations and Theses, 2018).

546. Martinet, É. H. G. & Damásio, B. F. Relationships between Cultural Adaptation and Immigrants' Well-Being. *Psico-USF* **26**, 467-481 (2021).

547. Martínez García, M. F., García Ramírez, M. & Maya Jariego, I. Social support and locus of control as predictors of psychological well-being in Moroccan and Peruvian immigrant women in Spain. *International Journal of Intercultural Relations* **26**, 287-310 (2002).

548. Martos-Mendez, M. J., Garcia-Cid, A., Gomez-Jacinto, L. & Hombrados-Mendieta, I. Perceived Discrimination, Psychological Distress and Cardiovascular Risk in Migrants in Spain. *International*

journal of environmental research and public health **17**, 1-18 (2020).

549. Martynowska, K., Korulczyk, T. & Mamcarz, P. J. Perceived stress and well-being of Polish migrants in the UK after Brexit vote. *PLoS One* **15**, e0236168 (2020).

550. Masgoret, A. M. Examining the role of language attitudes and motivation on the sociocultural adjustment and the job performance of sojourners in Spain. *International Journal of Intercultural Relations* **30**, 311-331 (2006).

551. Mason, D. The relationship among acculturation orientation, perceived discrimination, psychological functioning and gender among South Asian and South East Asian international students in the United States of America. (University of Kentucky, 2017).

552. Matsumoto, M. & Gopal, B. Model of cross-cultural adjustment and view of life-career among Japanese expatriate spouses in India. *Current Psychology* **41**, 5935-5947 (2022).

553. Mayer, Y., Ilan, R., Slone, M. & Lurie, I. Relations between traumatic life events and mental health of Eritrean asylum-seeking mothers and their children's mental health. *Children and Youth Services Review* **116**(2020).

554. Mazzucato, V., Dito, B. B., Grassi, M. & Vivet, J. Transnational parenting and the well-being of Angolan migrant parents in Europe. *Global Networks* **17**, 89-110 (2017).

555. McGinley, J. Expatriate adjustment within a social context: Examination of a sample in Russia. *Journal of Social, Evolutionary, and Cultural Psychology* **2**, 56-68 (2008).

556. McGrath, M.*, et al.* Somatic distress among Syrian refugees in Istanbul, Turkey: A cross-sectional study. *Journal of psychosomatic research* **132**, 109993 (2020).

557. McKeering, P., Hwang, Y. S. & Ng, C. A study into wellbeing, student engagement and resilience in early-adolescent international school students. *Journal of Research in International Education* **20**, 69-92 (2021).

558. Meghani, D. T. & Harvey, E. A. Asian indian international students' trajectories of depression, acculturation, and enculturation. *Asian American Journal of Psychology* **7**, 1-14 (2016).

559. Mendoza, N. B., Mordeno, I. G., Latkin, C. A. & Hall, B. J. Evidence of the paradoxical effect of social

network support: A study among Filipino domestic workers in China. *Psychiatry research* **255**, 263-271 (2017).

560. Merced, K., *et al.* Spanish Translation and Psychometric Validation of a Measure of Acculturative Stress among Latinx Immigrants in the USA. *International journal of environmental research and public health* **19**(2022).

561. Merchant, H., Rao-Nicholson, R. & Iheikhena, E. G. A multi-dimensional analysis of the subjective well-being of self-initiated expatriates: The case of Nigerian expatriates in Germany. *Thunderbird International Business Review* **65**, 117-130 (2022).

562. Merilus, H. A. First year international students' interpersonal relations, external experiences, knowledge of immigration procedures, academic achievement, social-cultural adjustment, and their willingness to stay in the united states after they complete their education (Dowling College, 2015).

563. Mesch, G. S., Turjeman, H. & Fishman, G. Perceived Discrimination and the Well-being of Immigrant Adolescents. *Journal of Youth and Adolescence* **37**, 592-604 (2008).

564. Miconi, D., Moscardino, U., Ronconi, L. & Altoè, G. Perceived Parenting, Self-Esteem, and Depressive Symptoms in Immigrant and Non-Immigrant Adolescents in Italy: A Multigroup Path Analysis. *Journal of Child and Family Studies* **26**, 345-356 (2016).

565. Miller, E., Ziaian, T., de Anstiss, H. & Baak, M. Ecologies of Resilience for Australian High School Students from Refugee Backgrounds: Quantitative Study. *International journal of environmental research and public health* **19**(2022).

566. Miller, R., Ong, K. I. C., Choi, S., Shibanuma, A. & Jimba, M. Seeking connection: a mixed methods study of mental well-being and community volunteerism among international migrants in Japan. *BMC Public Health* **20**, 1272 (2020).

567. Misra, R., Crist, M. & Burant, C. J. Relationships Among Life Stress, Social Support, Academic Stressors, and Reactions to Stressors of International Students in the United States. *International Journal of Stress Management* **10**, 137-157 (2003).

568. Miyamoto, Y. & Kuhlman, N. Ameliorating culture shock in Japanese expatriate children in the US.

International Journal of Intercultural Relations **25**, 21-40 (2001).

569. Mohr, A. T. & Klein, S. Exploring the adjustment of American expatriate spouses in Germany. *The International Journal of Human Resource Management* **15**, 1189-1206 (2004).

570. Moinolmolki, N. The role of bhutanese adolescent refugees' acculturation and familial social capital on their general well-being and school adjustment. (ProQuest Dissertations and Theses, 2016).

571. Moinolmolki, N. Correlates of general wellbeing among Bhutanese youths. *Journal of Ethnic & Cultural Diversity in Social Work* **29**, 185-202 (2019).

572. Molsa, M., Kuittinen, S., Tiilikainen, M., Honkasalo, M. L. & Punamaki, R. L. Mental health among older refugees: the role of trauma, discrimination, and religiousness. *Aging Ment Health* **21**, 829-837 (2017).

573. Molsa, M.*, et al.* Mental and somatic health and pre- and post-migration factors among older Somali refugees in Finland. *Transcultural psychiatry* **51**, 499-525 (2014).

574. Moore, T. M. The sojourner's truth: Exploring bicultural identity as a predictor of assignment success in American expatriates. (2009).

575. Mora, D. C.*, et al.* Social isolation among Latino workers in rural North Carolina: exposure and health implications. *Journal of Immigrant and Minority Health* **16**, 822-830 (2014).

576. Morey, B. N., Valencia, C., Park, H. W. & Lee, S. The central role of social support in the health of Chinese and Korean American immigrants. *Social Science & Medicine* **284**, 114229 (2021).

577. Morgan, G., Melluish, S. & Welham, A. Exploring the relationship between postmigratory stressors and mental health for asylum seekers and refused asylum seekers in the UK. *Transcultural psychiatry* **54**, 653-674 (2017).

578. Mossakowski, K. N., Wongkaren, T., Hill, T. D. & Johnson, R. Does ethnic identity buffer or intensify the stress of discrimination among the foreign born and U.S. born? Evidence from the Miami-Dade Health Survey. *Journal of Community Psychology* **47**, 445-461 (2019).

579. Mougenot, B., Amaya, E., Mezones-Holguin, E., Rodriguez-Morales, A. J. & Cabieses, B. Immigration, perceived discrimination and mental health: evidence from Venezuelan population living in Peru. *Global Health* **17**, 8 (2021).

580. Mui, A. C. & Lee, E. S. Correlates of Depression Among Chinese and Korean Immigrant Elders in the United States. *Ageing International* **39**, 274-288 (2013).

581. Mukherjee, A. J. & Diwan, S. Late Life Immigration and Quality of Life among Asian Indian Older Adults. *Journal of cross-cultural gerontology* **31**, 237-253 (2016).

582. Muller, L. R. F., Buter, K. P., Rosner, R. & Unterhitzenberger, J. Mental health and associated stress factors in accompanied and unaccompanied refugee minors resettled in Germany: a cross-sectional study. *Child and adolescent psychiatry and mental health* **13**, 8 (2019).

583. Muller, L. R. F.*, et al.* 1-year follow-up of the mental health and stress factors in asylum-seeking children and adolescents resettled in Germany. *BMC Public Health* **19**, 908 (2019).

584. Mullins, M. W. Acculturation and adjustment of expatriates: From organizational climate to discrimination. (ProQuest Dissertations and Theses, 2018).

585. Murphy, J. E.*, et al.* Relationships Between English Language Proficiency, Health Literacy, and Health Outcomes in Somali Refugees. *Journal of Immigrant and Minority Health* **21**, 451-460 (2019).

586. Nahidi, S., Blignault, I., Hayen, A. & Razee, H. Psychological Distress in Iranian International Students at an Australian University. *Journal of Immigrant and Minority Health* **20**, 651-657 (2018).

587. Nam, B., Kim, J. Y., DeVylder, J. E. & Song, A. Family functioning, resilience, and depression among North Korean refugees. *Psychiatry research* **245**, 451-457 (2016).

588. Nesterko, Y., Ulitsa, N., Friedrich, M. & Glaesmer, H. Do They Feel the Same Way? Health-Related Quality of Life and Satisfaction With Life in Jewish Immigrants From the Former Soviet Union in Germany and Israel. *Journal of Cross-Cultural Psychology* **49**, 618-634 (2018).

589. Neto, F. Cultural competence among Ukrainian economic immigrants in Portugal. (ProQuest Dissertations and Theses, 2015).

590. Neto, F. Subjective Well-Being of Angolan Students in Portugal. *Journal of Studies in International Education* **24**, 456-473 (2019).

591. Neto, F. & da Conceição Pinto, M. Loneliness among Chinese Migrants in Portugal. *Journal of Chinese Overseas* **17**, 196-213 (2021).

592. Neto, F. & Guse, T. Predictors of mental health among Angolan migrants living in Portugal. *International Journal of Migration, Health and Social Care* **14**, 146-159 (2018).

593. Neto, F. & Wilks, D. C. Predictors of Psychological Adaptation of Cape Verdean Students in Portugal. *Journal of College Student Development* **58**, 1087-1100 (2017).

594. Neto, F., Wilks, D. C. & Fonseca, A. C. M. Job-Related Well-Being of Immigrants. *Social Indicators Research* **141**, 463-475 (2018).

595. Neto, J., Quintana-Orts, C. & Neto, F. Acculturation, adaptation, and loneliness among Cape Verdean immigrants. *International Journal of Intercultural Relations* **87**, 98-107 (2022).

596. Neville, J. Host country language effects on American self-initiated expatriates' adjustment in China, South Korea, and Japan. (ProQuest Dissertations and Theses, 2017).

597. Newman, A., Nielsen, I., Smyth, R. & Hirst, G. Mediating Role of Psychological Capital in the Relationship between Social Support and Wellbeing of Refugees. *International Migration* **56**, 117-132 (2018).

598. Newman, G. G. The relationship between acculturation and depression with Burmese refugees. (ProQuest Dissertations and Theses, 2023).

599. Newnham, E. A., Pearman, A., Olinga-Shannon, S. & Nickerson, A. The mental health effects of visa insecurity for refugees and people seeking asylum: a latent class analysis. *International journal of public health* **64**, 763-772 (2019).

600. Ng, I. F., Lee, S. Y., Wong, W. K. & Chou, K. L. Effects of Perceived Discrimination on the Quality of Life Among New Mainland Chinese Immigrants to Hong Kong: A Longitudinal Study. *Social Indicators Research* **120**, 817-834 (2014).

601. Ng, T. K., Tsang, K. K. & Lian, Y. Acculturation strategies, social support, and cross-cultural adaptation: a mediation analysis. *Asia Pacific Education Review* **14**, 593-601 (2013).

602. Ng, T. K., Wang, K. W. C. & Chan, W. Acculturation and cross-cultural adaptation: The moderating role of social support. *International Journal of Intercultural Relations* **59**, 19-30 (2017).

603. Ngo, H. Y. & Li, H. Cultural Identity and Adaptation of Mainland Chinese Immigrants in Hong Kong.

American Behavioral Scientist **60**, 730-749 (2016).

604. Nguyen, D. & Goel, M. Social determinants and the psychological distress of Vietnamese immigrants. *International Journal of Culture and Mental Health* **8**, 22-33 (2014).

605. Nguyen, M. H., Le, T. T. & Meirmanov, S. Depression, Acculturative Stress, and Social Connectedness among International University Students in Japan: A Statistical Investigation. *Sustainability* **11**(2019).

606. Nickerson, A., *et al.* Emotion dysregulation mediates the relationship between trauma exposure, post-migration living difficulties and psychological outcomes in traumatized refugees. *Journal of Affective Disorders* **173**, 185-192 (2015).

607. Nickerson, A., *et al.* Cognitive mechanisms underlying the association between trauma exposure, mental health and social engagement in refugees: A longitudinal investigation. *Journal of Affective Disorders* **307**, 20-28 (2022).

608. Nickerson, A., *et al.* The association between visa insecurity and mental health, disability and social engagement in refugees living in Australia. *European journal of psychotraumatology* **10**, 1688129 (2019).

609. Nickerson, A., *et al.* Longitudinal association between trust, psychological symptoms and community engagement in resettled refugees. *Psychological medicine* **49**, 1661-1669 (2019).

610. Nickerson, A., *et al.* Moral Injury in Traumatized Refugees. *Psychotherapy and psychosomatics* **84**, 122-123 (2015).

611. Niehoff, B. P. & Maciocha, A. An Initial Exploration of the Cultural Adaptation and Motivations of Immigrant Workers in Ireland. *Irish Journal of Management* **29**(2008).

612. Nobis, E. The moderating effect of humility on the relationship between racialized trauma and psychological outcomes in a Latinx immigrant population. (ProQuest Dissertations and Theses, 2021).

613. Noh, S. & Kaspar, V. Perceived Discrimination and Depression: Moderating Effects of Coping, Acculturation, and Ethnic Support. *American Journal of Public Health* **93**, 232-238 (2003).

614. Nonrawan, K. The relationship between Mandarin language ability and sociocultural adaptation with cultural intelligence as mediator: A case study of international students in Taiwan. (ProQuest Dissertations and Theses, 2019).

615. Noor, N. M. & Shaker, M. N. Perceived workplace discrimination, coping and psychological distress among unskilled Indonesian migrant workers in Malaysia. *International Journal of Intercultural Relations* **57**, 19-29 (2017).

616. Nugraha, S., Hirano, Y. & Sumihisha, H. The Change in Mental Health Status of Indonesian Health Care Migrant Worker in Japan. *Kesmas: National Public Health Journal* **12**, 53-89 (2017).

617. O'Donnell, A. W., Paolini, S. & Stuart, J. Distinct trajectories of psychological distress among resettled refugees: Community acceptance predicts resilience while low ingroup social support predicts clinical distress. *Transcultural psychiatry* **60**, 26-38 (2023).

618. O'Donnell, A. W., Stuart, J. & O'Donnell, K. J. The long-term financial and psychological resettlement outcomes of pre-migration trauma and post-settlement difficulties in resettled refugees. *Social Science & Medicine* **262**, 113246 (2020).

619. Ogunsanya, M. E., Bamgbade, B. A., Thach, A. V., Sudhapalli, P. & Rascati, K. L. Determinants of health-related quality of life in international graduate students. *Currents in Pharmacy Teaching and Learning* **10**, 413-422 (2018).

620. Oh, H., Ardelt, M. & Koropeckyj-Cox, T. Daughters' generation: The importance of having daughters living nearby for older Korean immigrants' mental health. *Journal of Family Issues* **38**, 2329-2345 (2017).

621. Oh, S.-Y. & Jang, K. Self-initiated expatriate adjustment: South Korean workers in Vietnam. *Career Development International* **26**, 16-43 (2021).

622. Olaniran, B. A. International students' network patterns and cultural stress: What really counts. *Communication Research Reports* **10**, 69-83 (1993).

623. Olvera, J. Does citizenship status, education, and medical insurance protect against psychological distress among adult Mexican immigrants? (ProQuest Dissertations and Theses, 2020).

624. Ong, A. S. J. & Ward, C. The Construction and Validation of a Social Support Measure for Sojourners: The Index of Sojourner Social Support (ISSS) Scale. *Journal of Cross-Cultural Psychology* **36**, 637-661 (2005).

625. Oppedal, B. & Idsoe, T. The role of social support in the acculturation and mental health of

unaccompanied minor asylum seekers. *Scandinavian journal of psychology* **56**, 203-211 (2015).

626. Oppedal, B., Özer, S. & Şirin, S. R. Traumatic events, social support and depression: Syrian refugee children in Turkish camps. *Vulnerable Children and Youth Studies* **13**, 46-59 (2018).

627. Ordonez, Z. Antecedents of expatriate organizational embeddedness: The role of perceived cultural distance and host country national support. (ProQuest Dissertations and Theses, 2017).

628. Orjiako, O.-E. Y. & So, D. The role of acculturative stress factors on mental health and help-seeking behavior of sub-Saharan African immigrants. *International Journal of Culture and Mental Health* **7**, 315-325 (2014).

629. Osman-Gani, A. M. & Rockstuhl, T. Cross-cultural training, expatriate self-efficacy, and adjustments to overseas assignments: An empirical investigation of managers in Asia. *International Journal of Intercultural Relations* **33**, 277-290 (2009).

630. Oxman-Martinez, J. & Choi, Y. R. Newcomer Children: Experiences of Inclusion and Exclusion, and Their Outcomes. *Social Inclusion* **2**, 23-37 (2014).

631. Oyeniyi, O., Smith, R. L., Watson, J. C. & Nelson, K. A comparison of first-year undergraduate and graduate international students' adjustment to college. (ProQuest Dissertations and Theses, 2019).

632. Ozer, S. Predictors of international students' psychological and sociocultural adjustment to the context of reception while studying at Aarhus University, Denmark. *Scandinavian journal of psychology* **56**, 717-725 (2015).

633. Pagel, L. & Edele, A. The role of different school organizational models in the psychological adaptation of refugee adolescents. *European Journal of Psychology of Education* **37**, 1069-1092 (2021).

634. Palacios, M. P., Patel, S. G., Reed II, D. E., Poe, C. H. & Barrera, A. Z. A mixed-methods approach to understanding complex risk among newcomer Latina immigrant adolescents. *Peace and Conflict: Journal of Peace Psychology* **26**, 414-426 (2020).

635. Palthe, J. The relative importance of antecedents to cross-cultural adjustment: implications for managing a global workforce. *International Journal of Intercultural Relations* **28**, 37-59 (2004).

636. Pan, J.-Y. A resilience-based and meaning-oriented model of acculturation: A sample of mainland Chinese

postgraduate students in Hong Kong. *International Journal of Intercultural Relations* **35**, 592-603 (2011).

637. Pan, J.-Y., Wong, D. F. K., Chan, C. L. W. & Joubert, L. Meaning of life as a protective factor of positive affect in acculturation: A resilience framework and a cross-cultural comparison. *International Journal of Intercultural Relations* **32**, 505-514 (2008).

638. Pan, J. Y. Predictors of post-migration growth for Chinese international students in Australia. *International Journal of Intercultural Relations* **47**, 69-77 (2015).

639. Pan, J. Y. & Keung Wong, D. F. Acculturative Stressors and Acculturative Strategies as Predictors of Negative Affect Among Chinese International Students in Australia and Hong Kong: A Cross-Cultural Comparative Study. *Academic Psychiarty* **35**, 376-381 (2011).

640. Pang, H. Understanding the effects of WeChat on perceived social capital and psychological well-being among Chinese international college students in Germany. *Aslib Journal of Information Management* **70**, 288-304 (2018).

641. Pang, H. Exploring the beneficial effects of social networking site use on Chinese students' perceptions of social capital and psychological well-being in Germany. *International Journal of Intercultural Relations* **67**, 1-11 (2018).

642. Pang, H. How does time spent on WeChat bolster subjective well-being through social integration and social capital? *Telematics and Informatics* **35**, 2147-2156 (2018).

643. Pang, H. Is active social media involvement associated with cross-culture adaption and academic integration among boundary-crossing students? *International Journal of Intercultural Relations* **79**, 71-81 (2020).

644. Pannetier, J., Lert, F., Jauffret Roustide, M. & du Lou, A. D. Mental health of sub-saharan african migrants: The gendered role of migration paths and transnational ties. *SSM-population health* **3**, 549-557 (2017).

645. Pantelidou, S. & Craig, T. K. J. Culture shock and social support. *Social Psychiatry and Psychiatric Epidemiology* **41**, 777-781 (2006).

646. Park, H.-S. & Rubin, A. The mediating role of acculturative stress in the relationship between

acculturation level and depression among Korean immigrants in the U.S. *International Journal of Intercultural Relations* **36**, 611-623 (2012).

647. Park, J., Jang, Y., Oh, H. & Chi, I. Loneliness as a Mediator in the Association Between Social Isolation and Psychological Distress: A Cross-Sectional Study With Older Korean Immigrants in the United States. *Research on Aging* **45**, 438-447 (2023).

648. Park, N. & Noh, H. Effects of mobile instant messenger use on acculturative stress among international students in South Korea. *Computers in Human Behavior* **82**, 34-43 (2018).

649. Park, N., Song, H. & Lee, K. M. Social networking sites and other media use, acculturation stress, and psychological well-being among East Asian college students in the United States. *Computers in Human Behavior* **36**, 138-146 (2014).

650. Park, N. S., Jang, Y., Chiriboga, D. A. & Chung, S. Profiles of Social Engagement and Their Association With Loneliness and Self-Rated Health Among Older Korean Immigrants. *Research on Aging* **42**, 300-311 (2020).

651. Parker, B. & McEvoy, G. M. Initial examination of a model of intercultural adjustment. *International Journal of Intercultural Relations* **17**, 355-379 (1993).

652. Parkhomenko, D. Quality of life and migration experiences among Russian speaking immigrants to the United States of America. (ProQuest Dissertations and Theses, 2016).

653. Patel, S. G., Tabb, K. M., Strambler, M. J. & Eltareb, F. Newcomer Immigrant Adolescents and Ambiguous Discrimination. *Journal of Adolescent Research* **30**, 7-30 (2014).

654. Paulus, P. & Muehlfeld, K. Host country language skills and expatriates' cross-cultural adjustment in the presence of fear of terror. *Journal of Global Mobility: The Home of Expatriate Management Research* **5**, 418-442 (2017).

655. Pedersen, E. R., Neighbors, C., Larimer, M. E. & Lee, C. M. Measuring Sojourner Adjustment among American students studying abroad. *International Journal of Intercultural Relations* **35**, 881-889 (2011).

656. Pekerti, A. A., van de Vijver, F. J. R., Moeller, M. & Okimoto, T. G. Intercultural contacts and acculturation resources among International students in Australia: A mixed-methods study. *International*

Journal of Intercultural Relations **75**, 56-81 (2020).

657. Peltokorpi, V. Cross-cultural adjustment of expatriates in Japan. *The International Journal of Human Resource Management* **19**, 1588-1606 (2008).

658. Perrucci, R. & Hu, H. Satisfaction with social and educational experiences among international graduate students. *Research in Higher Education* **36**, 491-508 (1995).

659. Pho, H. & Schartner, A. Social contact patterns of international students and their impact on academic adaptation. *Journal of Multilingual and Multicultural Development* **42**, 489-502 (2019).

660. Phua, D. Y., Meaney, M. J., Khor, C. C., Lau, I. Y. M. & Hong, Y. Y. Effects of bonding with parents and home culture on intercultural adaptations and the moderating role of genes. *Behavioural Brain Research* **325**, 223-236 (2017).

661. Pimentel-Narez, D. The effects of immigrant status, gender and years of U.S. residency on the acculturative stress and psychological symptomatology of Mexican immigrants. (ProQuest Dissertations and Theses, 2017).

662. Pinto, L. H., Cabral-Cardoso, C. & Werther, W. B. Adjustment elusiveness: An empirical investigation of the effects of cross-cultural adjustment on general assignment satisfaction and withdrawal intentions. *International Journal of Intercultural Relations* **36**, 188-199 (2012).

663. Podsiadlowski, A., Vauclair, C.-M., Spiess, E. & Stroppa, C. Social support on international assignments: The relevance of socioemotional support from locals. *International Journal of Psychology* **48**, 563-573 (2013).

664. Polek, E. & Schoon, I. The Sociocultural Adaptation of Polish Brides in the Netherlands: Marital and Occupational Status. *Journal of Comparative Family Studies* **39**, 353-370 (2008).

665. Polek, E., van Oudenhoven, J. P. & Ten Berge, J. M. Attachment styles and demographic factors as predictors of sociocultural and psychological adjustment of Eastern European immigrants in the Netherlands. *International Journal of Psychology* **43**, 919-928 (2008).

666. Polek, E., Wöhrle, J. & Pieter van Oudenhoven, J. The Role of Attachment Styles, Perceived Discrimination, and Cultural Distance in Adjustment of German and Eastern European Immigrants in the

Netherlands. *Cross-Cultural Research* **44**, 60-88 (2010).

667. Ponciano, C., Wang, C. D. & Jin, L. Attachment, acculturative stress, and mental health of Mexican immigrants. *Counselling Psychology Quarterly* **35**, 381-396 (2020).

668. Ponizovsky-Bergelson, Y., Kurman, J. & Roer-Strier, D. Adjustment enhancer or moderator? The role of resilience in postmigration filial responsibility. *Journal of Family Psychology* **29**, 438-446 (2015).

669. Poyrazli, S., Arbona, C., Bullington, R. & Pisecco, S. Adjustment issues of Turkish college students studying in the United States. *College Student Journal* **35**, 52-62 (2001).

670. Poyrazli, S. & Kavanaugh, P. R. Marital status, ethnicity, academic achievement, and adjustment strains: the case of graduate international students. *College Student Journal* **40**, 767+ (2006).

671. Praharso, N. F., Tear, M. J. & Cruwys, T. Stressful life transitions and wellbeing: A comparison of the stress buffering hypothesis and the social identity model of identity change. *Psychiatry research* **247**, 265-275 (2017).

672. Prempeh, L., Thomas, B. & Caldwell, N. Acculturative stress and cognitive appraisals of stress as predictors of cross-cultural adaptation among international students. (ProQuest Dissertations and Theses, 2018).

673. Presbitero, A. Culture shock and reverse culture shock: The moderating role of cultural intelligence in international students' adaptation. *International Journal of Intercultural Relations* **53**, 28-38 (2016).

674. Puck, J. F., Kittler, M. G. & Wright, C. Does it really work? Re-assessing the impact of pre-departure cross-cultural training on expatriate adjustment. *The International Journal of Human Resource Management* **19**, 2182-2197 (2008).

675. Qin, C. & Baruch, Y. The impact of cross-cultural;training for expatriates in a;Chinese firm. *Career Development International* **15**, 296-318 (2010).

676. Qu, D., Chen, C., Kouros, C. D. & Yu, N. X. Congruence and discrepancy in migrant children's and mothers' perceived discrimination: Using response surface analysis to examine the effects on psychological distress. *Applied Psychology: Health and Well-Being* **13**, 602-619 (2021).

677. Rafiq, A., Saleem, S., Bashir, M. & Ali, A. The paradox role of extraversion in the cross-cultural

adjustment process of Asian expatriates. *Psychology research and behavior management* **12**, 179-194 (2019).

678. Rahman, O. & Rollock, D. Acculturation, Competence, and Mental Health Among South Asian Students in the United States. *Journal of Multicultural Counseling and Development* **32**, 130-142 (2004).

679. Ramos, A. K. Precarious work, invisible people, unjust livelihoods: A social ecological model of migrant farmworker health in the Midwest (ProQuest Dissertations and Theses, 2017).

680. Ramos, A. K., McGinley, M. & Carlo, G. The relations of workplace safety, perceived occupational stress, and adjustment among Latino/a immigrant cattle feedyard workers in the United States. *Safety science* **139**, 105262 (2021).

681. Ramos, K. Latino immigrant students: exploring the relationship between migration experience and education outcomes. (University of Oregon, 2015).

682. Rapaport, M., Doucerain, M. M. & Gouin, J. P. Come abroad with me: the role of partner characteristics and couple acculturation gaps on individual psychological adjustment. *Journal of Ethnic and Migration Studies* **49**, 1251-1273 (2021).

683. Rasmussen, A., Cissé, A., Han, Y. & Roubeni, S. Migration factors in West African immigrant parents' perceptions of their children's neighborhood safety. *American journal of community psychology* **61**, 321-331 (2018).

684. Ravasi, C., Salamin, X. & Davoine, E. Cross-cultural adjustment of skilled migrants in a multicultural and multilingual environment: an explorative study of foreign employees and their spouses in the Swiss context. *The International Journal of Human Resource Management* **26**, 1335-1359 (2015).

685. Recker, C., Milfont, T. L. & Ward, C. Un modelo de procesamiento motivacional dual de las conductas de aculturación y resultados de la adaptación. *Universitas Psychologica* **16**, 1-15 (2018).

686. Regev, S. & Slonim-Nevo, V. Sorrow shared is halved? War trauma experienced by others and mental health among Darfuri asylum seekers. *Psychiatry research* **273**, 475-480 (2019).

687. Reid, K. & Berle, D. Parental trajectories of PTSD and child adjustment: Findings from the Building a New Life in Australia study. *American Journal of Orthopsychiatry* **90**, 288-295 (2020).

688. Rekha, P. S. & Vasantha, S. Cross Cultural Competences of Indian it Expatriates Influencing Social Cultural Adaptation in USA. *Indian Journal of Public Health Research & Development* **8**, 327-331 (2017).

689. Ren, H., Harrison, D. A., Shaffer, M. A. & Bhaskar-Shrinivas, P. Beyond adjustment: complex roles of personality and health-related strains in expatriate performance. *European Journal of International Management* **10**, 54-77 (2016).

690. Ren, H., Shaffer, M. A., Harrison, D. A., Fu, C. & Fodchuk, K. M. Reactive Adjustment or Proactive Embedding? Multistudy, Multiwave Evidence for Dual Pathways to Expatriate Retention. *Personnel Psychology* **67**, 203-239 (2014).

691. Renner, A.*, et al.* Predictors of psychological distress in Syrian refugees with posttraumatic stress in Germany. *PLoS One* **16**, e0254406 (2021).

692. Revens, K. E.*, et al.* Social Support and Religiosity as Contributing Factors to Resilience and Mental Wellbeing in Latino Immigrants: A Community-Based Participatory Research Study. *Journal of Immigrant and Minority Health* **23**, 904-916 (2021).

693. Reyna, A. H. The impact of policy change on self-rated mental health among Hispanic and Latino immigrants. (ProQuest Dissertations and Theses, 2021).

694. Riley, A., Varner, A., Ventevogel, P., Taimur Hasan, M. M. & Welton-Mitchell, C. Daily stressors, trauma exposure, and mental health among stateless Rohingya refugees in Bangladesh. *Transcultural psychiatry* **54**, 304-331 (2017).

695. Ritchie, W., Brantley, B. I., Pattie, M., Swanson, B. & Logsdon, J. Expatriate Cultural Antecedents and Outcomes. *Nonprofit Management and Leadership* **25**, 325-342 (2015).

696. Rizkalla, N. & Segal, S. P. Well-Being and Posttraumatic Growth Among Syrian Refugees in Jordan. *Journal of Traumatic Stress* **31**, 213-222 (2018).

697. Robert, G., Martinez, J. M., Garcia, A. M., Benavides, F. G. & Ronda, E. From the boom to the crisis: changes in employment conditions of immigrants in Spain and their effects on mental health. *The European Journal of Public Health* **24**, 404-409 (2014).

698. Robertson, C. L., Savik, K., Mathiason-Moore, M., Mohamed, A. & Hoffman, S. Modeling Psychological

Functioning in Refugees. *Journal of the American Psychiatric Nurses Association* **22**, 225-232 (2016).

699. Rodriguez, G. M. Language and health in the Hispanic|Latino population. (ProQuest Dissertations and Theses, 2015).

700. Roh, S., Lee, Y. S., Kim, Y., Park, S. Y. & Chaudhuri, A. Gender Differences in the Roles of Religious Support and Social Network Support in Reducing Depressive Symptoms Among Older Korean Americans. *Journal of Social Service Research* **41**, 484-497 (2015).

701. Roh, S., Lee, Y. S., Lee, J. H. & Martin, J. I. Typology of religiosity/spirituality in relation to perceived health, depression, and life satisfaction among older Korean immigrants. *Aging Ment Health* **18**, 444-453 (2014).

702. Rohde, D. Latino immigrant stressors, emotional exhaustion, coping resources and work-related outcomes. *North American Journal of Psychology* **20.0**, 121-150 (2018).

703. Roley, M. E.*, et al.* Family cohesion moderates the relationship between acculturative stress and depression in Japanese adolescent temporary residents. *Journal of Immigrant and Minority Health* **16**, 1299-1302 (2014).

704. Rousseau, C., Hassan, G., Moreau, N. & Thombs, B. D. Perceived Discrimination and Its Association With Psychological Distress Among Newly Arrived Immigrants Before and After September 11, 2001. *American Journal of Public Health* **101**, 909-915 (2011).

705. Roy, S. How discrimination, social support, and english proficiency impact depression in south Asian immigrants: The mediators of atherosclerosis in South Asians living in America study. (ProQuest Dissertations and Theses, 2020).

706. Rui, J. R. & Wang, H. Social network sites and international students' cross-cultural adaptation. *Computers in Human Behavior* **49**, 400-411 (2015).

707. Rujipak, V. & Limprasert, S. International Students' Adjustment in Thailand. *ABAC Journal* **36.0**, 34-46 (2016).

708. Ryabichenko, T. A. & Lebedeva, N. M. Assimilation or integration: Similarities and differences between acculturation attitudes of migrants from Central Asia and Russians in Central Russia. *Psychology in*

Russia: State of the Art **9**, 98-111 (2016).

709. Ryu, W. The Effect of Traumatic Experiences of North Korean Adolescent Refugees upon Their Negative Health Perception: Focusing on Multiple Moderating Effect of Problem-Focused versus Social Support-Focused Coping Strategies. *International journal of environmental research and public health* **17**, 1-11 (2020).

710. Saasa, S. & Miller, S. Biopsychosocial Predictors of Depression and Anxiety Symptoms among First-Generation Black African Immigrants. *Social Work in Mental Health* **66**, 329-338 (2021).

711. Safdar, S., Ray-Yol, E., Reif, J. A. & Berger, R. Multidimensional Individual Difference Acculturation (MIDA) model: Syrian refugees' adaptation into Germany. *International Journal of Intercultural Relations* **85**, 156-169 (2021).

712. Saint Arnault, D. & Shimabukuro, S. Floating on Air: Fulfillment and Self-in-Context for Distressed Japanese Women. *Western journal of nursing research* **38**, 572-595 (2016).

713. Salas-Wright, C. P.*, et al.* Trends and mental health correlates of discrimination among Latin American and Asian immigrants in the United States. *Social Psychiatry and Psychiatric Epidemiology* **55**, 477-486 (2020).

714. Salgado, J. F. & Bastida, M. Predicting expatriate effectiveness: The role of personality, cross-cultural adjustment, and organizational support. *International Journal of Selection and Assessment* **25**, 267-275 (2017).

715. Salo, C. D. & Birman, D. Acculturation and Psychological Adjustment of Vietnamese Refugees: An Ecological Acculturation Framework. *American journal of community psychology* **56**, 395-407 (2015).

716. Sam, D. L. Satisfaction with life among international students: An exploratory study. *Social Indicators Research* **53**, 315-337 (2001).

717. Sam, D. L., Tetteh, D. K. & Amponsah, B. Satisfaction with life and psychological symptoms among international students in Ghana and their correlates. *International Journal of Intercultural Relations* **49**, 156-167 (2015).

718. Sambasivan, M., Sadoughi, M. & Esmaeilzadeh, P. Investigating the factors influencing cultural

adjustment and expatriate performance. *International Journal of Productivity and Performance Management* **66**, 1002-1019 (2017).

719. Sánchez-Teruel, D., Robles-Bello, M. A. & Camacho-Conde, J. A. Resilience and the variables that encourage it in young sub-saharan Africans who migrate. *Children and Youth Services Review* **119**(2020).

720. Sangalang, C. C*., et al.* Trauma, Post-Migration Stress, and Mental Health: A Comparative Analysis of Refugees and Immigrants in the United States. *Journal of Immigrant and Minority Health* **21**, 909-919 (2019).

721. Sari, H. Y., Gelbal, S. & Halil, S. Factors Affecting Academic Self-Efficacy of Syrian Refugee Students: A Path Analysis Model. *International Journal of Assessment Tools in Education* **7.0**, 266-279 (2020).

722. Sarwar, F., Panatik, S. A. & Jameel, H. T. Does fear of terrorism influence psychological adjustment of academic sojourners in Pakistan? Role of state negative affect and emotional support. *International Journal of Intercultural Relations* **75**, 34-47 (2020).

723. Saw, Y. E., Tan, E. Y., Buvanaswari, P., Doshi, K. & Liu, J. C. Mental health of international migrant workers amidst large-scale dormitory outbreaks of COVID-19: A population survey in Singapore. *Journal of migration and health* **4**, 100062 (2021).

724. Schaafsma, J. Discrimination and subjective well-being: The moderating roles of identification with the heritage group and the host majority group. *European Journal of Social Psychology* **41**, 786-795 (2011).

725. Scherer, N*., et al.* Prevalence of common mental disorders among Syrian refugee children and adolescents in Sultanbeyli district, Istanbul: results of a population-based survey. *Epidemiology and psychiatric sciences* **29**, e192 (2020).

726. Schmitt, M. T., Spears, R. & Branscombe, N. R. Constructing a minority group identity out of shared rejection: the case of international students. *European Journal of Social Psychology* **33**, 1-12 (2003).

727. Schunck, R., Reiss, K. & Razum, O. Pathways between perceived discrimination and health among immigrants: evidence from a large national panel survey in Germany. *Ethnicity & health* **20**, 493-510 (2015).

728. Schuster, T., Ambrosius, J. & Bader, B. Mentoring in international assignments: a personality traits

perspective. *Employee Relations* **39**, 1100-1130 (2017).

729. Schwartz, S. J., *et al.* Cultural stress and psychological symptoms in recent Venezuelan immigrants to the United States and Colombia. *International Journal of Intercultural Relations* **67**, 25-34 (2018).

730. Schwartz, S. J., *et al.* Perceived context of reception among recent Hispanic immigrants: conceptualization, instrument development, and preliminary validation. *Cultural Diversity and Ethnic Minority Psychology* **20**, 1-15 (2014).

731. Searle, W. & Ward, C. The prediction of psychological and sociocultural adjustment during cross-cultural transitions. *International Journal of Intercultural Relations* **14**, 449-464 (1990).

732. Seglem, K. B., Oppedal, B. & Roysamb, E. Daily hassles and coping dispositions as predictors of psychological adjustment. *International Journal of Behavioral Development* **38**, 293-303 (2014).

733. Selmer, J. The Chinese connection? Adjustment of Western vs. overseas Chinese expatriate managers in China. *Journal of Business Research* **55**, 41-50 (2002).

734. Selmer, J. Cultural novelty and adjustment: Western business expatriates in China. *The International Journal of Human Resource Management* **17**, 1209-1222 (2006).

735. Selmer, J. & Lauring, J. Cultural similarity and adjustment of expatriate academics. *International Journal of Intercultural Relations* **33**, 429-436 (2009).

736. Selmer, J., Lauring, J., Normann, J. & Kubovcikova, A. Context matters: Acculturation and work-related outcomes of self-initiated expatriates employed by foreign vs. local organizations. *International Journal of Intercultural Relations* **49**, 251-264 (2015).

737. Sengoelge, M., Nissen, A. & Solberg, O. Post-Migration Stressors and Health-Related Quality of Life in Refugees from Syria Resettled in Sweden. *International journal of environmental research and public health* **19**(2022).

738. Sengoelge, M., Solberg, O., Nissen, A. & Saboonchi, F. Exploring Social and Financial Hardship, Mental Health Problems and the Role of Social Support in Asylum Seekers Using Structural Equation Modelling. *International journal of environmental research and public health* **17**, 1-14 (2020).

739. Seo, H., Harn, R. W., Ebrahim, H. & Aldana, J. International students' social media use and social

adjustment. *First Monday* **21.0**(2016).

740. Seo, Y. J., *et al.* The Mediating Role of Korean Immigrant Mothers' Psychological Well-Being in the Associations between Social Support and Authoritarian Parenting Style. *Journal of Child and Family Studies* **27**, 979-989 (2017).

741. Serafica, R., Lekhak, N. & Bhatta, T. Acculturation, acculturative stress and resilience among older immigrants in United States. *International Nursing Review* **66**, 442-448 (2019).

742. Setti, I., Sommovigo, V. & Argentero, P. Enhancing expatriates' assignments success: the relationships between cultural intelligence, cross-cultural adaptation and performance. *Current Psychology* **41**, 4291-4311 (2020).

743. Sevillano, V., Basabe, N., Bobowik, M. & Aierdi, X. Health-related quality of life, ethnicity and perceived discrimination among immigrants and natives in Spain. *Ethnicity & health* **19**, 178-197 (2014).

744. Shafaei, A. & Razak, N. A. What matters most: importance-performance matrix analysis of the factors influencing international postgraduate students' psychological and sociocultural adaptations. *Quality & Quantity* **52**, 37-56 (2016).

745. Shafaei, A. & Razak, N. A. International Postgraduate Students' Cross-Cultural Adaptation in Malaysia: Antecedents and Outcomes. *Research in Higher Education* **57**, 739-767 (2016).

746. Shaffer, M. A. & Harrison, D. A. Expatriates' psychological withdrawal from international assignments: Work, nonwork, and family influences. *Personnel Psychology* **51**, 87-118 (1998).

747. Shaffer, M. A. & Harrison, D. A. Forgotten partners of international assignments: development and test of a model of spouse adjustment. *Journal of Applied Psychology* **86**, 238-254 (2001).

748. Shaffer, M. A., Harrison, D. A. & Gilley, K. M. Dimensions, Determinants, and Differences in the Expatriate Adjustment Process. *Journal of International Business Studies* **30**, 557-581 (1999).

749. Shaffner, E. A. H. An examination of relational health, belonging, and self-compassion in Chinese international students. University of Denver (ProQuest Dissertations and Theses, 2020).

750. Shah, D., de Oliveira, R. T., Barker, M., Moeller, M. & Nguyen, T. Expatriate family adjustment: How organisational support on international assignments matters. *Journal of International Management*

28(2022).

751. Shaw, S. A., Pillai, V. & Ward, K. P. Assessing mental health and service needs among refugees in Malaysia. *International Journal of Social Welfare* **28**, 44-52 (2018).

752. Sheng, L., Dai, J. & Lei, J. The impacts of academic adaptation on psychological and sociocultural adaptation among international students in China: The moderating role of friendship. *International Journal of Intercultural Relations* **89**, 79-89 (2022).

753. Shin, G. & Lee, S. J. Mental health and PTSD in female North Korean refugees. *Health Care for Women International* **36**, 409-423 (2015).

754. Shin, H. S., Han, H. R. & Kim, M. T. Predictors of psychological well-being amongst Korean immigrants to the United States: a structured interview survey. *International journal of nursing studies* **44**, 415-426 (2007).

755. Shin, J. E., Choi, J. S., Choi, S. H. & Yoo, S. Y. The Effect of Postmigration Factors on Quality of Life among North Korean Refugees Living in South Korea. *International journal of environmental research and public health* **18**(2021).

756. Shu, F., Ahmed, S. F., Pickett, M. L., Ayman, R. & McAbee, S. T. Social support perceptions, network characteristics, and international student adjustment. *International Journal of Intercultural Relations* **74**, 136-148 (2020).

757. Shueh, T.-A. Perceived justice, ethnic identity, and international students' sociocultural adaptation. (University of Florida, 2007).

758. Shupe, E. I. Clashing Cultures: A Model of International Student Conflict. *Journal of Cross-Cultural Psychology* **38**, 750-771 (2007).

759. Sierau, S., Schneider, E., Nesterko, Y. & Glaesmer, H. Alone, but protected? Effects of social support on mental health of unaccompanied refugee minors. *European child & adolescent psychiatry* **28**, 769-780 (2019).

760. Silbiger, A., Barnes, B. R., Berger, R. & Renwick, D. W. S. The role of regulatory focus and its influence on the cultural distance – Adjustment relationship for expatriate managers. *Journal of Business Research*

122, 398-410 (2021).

761. Sim, A., Bowes, L. & Gardner, F. Modeling the effects of war exposure and daily stressors on maternal mental health, parenting, and child psychosocial adjustment: a cross-sectional study with Syrian refugees in Lebanon. *Global Mental Health* **5**, e40 (2018).

762. Singh, A. Investigating the sense of belonging for international students through a predictive model. (ProQuest Dissertations and Theses, 2018).

763. Singh, D. Acculturative stress and self-esteem: A study examining the relationship between acculturative stress and perceived self-esteem among immigrants in the United States. (ProQuest Dissertations and Theses, 2017).

764. Singh, S., McBride, K. & Kak, V. Role of Social Support in Examining Acculturative Stress and Psychological Distress Among Asian American Immigrants and Three Sub-groups: Results from NLAAS. *Journal of Immigrant and Minority Health* **17**, 1597-1606 (2015).

765. Singh, S., Schulz, A. J., Neighbors, H. W. & Griffith, D. M. Interactive Effect of Immigration-Related Factors with Legal and Discrimination Acculturative Stress in Predicting Depression Among Asian American Immigrants. *Community mental health journal* **53**, 638-646 (2017).

766. Sirlopú, D. & Renger, D. Social recognition matters: Consequences for school participation and life satisfaction among immigrant students. *Journal of Community & Applied Social Psychology* **30**, 561-575 (2020).

767. Sleijpen, M., Haagen, J., Mooren, T. & Kleber, R. J. Growing from experience: an exploratory study of posttraumatic growth in adolescent refugees. *European Journal of Psychotraumatology* **7**, 28698 (2016).

768. Sokro, E., Pillay, S. & Bednall, T. The effects of perceived organisational support on expatriate adjustment, assignment completion and job satisfaction. *International Journal of Cross Cultural Management* **21**, 452-473 (2021).

769. Solberg, O., *et al.* Health-related quality of life in refugee minors from Syria, Iraq and Afghanistan resettled in Sweden: a nation-wide, cross-sectional study. *Social Psychiatry and Psychiatric Epidemiology* **57**, 255-266 (2022).

770. Solberg, O., Sengoelge, M., Nissen, A. & Saboonchi, F. Coping in Limbo? The Moderating Role of Coping Strategies in the Relationship between Post-Migration Stress and Well-Being during the Asylum-Seeking Process. *International journal of environmental research and public health* **18**, 1-16 (2021).

771. Solberg, O., Vaez, M., Johnson-Singh, C. M. & Saboonchi, F. Asylum-seekers' psychosocial situation: A diathesis for post-migratory stress and mental health disorders? *Journal of psychosomatic research* **130**, 109914 (2020).

772. Son, H. Established multicultural families' work and life: The impact of employment and perceived Korean husbands' practical support on migrant wives' life satisfaction. (ProQuest Dissertations and Theses, 2018).

773. Sonderegger, R., Barrett, P. M. & Creed, P.A. Models of Cultural Adjustment for Child and Adolescent Migrants to Australia: Internal Process and Situational Factors. *Journal of Child and Family Studies* **13**, 357-371 (2004).

774. Song, H., Zhao, S., Zhao, W. & Han, H. Career Development Support, Job Adaptation, and Withdrawal Intention of Expatriates: A Multilevel Analysis of Environmental Factors. *International journal of environmental research and public health* **16**(2019).

775. Song, H. R. The role of multicultural social network in the relationship between the role of multicultural social network in the relationship between acculturative stress and depression among Korean immigrants in the U.S. (ProQuest Dissertations and Theses, 2021).

776. Spaas, C., *et al.* Mental Health of Refugee and Non-refugee Migrant Young People in European Secondary Education: The Role of Family Separation, Daily Material Stress and Perceived Discrimination in Resettlement. *Journal of youth and adolescence* **51**, 848-870 (2022).

777. Stadnik, N. The quality of life and well-being among older immigrants of Eastern European origin. (ProQuest Dissertations and Theses, 2022).

778. Stafford, A. M., Tsumura, H. & Pan, W. Race/Ethnicity, Parental Support, and Youth Depressive Symptoms: A Moderated Longitudinal Mediation Analysis. *Journal of youth and adolescence* **50**, 1319-1332 (2021).

779. Stahl, G. K. & Caligiuri, P. The effectiveness of expatriate coping strategies: the moderating role of cultural distance, position level, and time on the international assignment. *Journal of Applied Psychology* **90**, 603-615 (2005).

780. Steel, J. L., Dunlavy, A. C., Harding, C. E. & Theorell, T. The Psychological Consequences of Pre-Emigration Trauma and Post-Migration Stress in Refugees and Immigrants from Africa. *Journal of Immigrant and Minority Health* **19**, 523-532 (2017).

781. Steel, K. C., Fernandez-Esquer, M. E., Atkinson, J. S. & Taylor, W. C. Exploring relationships among social integration, social isolation, self-rated health, and demographics among Latino day laborers. *Ethnicity & health* **23**, 425-441 (2018).

782. Stempel, C., *et al.* Gendered Sources of Distress and Resilience among Afghan Refugees in Northern California: A Cross-Sectional Study. *International journal of environmental research and public health* **14**(2016).

783. Stierle, C., van Dick, R. & Wagner, U. Success or Failure? Personality, Family, and Intercultural Orientation as Determinants of Expatriate Managers' Success. *Zeitschrift für Sozialpsychologie* (2002).

784. Stoermer, S., Haslberger, A., Froese, F. J. & Kraeh, A. L. Person-Environment Fit and Expatriate Job Satisfaction. *Thunderbird International Business Review* **60**, 851-860 (2018).

785. Straiton, M., Grant, J. F., Winefield, H. R. & Taylor, A. Mental health in immigrant men and women in Australia: the North West Adelaide Health Study. *BMC Public Health* **14**, 1111 (2014).

786. Straiton, M. L., Aambo, A. K. & Johansen, R. Perceived discrimination, health and mental health among immigrants in Norway: the role of moderating factors. *BMC Public Health* **19**, 325 (2019).

787. Stroh, L. K., Dennis, L. E. & Cramer, T. C. Predictors of expatriate adjustment. *The International Journal of Organizational Analysis* **2**, 176-192 (1994).

788. Stroppa, C. & Spieß, E. International assignments: The role of social support and personal initiative. *International Journal of Intercultural Relations* **35**, 234-245 (2011).

789. Stuart, J. & Nowosad, J. The Influence of Premigration Trauma Exposure and Early Postmigration Stressors on Changes in Mental Health Over Time Among Refugees in Australia. *Journal of Traumatic*

Stress **33**, 917-927 (2020).

790. Su, S., Lin, X. & McElwain, A. Parenting, loneliness, and stress in Chinese international students: do parents still matter from thousands of miles away? *Journal of Family Studies* **29**, 255-268 (2021).

791. Su, T. Does family cohesion moderate the relationship between acculturative stress and depression among Asian American immigrants? *Asian American Journal of Psychology* **13**, 141-148 (2022).

792. Su, Z.*, et al.* Chinese International Students in the United States: The Interplay of Students' Acculturative Stress, Academic Standing, and Quality of Life. *Frontiers in Psychology* **12**, 625863 (2021).

793. Subedi, A. Coping strategies and psychological well-being among Bhutanese refugees resettled in Ottawa, Canada. (ProQuest Dissertations and Theses, 2016).

794. Subedi, R. P. Healthy immigrants? Healthy workers? High-skilled immigrants working in low-skilled jobs in Ottawa, Canada. (ProQuest Dissertations and Theses, 2016).

795. Suh, H.*, et al.* Measuring acculturative stress with the SAFE: Evidence for longitudinal measurement invariance and associations with life satisfaction. *Personality and Individual Differences* **89**, 217-222 (2016).

796. Suh, H. N., Flores, L. Y. & Wang, K. T. Perceived Discrimination, Ethnic Identity, and Mental Distress Among Asian International Students in Korea. *Journal of Cross-Cultural Psychology* **50**, 991-1007 (2019).

797. Sumer, S. International Students' Psychological and Sociocultural Adaptation in the United States. *Doctoral dissertation* (2009).

798. Sümer, S., Poyrazli, S. & Grahame, K. Predictors of Depression and Anxiety Among International Students. *Journal of Counseling & Development* **86**, 429-437 (2008).

799. Sun, K. Parenting in Chinese immigrant mothers: The influences of Chinese identity, cultural and parenting cognitions, grandparent support and child temperament. (ProQuest Dissertations and Theses, 2019).

800. Sun, K. S.*, et al.* Associations between demographic factors and psychological distress among Chinese residents in Hong Kong: beyond socioeconomic classes. *Psychology, Health & Medicine* **25**, 1049-1061

(2020).

801. Sun, X., Hall, G. C. N., DeGarmo, D. S., Chain, J. & Fong, M. C. A Longitudinal Investigation of Discrimination and Mental Health in Chinese International Students: The Role of Social Connectedness. *Journal of Cross-Cultural Psychology* **52**, 61-77 (2020).

802. Supangco, V. & Mayrhofer, W. Determinants of work role transition outcomes of Filipinos in Singapore. *Journal of Global Mobility* **2**, 317-342 (2014).

803. Swagler, M. A. & Ellis, M. V. Crossing the distance: Adjustment of Taiwanese Graduate Students in the United States. *Journal of Counseling Psychology* **50**, 420-437 (2003).

804. Swami, V. Predictors of sociocultural adjustment among sojourning Malaysian students in Britain. *International Journal of Psychology* **44**, 266-273 (2009).

805. Swami, V., Arteche, A., Chamorro-Premuzic, T. & Furnham, A. Sociocultural adjustment among sojourning Malaysian students in Britain: a replication and path analytic extension. *Social Psychiatry and Psychiatric Epidemiology* **45**, 57-65 (2010).

806. Szabó, Á. Envisioning positive future selves: Perceptions of the future self and psychological adaptation in recent migrants. *Self and Identity* **21**, 877-890 (2021).

807. Szabo, A*., et al.* Is the Utility of Secondary Coping a Function of Ethnicity or the Context of Reception? A Longitudinal Study Across Western and Eastern Cultures. *Journal of Cross-Cultural Psychology* **48**, 1230-1246 (2017).

808. Szabo, A., Klokgieters, S. S., Kok, A. A. L., van Tilburg, T. G. & Huisman, M. Psychological Resilience in the Context of Disability: A Study With Turkish and Moroccan Young-Old Immigrants Living in the Netherlands. *Gerontologist* **60**, 259-269 (2020).

809. Szabó, Á., Papp, Z. Z. & Luu, L. A. N. Social contact configurations of international students at school and outside of school: Implications for acculturation orientations and psychological adjustment. *International Journal of Intercultural Relations* **77**, 69-82 (2020).

810. Szabo, A. & Ward, C. Identity development during cultural transition: The role of social-cognitive identity processes. *International Journal of Intercultural Relations* **46**, 13-25 (2015).

811. Szabo, A., Ward, C. & Jose, P. E. Uprooting stress, coping, and anxiety: A longitudinal study of international students. *International Journal of Stress Management* **23**, 190-208 (2016).

812. Tadik, Y. C. Longitudinal associations between marital quality and maternal psychological well-being: The mediating role of coparenting in US-Born and non-US-born parents. (ProQuest Dissertations and Theses, 2020).

813. Takatsuka, M. & Yimcharoen, P. The Impact of Perceived Organizational Support and Cross-Cultural Adjustment on Job Burnout and Job Satisfaction: A Case of Japanese Expatriates, Thailand. in *2021 6th International Conference on Business and Industrial Research (ICBIR)* 53-58 (2021).

814. Takenoshita, H. Social Capital and Mental Health among Brazilian Immigrants in Japan. *International Journal of Japanese Sociology* **24**, 48-64 (2015).

815. Takeuchi, R., Lepak, D. P., Marinova, S. V. & Yun, S. Nonlinear influences of stressors on general adjustment: the case of Japanese expatriates and their spouses. *Journal of International Business Studies* **38**, 928-943 (2007).

816. Takeuchi, R., Wang, M., Marinova, S. V. & Yao, X. Role of Domain-Specific Facets of Perceived Organizational Support During Expatriation and Implications for Performance. *Organization Science* **20**, 621-634 (2009).

817. Takeuchi, R., Yun, S. & Russell, J. E. A. Antecedents and consequences of the perceived adjustment of Japanese expatriates in the USA. *The International Journal of Human Resource Management* **13**, 1224-1244 (2002).

818. Takeuchi, R., Yun, S. & Tesluk, P. An examination of crossover and spillover effects of spousal and expatriate cross-cultural adjustment. *The Journal of Applied Psychology* **87**, 655-666 (2002).

819. Taliaferro, L. A., Muehlenkamp, J. J. & Jeevanba, S. B. Factors associated with emotional distress and suicide ideation among international college students. *Journal of American college health* **68**, 565-569 (2020).

820. Tanova, C. & Ajayi, O. International faculty member sociocultural adjustment and intention to stay: Evidence from North Cyprus. *Asian Academy of Management Journal* **21.0**, 47-72 (2016).

821. Tarsitani, L.*, et al.* Somatization and traumatic events in asylum seekers and refugees resettled in Italy. *Journal of Psychopathology* **26.0**, 41-45 (2020).

822. Tartakovsky, E., Patrakov, E. & Nikulina, M. Is emigration worth the trouble? Satisfaction with life, group identifications, perceived discrimination, and socio-economic status of immigrants and stayers. *International Journal of Intercultural Relations* **80**, 195-205 (2021).

823. Tartakovsky, E. & Saranga, M. Eritrean Asylum Seekers in Israel: Traumatic Experience, Social Contacts with Eritreans and Israelis, Psychological Well-Being, and Sociocultural Adaptation. *Journal of Immigrant & Refugee Studies*, 1-14 (2022).

824. Tartakovsky, E. & Walsh, S. D. Factors affecting the psychological well-being of immigrants: The role of group self-appraisal, social contacts, and perceived ethnic density. *Cultural Diversity and Ethnic Minority Psychology* **26**, 592-603 (2020).

825. Tatarko, A., Berry, J. W. & Choi, K. Social capital, acculturation attitudes, and sociocultural adaptation of migrants from central Asian republics and South Korea in Russia. *Asian Journal of Social Psychology* **23**, 302-312 (2020).

826. Taušová, J., Bender, M., Dimitrova, R. & van de Vijver, F. The role of perceived cultural distance, personal growth initiative, language proficiencies, and tridimensional acculturation orientations for psychological adjustment among international students. *International Journal of Intercultural Relations* **69**, 11-23 (2019).

827. Taylor, Z. E. & Ruiz, Y. Contextual stressors and the mental health outcomes of Latino children in rural migrant-farmworker families in the midwest. *Journal of Rural Mental Health* **41**, 284-298 (2017).

828. Tegegne, M. A. Social capital and immigrant integration: The role of social capital in labor market and health outcomes. (ProQuest Dissertations and Theses, 2016).

829. Tegegne, M. A. Linguistic Integration and Immigrant Health: The Longitudinal Effects of Interethnic Social Capital. *Journal of Health and Social Behavior* **59**, 215-230 (2018).

830. Teixeira, A. F. & Dias, S. F. Labor market integration, immigration experience, and psychological distress in a multi-ethnic sample of immigrants residing in Portugal. *Ethnicity & health* **23**, 81-96 (2018).

831. Ten Kate, R. L. F., Bilecen, B. & Steverink, N. A Closer Look at Loneliness: Why Do First-Generation Migrants Feel More Lonely Than Their Native Dutch Counterparts? *Gerontologist* **60**, 291-301 (2020).

832. Terry, D. J., Pelly, R. N., Lalonde, R. N. & Smith, J. R. Predictors of Cultural Adjustment: Intergroup Status Relations and Boundary Permeability. *Group Processes and Intergroup Relations* **9**, 249-264 (2006).

833. Thibeault, M. A., Mendez, J. L., Nelson-Gray, R. O. & Stein, G. L. Impact of trauma exposure and acculturative stress on internalizing symptoms for recently arrived migrant-origin youth: Results from a community-based partnership. *Journal of Community Psychology* **45**, 984-998 (2017).

834. Thomas, F. & Sumathi, G. N. Acculturative stress and mental health among international students: An empirical evidence. *International Journal of Mechanical Engineering and Technology* **9.0**, 780-788 (2018).

835. Thomson, G., Rosenthal, D. & Russell, J. Cultural stress among international students at an Australian university. *Australian International Education Conference 2006* (2006).

836. Thornberry, N. R. Counseling and expatriate adjustment. (ProQuest Dissertations and Theses, 2015).

837. Tingvold, L.*, et al.* Predictors of acculturative hassles among Vietnamese refugees in Norway: Results from a long-term longitudinal study. *Transcultural psychiatry* **52**, 700-714 (2015).

838. Tip, L. K., Brown, R., Morrice, L., Collyer, M. & Easterbrook, M. J. Improving Refugee Well-Being With Better Language Skills and More Intergroup Contact. *Social Psychological and Personality Science* **10**, 144-151 (2018).

839. Tippens, J. A.*, et al.* Psychological distress prevalence and associated stressors and supports among urban-displaced Congolese adults in Kenya. *American Journal of Orthopsychiatry* **91**, 626-634 (2021).

840. Tippens, J. A.*, et al.* Emotional support and mental health among Somali men in a rural Midwestern town. *Journal of Rural Mental Health* **44**, 170 (2020).

841. Toh, S. M. *Host country nationals to the rescue: A social categorization approach to expatriate adjustment*, (Texas A&M University, 2003).

842. Tonsing, K. N. Predictors of psychological adaptation of South Asian immigrants in Hong Kong.

International Journal of Intercultural Relations **37**, 238-248 (2013).

843. Tonsing, K. N. Exploratory study of personal wellbeing of Burmese refugees in the United States. *Journal of Ethnic & Cultural Diversity in Social Work* **31**, 329-340 (2020).

844. Tonsing, K. N. & Vungkhanching, M. The relationship between postmigration living difficulties, social support, and psychological distress of Burmese refugees in the United States. *Asian American Journal of Psychology* **11**, 179-186 (2020).

845. Torres, C. V., *et al.* Refugees in Brazil: An investigation of Syrian refugees' psychological experiences. *International Journal of Psychology* **57**, 466-474 (2022).

846. Toyokawa, T. & Toyokawa, N. Extracurricular activities and the adjustment of Asian international students: A study of Japanese students. *International Journal of Intercultural Relations* **26**, 363-379 (2002).

847. Tran, T. V., Wang, K., Bekteshi, V. & Vatcher, R. Marriage, race/ethnicity, and psychological distress among foreign-born Chinese, Korean, Mexican, and Vietnamese Americans. *Social Work in Mental Health* **14**, 245-259 (2015).

848. Trice, A. G. Mixing It Up: International Graduate Students' Social Interactions With American Students. *Journal of College Student Development* **45**, 671-687 (2004).

849. Tsacoumangos, A. Acculturation, social support, and overall well-being in the international student population. (ProQuest Dissertations and Theses, 2018).

850. Tsang, E. W. K. Adjustment of mainland Chinese academics and students to Singapore. *International Journal of Intercultural Relations* **25**, 347-372 (2001).

851. Tsegaye, W. K., Su, Q. & Malik, M. Expatriate cultural values alignment: The mediating effect of cross-cultural adjustment level on innovative behaviour. *Creativity and Innovation Management* **28**, 218-229 (2019).

852. Tso, L. & Shukla, M. Experiences of Tibetan Refugees in India during the COVID-19 Pandemic. *Refuge: Canada's Journal on Refugees* **38**, 43-61 (2022).

853. Tu, C. K., Chen, C. S. & Lam, K. C. Happy expatriates have higher cross-cultural adjustment and

retention? in *Proceedings of the 10th International Conference on E-Education, E-Business, E-Management and E-Learning* 368-373 (2019).

854. Turner, S. L. The effect of cross-cultural training on adjustment and job performance: Examining the role of supervisor skill-building and individual differences. (Rice University, 2007).

855. Uen, J. F., Teng, S. K., Wu, L. C. & Tsao, S. A. The antecedents and consequences of cultural intelligence: An exploratory study of Taiwanese expatriates. in *2018 5th International Conference on Business and Industrial Research (ICBIR)* 481-485 (2018).

856. Um, M. Y., Chi, I., Kim, H. J., Palinkas, L. A. & Kim, J. Y. Correlates of depressive symptoms among North Korean refugees adapting to South Korean society: the moderating role of perceived discrimination. *Social Science & Medicine* **131**, 107-113 (2015).

857. Upvall, M. J. A Model of Uprooting for International Students. *Western Journal of Nursing Research* **12**, 95-107 (1990).

858. Urzua, A., Cabrera, C., Carvajal, C. C. & Caqueo-Urizar, A. The mediating role of self-esteem on the relationship between perceived discrimination and mental health in South American immigrants in Chile. *Psychiatry research* **271**, 187-194 (2019).

859. Urzua, A*., et al.* The mediating effect of self-esteem on the relationship between perceived discrimination and psychological well-being in immigrants. *PLoS One* **13**, e0198413 (2018).

860. Urzua, A., Henriquez, D. & Caqueo-Urizar, A. Affects as Mediators of the Negative Effects of Discrimination on Psychological Well-Being in the Migrant Population. *Frontiers in Psychology* **11**, 602537 (2020).

861. Urzua, A., Henriquez, D., Caqueo-Urizar, A. & Smith-Castro, V. Validation of the brief scale for the evaluation of acculturation stress in migrant population (EBEA). *Psicologia: Reflexão e Crítica* **34**, 3 (2021).

862. Usunier, J.-C. Oral pleasure and expatriate satisfaction: an empirical approach. *International Business Review* **7**, 89-110 (1998).

863. Uygun, E. The relation between Syrians' quality of life, depression and anxiety levels and economic

conditions: a cross-sectional study at an adult refugee mental health clinic in Turkey. *Anatolian Journal of Psychiatry/Anadolu Psikiyatri Dergisi* **21**(2020).

864. Uysal, B., *et al.* Psychological problems and resilience among Syrian adolescents exposed to war. *European Journal of Trauma & Dissociation* **6**(2022).

865. van der Bank, M. & Rothmann, S. Correlates of expatriates' cross-cultural adjustment. *Management Dynamics* **15**, 29-39 (2006).

866. van Der Zee, K. I., Ali, A. J. & Haaksma, I. Determinants of effective coping with cultural transition among expatriate children and adolescents. *Anxiety, Stress, & Coping* **20**, 25-45 (2007).

867. van der Zee, K. I., Ali, A. J. & Salomé, E. Role interference and subjective well-being among expatriate families. *European Journal of Work and Organizational Psychology* **14**, 239-262 (2005).

868. van Erp, K. J. P. M., van der Zee, K. I., Giebels, E. & van Duijn, M. A. J. Lean on me: The importance of one's own and partner's intercultural personality for expatriate's and expatriate spouse's successful adjustment abroad. *European Journal of Work and Organizational Psychology* **23**, 706-728 (2014).

869. van Heemstra, H. E., Scholte, W. F., Nickerson, A. & Boelen, P. A. Can Circumstances Be Softened? Self-Efficacy, Post-Migratory Stressors, and Mental Health among Refugees. *International journal of environmental research and public health* **18**, 1-9 (2021).

870. van Vianen, A. E. M., De Pater, I. E., Kristof-Brown, A. L. & Johnson, E. C. Fitting in: Surface- and Deep-Level Cultural Differences and Expatriates' Adjustment. *Academy of Management Journal* **47**, 697-709 (2004).

871. VanderWielen, J. J. *Cognitive appraisal, coping, and the psychological and sociocultural adjustment of expatriates*, (California School of Professional Psychology-San Diego, 2001).

872. Vaquera, E. & Aranda, E. Moving Up and Down the Ladder: Perceived Social Mobility and Emotional Dispositions Among South Florida's Immigrants. *Sociological Forum* **32**, 793-815 (2017).

873. Vargas, K. The relationship of acculturation, ethnic identity and social support on the psychological well-being of Latino immigrants. (ProQuest Dissertations and Theses, 2017).

874. Varma, A., Mathew, J., Wang, C. H., Budhwar, P. & Katou, A. Indian Nurses in the United Kingdom: A

Two-Phase Study of the Expatriate-Host Country National Relationship. *European Management Review* **18**, 329-341 (2020).

875. Vartevan, A. The correlation between life satisfaction, subjective wellbeing, acculturation, and acculturative stress among first-generation Armenian immigrants in the United States. (ProQuest Dissertations and Theses, 2018).

876. Vazquez, V.*, et al.* Depressive symptoms among recent Latinx immigrants in South Florida: The role of premigration trauma and stress, postimmigration stress, and gender. *Journal of Traumatic Stress* **35**, 533-545 (2022).

877. Velarde Pierce, S., Haro, A. Y., Ayón, C. & Enriquez, L. E. Evaluating the Effect of Legal Vulnerabilities and Social Support on the Mental Health of Undocumented College Students. *Journal of Latinos and Education* **20**, 246-259 (2021).

878. Venta, A.*, et al.* Contribution of schools to mental health and resilience in recently immigrated youth. *School Psychology* **34**, 138-147 (2019).

879. Vervliet, M., Lammertyn, J., Broekaert, E. & Derluyn, I. Longitudinal follow-up of the mental health of unaccompanied refugee minors. *European child & adolescent psychiatry* **23**, 337-346 (2014).

880. Vijayakumar, P. B. & Cunningham, C. J. L. Impact of spousal work restrictions on expatriates' work life and overall life satisfaction. *International Journal of Psychology* **55**, 959-963 (2020).

881. Vinokurov, A. & Trickett, E. J. Ethnic Clusters in Public Housing and Independent Living of Elderly Immigrants from the Former Soviet Union. *Journal of cross-cultural gerontology* **30**, 353-376 (2015).

882. Vinokurov, A., Trickett, E. J. & Birman, D. The Effect of Ethnic Community on Acculturation and Cultural Adaptation: the Case of Russian-Speaking Older Adults. *Journal of International Migration and Integration* **21**, 1057-1081 (2019).

883. Viswanathan, R., Mohammed, M., Sarath, L. N. & Parveen, J. Impact of Emotional Intelligence, Cultural Intelligence, Perceived Organizational Supporting Practices and Perceived Family Support on Cross Cultural Adjustment of Indian it Expatriates Working in the United States of America. *International Journal of Recent Technology and Engineering* **8**, 368-377 (2019).

884. Viswanathan, R., Moiz, M., Sarath, L. N. & Parveen, J. Does Emotional Intelligence Pave way for Psychological Well Being and Enhancing Work Performance. *International Journal of Recent Technology and Engineering* **8**, 663-672 (2019).

885. Vohra, N. Life satisfaction of Indian immigrants in Canada. (University of Manitoba, 1995).

886. Vohra, N. & Adair, J. Life satisfaction of Indian immigrants in Canada. *Psychology and Developing Societies* **12**, 109-138 (2000).

887. Volpone, S. D., Marquardt, D. J., Casper, W. J. & Avery, D. R. Minimizing cross-cultural maladaptation: How minority status facilitates change in international acculturation. *Journal of Applied Psychology* **103**, 249-269 (2018).

888. Vora, K. Acculturative Stress and Adjustment Among Adult International Students in Goa. *The Journal of Continuing Higher Education* **71**, 1-23 (2021).

889. Vulić-Prtorić, A. & Oetjen, N. Adaptation and acculturation of international students in croatia [Prilagodba i akulturacija internacionalnih studenata u hrvatskoj]. *Collegium Antropologicum* **41.0**, 335-343 (2017).

890. Waldman, Z. C.*, et al.* Sleep and Economic Status Are Linked to Daily Life Stress in African-Born Blacks Living in America. *International journal of environmental research and public health* **19**(2022).

891. Wang, A. Chinese international students' health and well-being in UK universities. (ProQuest Dissertations and Theses, 2017).

892. Wang, C. D.*, et al.* Adult attachment, acculturation, acculturative stress, and psychological distress of first-generation latinx immigrants. *Counselling Psychology Quarterly* **35**, 634-651 (2021).

893. Wang, J., Hong, J. Z. & Pi, Z. L. Cross-Cultural Adaptation: The Impact of Online Social Support and the Role of Gender. *Social Behavior and Personality: an international journal* **43**, 111-121 (2015).

894. Wang, K. T.*, et al.* Profiles of Acculturative Adjustment Patterns Among Chinese International Students. *Journal of Counseling Psychology* **59**, 424-436 (2012).

895. Wang, K. T., Heppner, P. P., Wang, L. & Zhu, F. Cultural Intelligence Trajectories in New International Students: Implications for the Development of Cross-Cultural Competence. *International Perspectives in*

Psychology **4**, 51-65 (2015).

896. Wang, K. T., Wei, M. & Chen, H.-H. Social Factors in Cross-National Adjustment. *The Counseling Psychologist* **43**, 272-298 (2015).

897. Wang, L., Wang, K. T., Heppner, P. P. & Chuang, C.-C. Cross-national cultural competency among Taiwanese international students. *Journal of Diversity in Higher Education* **10**, 271-287 (2017).

898. Wang, M. & Takeuchi, R. The Role of Goal Orientation During Expatriation: A Cross-Sectional and Longitudinal Investigation. *The Journal of Applied Psychology* **92**, 1437-1445 (2007).

899. Wang, X. Expatriate social support network, psychological well-being, and performance: A theoretical examination and an empirical test. (2001).

900. Wang, X. & Kanungo, R. N. Nationality, social network and psychological well-being: expatriates in China. *The International Journal of Human Resource Management* **15**, 775-793 (2004).

901. Wang, X. & Nayir, D. Z. How and when is social networking important? Comparing European expatriate adjustment in China and Turkey. *Journal of International Management* **12**, 449-472 (2006).

902. Ward, C. & Kennedy, A. Locus of control, mood disturbance, and social difficulty during cross-cultural transitions. *International journal of intercultural relations* **16**, 175-194 (1992).

903. Ward, C. & Kennedy, A. Psychological and Socio-cultural Adjustment during Cross-cultural Transitions: A Comparison of Secondary Students Overseas and at Home. *International Journal of Psychology* **28**, 129-147 (1993).

904. Ward, C. & Kennedy, A. Where's the "Culture" in Cross-Cultural Transition?: Comparative Studies of Sojourner Adjustment. *Journal of Cross-Cultural Psychology* **24**, 221-249 (1993).

905. Ward, C. & Rana-Deuba, A. Home and host culture influences on sojourner adjustment. *International Journal of Intercultural Relations* **24**, 291-306 (2000).

906. Ward, C. & Searle, W. The impact of value discrepancies and cultural identity on psychological and sociocultural adjustment of sojourners. *International Journal of Intercultural Relations* **15**, 209-224 (1991).

907. Ward, C., Stuart, J. & Kus, L. The Construction and Validation of a Measure of Ethno-Cultural Identity

Conflict. *Journal of Personality Assessment* **93**, 462-473 (2011).

908. Waxin, M. F. Expatriates' interaction adjustment: the direct and moderator effects of culture of origin. *International Journal of Intercultural Relations* **28**, 61-79 (2004).

909. Wechtler, H., Koveshnikov, A. & Dejoux, C. Just like a fine wine? Age, emotional intelligence, and cross-cultural adjustment. *International Business Review* **24**, 409-418 (2015).

910. Wei, M., Ku, T.-Y., Russell, D. W., Mallinckrodt, B. & Liao, K. Y.-H. Moderating Effects of Three Coping Strategies and Self-Esteem on Perceived Discrimination and Depressive Symptoms: A Minority Stress Model for Asian International Students. *Journal of Counseling Psychology* **55**, 451-462 (2008).

911. Wei, M., Liang, Y. S., Du, Y., Botello, R. & Li, C. I. Moderating effects of perceived language discrimination on mental health outcomes among Chinese international students. *Asian American Journal of Psychology* **6**, 213-222 (2015).

912. Wei, M., Tsai, P.-C., Chao, R., Du, Y. & Lin, S.-P. Advisory Working Alliance, Perceived English Proficiency, and Acculturative Stress. *Journal of Counseling Psychology* **59**, 437-448 (2012).

913. Wei, M., Wang, K. T., Heppner, P. P. & Du, Y. Ethnic and Mainstream Social Connectedness, Perceived Racial Discrimination, and Posttraumatic Stress Symptoms. *Journal of Counseling Psychology* **59**, 486-493 (2012).

914. Weivoda, L. J. The relationship between cultural distance and expatriate adjustment to host countries. (ProQuest Dissertations and Theses, 2016).

915. Wen, W., Luo, Y. & Hu, D. Bridging the Gap between Western and Chinese Evidence in the International Education Market. *China & World Economy* **22**, 87-102 (2014).

916. Werkuyten, M. & Nekuee, S. Subjective Well-Being, Discrimination and Cultural Conflict: Iranians Living in The Netherlands. *Social Indicators Research* **47**, 281-306 (1999).

917. Whitehead, M. R. Exploring changes among parental acculturation, immigration-related stress, parental depression, and parental alliance: A longitudinal study with a sample of Latino|a immigrant parents. (ProQuest Dissertations and Theses, 2016).

918. Wiese, D. Psychological Health of Expatriate Spouses: A Neglected Factor in International Relocation.

Asian Journal of Counselling (2013).

919. Williams, M. S. Acculturation and immigrants: The moderating effect of cultural identity salience and harmony enhancement and the mediating effect of social support and ostracism on the relationship between acculturation demands and employee outcomes. (ProQuest Dissertations and Theses, 2017).

920. Wilson, J., Ward, C., Fetvadjiev, V. H. & Bethel, A. Measuring Cultural Competencies: The Development and Validation of a Revised Measure of Sociocultural Adaptation. *Journal of Cross-Cultural Psychology* **48**, 1475-1506 (2017).

921. Winn, L. Relations between depression, acculturation, enculturation and alcohol use among recently immigrated Latina young adults. (ProQuest Dissertations and Theses, 2020).

922. Won, S. & Kim, H. Importance of Family Values Differences Between Husbands and Wives in Determining Depression in Foreign Wives in Korean Multicultural Families: Examining the Moderating Effect of Social Support. *Asian Social Work and Policy Review* **8**, 1-15 (2014).

923. Wong, C. W., Schweitzer, R. D. & Khawaja, N. G. Individual, Pre-Migration, and Post-Settlement Factors in Predicting Academic Success of Adolescents from Refugee Backgrounds: a 12-Month Follow-Up. *Journal of International Migration and Integration* **19**, 1095-1117 (2018).

924. Wong, W. K., Ng, I. F. & Chou, K. L. Factors contributing to social support among female marriage migrants in Hong Kong: A longitudinal study. *International Social Work* **60**, 394-408 (2016).

925. Wong, Y. J., Tsai, P. C., Liu, T., Zhu, Q. & Wei, M. Male Asian international students' perceived racial discrimination, masculine identity, and subjective masculinity stress: a moderated mediation model. *Journal of Counseling Psychology* **61**, 560-569 (2014).

926. Wood, E. D. Post-arrival performance interventions that assist expatriates' adjustment and performance. (ProQuest Dissertations and Theses, 2010).

927. Wu, E. K. Y. & Mak, W. W. S. Acculturation Process and Distress: Mediating Roles of Sociocultural Adaptation and Acculturative Stress. *The Counseling Psychologist* **40**, 66-92 (2011).

928. Wu, J., Yang, J., McIntyre, J. R. & Zhang, X. Revisiting the influence of cultural novelty and emotional stability on general adjustment of expatriates hosted in emerging economies. *Cross Cultural & Strategic*

Management **29**, 870-898 (2022).

929. Wu, P.-C. & Ang, S. H. The impact of expatriate supporting practices and cultural intelligence on cross-cultural adjustment and performance of expatriates in Singapore. *The International Journal of Human Resource Management* **22**, 2683-2702 (2011).

930. Wu, S., Fan, D. & Dabasia, A. J. Expatriate adjustment and subsidiary performance: a motivation-hygiene perspective. *International Journal of Manpower* **44**, 1-18 (2022).

931. Wu, S., *et al.* Time-varying associations of pre-migration and post-migration stressors in refugees' mental health during resettlement: a longitudinal study in Australia. *Lancet Psychiatry* **8**, 36-47 (2021).

932. Wypych, M. & Bilewicz, M. Psychological toll of hate speech: The role of acculturation stress in the effects of exposure to ethnic slurs on mental health among Ukrainian immigrants in Poland. *Cultural Diversity and Ethnic Minority Psychology* **30**, 35-44 (2022).

933. Xia, M. & Duan, C. Stress Coping of Chinese International Students in Face of COVID 19 Pandemic: Cultural Characteristics. *International Journal of Mental Health Promotion* **22**, 159-172 (2020).

934. Xia, M., Yang, C., Zhou, Y., Cheng, G. & Yu, J. One belt & one road international students' gratitude and acculturation stress: A moderated mediation model. *Current Psychology* **42**, 1212-1224 (2021).

935. Xiao, H. T. Chinese and Korean student sociocultural adaptation and intercultural organizational involvement at a selected private university. (ProQuest Dissertations and Theses, 2015).

936. Xiao, S. L., Yang, Z. X. & Bernardo, A. B. I. Polyculturalism and cultural adjustment of international students: Exploring the moderating role of cultural distance in a quantitative cross-sectional survey study. *Cogent Psychology* **6**(2019).

937. Xie, M. A mixed-method study of friendship networks and psychological and sociocultural adaptation among Chinese international undergraduates in U.S. higher education. (ProQuest Dissertations and Theses, 2019).

938. Xinyi, J. On cultural identity, acculturation strategy and social support of Chinese international students in the U.S. (Shanghai International Studies University, 2016).

939. Xiong, Y. An exploration of Asian international students' mental health: Comparisons to American

students and other international students in the United States. (ProQuest Dissertations and Theses, 2018).

940. Xu, T. Psychological Distress of International Students during the COVID-19 Pandemic in China: Multidimensional Effects of External Environment, Individuals' Behavior, and Their Values. *International journal of environmental research and public health* **18**(2021).

941. Xue, X., Cheah, C. S. L. & Hart, C. H. Risk and protective processes in the link between racial discrimination and Chinese American mothers' psychologically controlling parenting. *Cultural Diversity and Ethnic Minority Psychology* **30**, 143-155 (2022).

942. Yakobov, E., Jurcik, T., Solopieieva-Jurcikova, I. & Ryder, A. G. Expectations and acculturation: Further unpacking of adjustment mechanisms within the Russian-speaking community in Montreal. *International Journal of Intercultural Relations* **68**, 67-76 (2019).

943. Yakunina, E. S., Weigold, I. K., Weigold, A., Hercegovac, S. & Elsayed, N. International Students' Personal and Multicultural Strengths: Reducing Acculturative Stress and Promoting Adjustment. *Journal of Counseling & Development* **91**, 216-223 (2013).

944. Yalim, A. C. The Impacts of Contextual Factors on Psychosocial Wellbeing of Syrian Refugees: Findings from Turkey and the United States. *Journal of Social Service Research* **47**, 104-117 (2020).

945. Yamei, H. The acculturation strategy, acculturative stress and adaptation outcomes of expatriates working in some foreign enterprize in Shanghai. (Shanghai International Studies University, 2011).

946. Yan, Y. Facebook and Wechat: Chinese international students' social media usage and how it influences their process of intercultural adaptation. (ProQuest Dissertations and Theses, 2018).

947. Yanardağ, M. Z., Budak, F. & Özer, Ö. Psychological flourishing and social support in young Syrian adults: a study on psychosocial health. *Journal of Human Behavior in the Social Environment* **32**, 501-518 (2021).

948. Yang, B. & Clum, G. A. Measures of life stress and social support specific to an Asian student population. *Journal of Psychopathology and Behavioral Assessment* **17**, 51-67 (1995).

949. Yang, F., He, Y. & Xia, Z. The effect of perceived discrimination on cross-cultural adaptation of international students: moderating roles of autonomous orientation and integration strategy. *Current*

Psychology **42**, 19927-19940 (2022).

950. Yang, F.-J. The "How" Question of the Healthy Immigrant Paradox: Understanding Psychosocial Resources and Demands as Pathways Linking Migration to Mental Health Risks. *Society and Mental Health* **11**, 69-89 (2020).

951. Yang, H. J., Wu, J. Y., Huang, S. S., Lien, M. H. & Lee, T. S. Perceived discrimination, family functioning, and depressive symptoms among immigrant women in Taiwan. *Archives of women's mental health* **17**, 359-366 (2014).

952. Yang, M. S. The impacts of life events on depression in later life among older Hmong immigrants. (ProQuest Dissertations and Theses, 2017).

953. Yang, N.*, et al.* Acculturative stress, poor mental health and condom-use intention among international students in China. *Health Education Journal* **77**, 142-155 (2017).

954. Yang, R. P.-J., Noels, K. A. & Saumure, K. D. Multiple routes to cross-cultural adaptation for international students: Mapping the paths between self-construals, English language confidence, and adjustment. *International Journal of Intercultural Relations* **30**, 487-506 (2006).

955. Ye, J. Acculturative Stress and Use of the Internet among East Asian International Students in the United States. *Cyberpsychology & Behavior* **8**, 154-161 (2005).

956. Ye, J. An Examination of Acculturative Stress, Interpersonal Social Support, and Use of Online Ethnic Social Groups among Chinese International;Students. *The Howard Journal of Communications* **17**, 1-20 (2006).

957. Ye, J. Traditional and Online Support Networks in the Cross-Cultural Adaptation of Chinese International Students in the United States. *Journal of Computer-Mediated Communication* **11**, 863-876 (2006).

958. Yeung, N. C. Y., Kan, K. K. Y., Wong, A. L. Y. & Lau, J. T. F. Self-stigma, resilience, perceived quality of social relationships, and psychological distress among Filipina domestic helpers in Hong Kong: A mediation model. *Stigma and Health* **6**, 90-99 (2021).

959. Yeung, W. J. J. & Mu, Z. Happiness of female immigrants in cross-border marriages in Taiwan. *Journal of Ethnic and Migration Studies* **46**, 2956-2976 (2019).

960. Yi, F. Acculturative stress, meaning-in-life, collectivistic coping, and subjective well-being among Chinese international students: A moderated mediation model. (ProQuest Dissertations and Theses, 2018).

961. Yildirim, M., Aziz, I. A., Vostanis, P. & Hassan, M. N. Associations among resilience, hope, social support, feeling belongingness, satisfaction with life, and flourishing among Syrian minority refugees. *Journal of Ethnicity in Substance Abuse* **23**, 1-16 (2022).

962. Yim, H., Kim, A. C. H., Du, J. & James, J. D. Sport participation, acculturative stress, and depressive symptoms among international college students in the United States. (ProQuest Dissertations and Theses, 2020).

963. Yin, M., Aoki, K., Liao, K. Y.-H. & Xu, H. An Exploration on the Attachment, Acculturation, and Psychosocial Adjustment of Chinese International Students in Japan. *Journal of International Students* **11**, 176-194 (2021).

964. Ying, Y.-W. Variation in acculturative stressors over time: A study of Taiwanese students in the United States. *International Journal of Intercultural Relations* **29**, 59-71 (2005).

965. Ying, Y.-W. & Han, M. The contribution of personality, acculturative stressors, and social affiliation to adjustment: A longitudinal study of Taiwanese students in the United States. *International Journal of Intercultural Relations* **30**, 623-635 (2006).

966. Ying, Y.-W. & Han, M. Variation in the Prediction of Cross-Cultural Adjustment by Ethnic Density: A Longitudinal Study of Taiwanese Students in the United States. *College Student Journal* **42**, 1075-1086 (2008).

967. Ying, Y.-W. & Liese, L. H. Emotional well-being of Taiwan students in the U.S.: An examination of pre- to post-arrival differential. *International Journal of Intercultural Relations* **15**, 345-366 (1991).

968. Ying, Y. W. & Liese, L. H. Initial Adaptation of Taiwan Foreign Students to the United States: The Impact of Prearrivai Variables. *American Journal of Community Psycholog* **18**, 825-845 (1990).

969. Yılmaz, K. & Temizkan, V. The Effects of Educational Service Quality and Socio-Cultural Adaptation Difficulties on International Students' Higher Education Satisfaction. *SAGE Open* **12**(2022).

970. Yoon, E., Hacker, J., Hewitt, A., Abrams, M. & Cleary, S. Social Connectedness, Discrimination, and

Social Status as Mediators of Acculturation/Enculturation and Well-Being. *Journal of Counseling Psychology* **59**, 86-96 (2011).

971. Yoon, J. Purpose development, acculturation, and identity among South Sudanese unaccompanied refugee minors: A multimethod analysis of longitudinal adjustment outcomes. (ProQuest Dissertations and Theses, 2018).

972. Yoon, Y. Psychological and sociocultural adaptation of international students through sport spectatorship: A mixed method approach. (ProQuest Dissertations and Theses, 2015).

973. Young, M. T. & Pebley, A. R. Legal Status, Time in the USA, and the Well-Being of Latinos in Los Angeles. *Journal of Urban Health* **94**, 764-775 (2017).

974. Young, T. J., Sercombe, P. G., Sachdev, I., Naeb, R. & Schartner, A. Success factors for international postgraduate students' adjustment: exploring the roles of intercultural competence, language proficiency, social contact and social support. *European Journal of Higher Education* **3**, 151-171 (2013).

975. Yu, B., Bodycott, P. & Mak, A. S. Language and Interpersonal Resource Predictors of Psychological and Sociocultural Adaptation: International Students in Hong Kong. *Journal of Studies in International Education* **23**, 572-588 (2019).

976. Yu, B., Mak, A. S. & Bodycott, P. Psychological and academic adaptation of mainland Chinese students in Hong Kong universities. *Studies in Higher Education* **46**, 1552-1564 (2019).

977. Yu, B. & Shen, H. Predicting roles of linguistic confidence, integrative motivation and second language proficiency on cross-cultural adaptation. *International Journal of Intercultural Relations* **36**, 72-82 (2012).

978. Yu, L., Renzaho, A. M. N., Shi, L., Ling, L. & Chen, W. The Effects of Family Financial Stress and Primary Caregivers' Levels of Acculturation on Children's Emotional and Behavioral Problems among Humanitarian Refugees in Australia. *International journal of environmental research and public health* **17**(2020).

979. Yu, X., Stewart, S. M., Liu, I. K. & Lam, T. H. Resilience and depressive symptoms in mainland Chinese immigrants to Hong Kong. *Social Psychiatry and Psychiatric Epidemiology* **49**, 241-249 (2014).

980. Yuan, C. T. P. The influences of dual social network site use and social capital development on sociocultural adaptation. (ProQuest Dissertations and Theses, 2015).

981. Yue, X. Chinese international students' social support and intercultural communication apprehension in the context of intercultural adaptation. (Shanghai International Studies University, 2016).

982. Yun, C. T. P. & Greenwood, K. M. Stress, Sleep and Performance in International and Domestic University Students. *Journal of International Students* **12**, 81-100 (2021).

983. Yusoff, Y. Self-Efficacy, Perceived;Social Support, and;Psychological Adjustment in;International Undergraduate;Students in a Public Higher;Education Institution in;Malaysia. *Journal of Studies in International Education* **16**, 353-371 (2012).

984. Zakariyaa, N. H., Othmanb, A. K. & Abdullahb, Z. The relationship between expatriate adjustment and expatriate job performance at multinational corporations in Malaysia. *Academy of Entrepreneurship Journal* **25.0**, 1-11 (2019).

985. Zeng, F., *et al.* Biopsychosocial Factors Associated with Depression among U.S. Undergraduate International Students. *Journal of International Students* **12**, 101-122 (2021).

986. Zghal, A. Exploring the impact of patient perceptions of health care provider cultural competence on health-related quality of life among an immigrant population. (ProQuest Dissertations and Theses, 2018).

987. Zghal, A., El-Masri, M., McMurphy, S. & Pfaff, K. Exploring the Impact of Health Care Provider Cultural Competence on New Immigrant Health-Related Quality of Life: A Cross-Sectional Study of Canadian Newcomers. *Journal of Transcultural Nursing* **32**, 508-517 (2021).

988. Zhang, J. & Goodson, P. Acculturation and psychosocial adjustment of Chinese international students: Examining mediation and moderation effects. *International Journal of Intercultural Relations* **35**, 614-627 (2011).

989. Zhang, J., Smith, S., Swisher, M., Fu, D. & Fogarty, K. Gender Role Disruption and Marital Satisfaction among Wives of Chinese International Students in the United States. *Journal of Comparative Family Studies* **42**, 523-542 (2011).

990. Zhang, K., Wu, B. & Zhang, W. Perceived Neighborhood Conditions, Self-Management Abilities, and

Psychological Well-Being Among Chinese Older Adults in Hawai'i. *Journal of Applied Gerontology* **41**, 1111-1119 (2022).

991. Zhang, W., Liu, S., Zhang, K. & Wu, B. Neighborhood Social Cohesion, Resilience, and Psychological Well-Being Among Chinese Older Adults in Hawai'i. *Gerontologist* **60**, 229-238 (2020).

992. Zhang, X. Acculturation and acculturation gaps: Impacts on the educational involvement and psychological adjustment of Chinese immigrant parents. (ProQuest Dissertations and Theses, 2021).

993. Zhang, X. & Kong, P. A. Immigrant Chinese parents in New York Chinatowns: Acculturation gap and psychological adjustment. *Asian American Journal of Psychology* **14**, 145-154 (2023).

994. Zhang, Y. & Jung, E. Multi-Dimensionality of Acculturative Stress among Chinese International Students: What Lies behind Their Struggles? *International Research and Review* **7.0**, 23-43 (2017).

995. Zhang, Y*., et al.* The impact of organizational position level and cultural flow direction on the relationship between cultural intelligence and expatriate cross-border adaptation. *Cross Cultural & Strategic Management* **28**, 332-367 (2021).

996. Zhang, Y. & Oczkowski, E. Exploring the potential effects of expatriate adjustment direction. *Cross Cultural & Strategic Management* **23**, 158-183 (2016).

997. Zhou, X. & Qin, J. A Study on Cross-Cultural Adjustment of Japanese and American Expatriates in China. *International Journal of Business and Management* **4**(2009).

998. Zhu, J., Wanberg, C. R., Harrison, D.A. & Diehn, E.W. Ups and downs of the expatriate experience? Understanding work adjustment trajectories and career outcomes. *Journal of Applied Psychology* **101**, 549-568 (2016).

999. Zhu, L., Liu, M. & Fink, E. L. The Role of Person-Culture Fit in Chinese Students' Cultural Adjustment in the United States: A Galileo Mental Model Approach. *Human Communication Research* **42**, 485-505 (2016).

1000. Zhuang, W. L., Wu, M. & Wen, S. L. Relationship of mentoring functions to expatriate adjustments: comparing home country mentorship and host country mentorship. *The International Journal of Human Resource Management* **24**, 35-49 (2013).

1001. Zimmermann, S. Perceptions of intercultural communication competence and international student adaptation to an American campus. *Communication Education* **44**, 321-335 (1995).

1002. Zlobina, A., Basabe, N., Paez, D. & Furnham, A. Sociocultural adjustment of immigrants: Universal and group-specific predictors. *International Journal of Intercultural Relations* **30**, 195-211 (2006).

1003. Zlotnick, C., Dryjanska, L. & Suckerman, S. The Association Between Acculturation Variables and Life Satisfaction Among Israeli Immigrants from Four English-Speaking Countries. *Journal of Happiness Studies* **21**, 1427-1444 (2019).

1004. Zlotnick, C., Dryjanska, L. & Suckerman, S. Health literacy, resilience and perceived stress of migrants in Israel during the COVID-19 pandemic. *Psychology & health* **37**, 1076-1092 (2022).

1005. Zou, X., Hall, B. J., Xiong, M. & Wang, C. Post-migration well-being of Sub-Saharan Africans in China: a nationwide cross-sectional survey. *Quality of Life Research* **30**, 1025-1035 (2021).

References for Supplementary Materials

1006. Bakhtiari, F., Benner, A. D. & Plunkett, S. W. Life quality of university students from immigrant families in the United States. *Fam. Consum. Sci. Res. J.* **46**, 331–346 (2018).

1007. Gokuladas, V. K. & Baby Sam, S. K. Student satisfaction in secondary education: An empirical study of Indian expatriate students. *J. Res. Int. Educ.* **21**, 73–83 (2022).

1008. Gomes, C. Outside the classroom: The language of English and its impact on international student mental health. *J. Int. Stud.* **10**, 934–953 (2020).

1009. Bierwiaczonek, K. *Cross-cultural adaptation as an intergroup phenomenon: antecedents, processes and manifestations* (unpublished doctoral dissertation, VU, 2018).

1010. Bierwiaczonek, K. & Waldzus, S. Socio-cultural factors as antecedents of cross-cultural adaptation in expatriates, international students, and migrants: A review. *J. Cross-Cult. Psychol.* **47**, 767–817 (2016).

1011. Bak-Klimek, A., Karatzias, T., Elliott, L. & Maclean, R. The determinants of well-being among international economic immigrants: A systematic literature review and meta-analysis. *Appl. Res. Qual. Life* **10**, 161–188 (2015).

1012. Bender, M., van Osch, Y., Sleegers, W. & Ye, M. Social support benefits psychological adjustment of international students: Evidence from a meta-analysis. *J. Cross-Cult. Psychol.* **50**, 827–847 (2019).

1013. Bhaskar-Shrinivas, P., Harrison, D. A., Shaffer, M. A. & Luk, D. Input-based and time-based models of international adjustment: Meta-analytic evidence and theoretical extensions. *Acad. Manag. J.* **48**, 257–281 (2005).

1014. Wilson, J., Ward, C. & Fischer, R. Beyond culture learning theory: What can personality tell us about cultural competence? *J. Cross-Cult. Psychol.* **44**, 900–927 (2013). https://doi.org/10.1177/0022022113492889

1015. Dimitrova, R., Chasiotis, A. & van de Vijver, F. Adjustment outcomes of immigrant children and youth in Europe: A meta-analysis. *Eur. Psychol.* **21**, 150–162 (2016). https://doi.org/10.1027/1016-9040/a000246

1016. Hechanova, R., Beehr, T. A. & Christiansen, N. D. Antecedents and consequences of employees' adjustment to overseas assignment: A meta-analytic review. *Appl. Psychol.* **52**, 213–236 (2003). https://doi.org/10.1111/1464-0597.00132

1017. van der Laken, P. A., Van Engen, M. L., Van Veldhoven, M. J. P. M. & Paauwe, J. Fostering expatriate success: A meta-analysis of the differential benefits of social support. *Hum. Resour. Manag. Rev.* **29**, 100679 (2019).

1018. van de Schoot, R. *et al.* An open source machine learning framework for efficient and transparent systematic reviews. *Nat. Mach. Intell.* **3**, 125–133 (2021).

1019. Moola, S. *et al.* Systematic reviews of etiology and risk. In *JBI Manual for Evidence Synthesis* (eds Aromataris, E. & Munn, Z.) (JBI, 2020). https://synthesismanual.jbi.global

1020. Lipsey, M. W. & Wilson, D. B. *Practical meta-analysis* (Sage Publications, 2001).

1021. Cohen, J., Cohen, P., West, S. G. & Aiken, L. S. *Applied multiple regression/correlation analysis for the behavioral sciences* (Routledge, 2013).

1022. Jacobs, P. & Viechtbauer, W. Estimation of the biserial correlation and its sampling variance for use in meta-analysis. *Res. Synth. Methods* **8**, 161–180 (2017).

1023. Pustejovsky, J. E. Converting from d to r to z when the design uses extreme groups, dichotomization, or experimental control. *Psychol. Methods* **19**, 92–99 (2014).

1024. Viechtbauer, W. Package 'metafor'. *Comprehensive R Archive Network* (2015).

1025. Vu, D. H., Kunst, J. R., Tong, R. & Bierwiaczonek, K. Methodological challenges in cross-cultural adaptation research: Insights from a large-scale meta-analysis. In *Handbook of Communication and Culture* (ed. Arasaratnam-Smith, L.) (Edward Elgar Publishing, in press).

1026. Pustejovsky, J. E. & Rodgers, M. A. Testing for funnel plot asymmetry of standardized mean differences. *Res. Synth. Methods* **10**, 57–71 (2019). https://doi.org/10.1002/jrsm.1332

1027. Stanley, T. D. & Doucouliagos, H. Meta-regression approximations to reduce publication selection bias. *Res. Synth. Methods* **5**, 60–78 (2014).

1026. Mathur, M. B. P-hacking in meta-analyses: A formalization and new meta-analytic methods. *Res. Synth. Methods* **15**, 483–499 (2024). https://doi.org/10.1002/jrsm.1701

1027. Page, M.J, McKenzie, J.E., Bossuyt, P.M., Boutron, I., Hoffmann, T.C., Mulrow, C.D., et al. The PRISMA 2020 statement: an updated guideline for reporting systematic reviews. *BMJ* **372**, n71 (2021). https:/doi.org/10.1136/bmj.n71
