# Supplementary material for: A Meta-Analysis of Social and Contextual Correlates of Migrant Adaptation to Living in Receiving Societies
Source: Nat Commun. 2025 Dec 17;16:11231. doi: 10.1038/s41467-025-67468-z (PMC12715197; doi:10.1038/s41467-025-67468-z)
Supplement: Supplementary file 2 — Reporting Summary [file 41467_2025_67468_MOESM2_ESM.pdf]

nature portfolio

Corresponding author(s): Kinga Bierwiaczonek

Last updated by author(s): Oct 15, 2025

Reporting Summary

Nature Portfolio wishes to improve the reproducibility of the work that we publish. This form provides structure for consistency and transparency in reporting. For further information on Nature Portfolio policies, see our Editorial Policies and the Editorial Policy Checklist.

Statistics

For all statistical analyses, confirm that the following items are present in the figure legend, table legend, main text, or Methods section.

n/a	Confirmed	
☐	☒	The exact sample size (*n*) for each experimental group/condition, given as a discrete number and unit of measurement
☒	☐	A statement on whether measurements were taken from distinct samples or whether the same sample was measured repeatedly
☐	☒	The statistical test(s) used AND whether they are one- or two-sided<br>*Only common tests should be described solely by name; describe more complex techniques in the Methods section.*
☒	☐	A description of all covariates tested
☐	☒	A description of any assumptions or corrections, such as tests of normality and adjustment for multiple comparisons
☐	☒	A full description of the statistical parameters including central tendency (e.g. means) or other basic estimates (e.g. regression coefficient) AND variation (e.g. standard deviation) or associated estimates of uncertainty (e.g. confidence intervals)
☐	☒	For null hypothesis testing, the test statistic (e.g. *F*, *t*, *r*) with confidence intervals, effect sizes, degrees of freedom and *P* value noted<br>*Give P values as exact values whenever suitable.*
☒	☐	For Bayesian analysis, information on the choice of priors and Markov chain Monte Carlo settings
☐	☒	For hierarchical and complex designs, identification of the appropriate level for tests and full reporting of outcomes
☐	☒	Estimates of effect sizes (e.g. Cohen's *d*, Pearson's *r*), indicating how they were calculated

Our web collection on statistics for biologists contains articles on many of the points above.

Software and code

Policy information about availability of computer code

Data collection	ASReview was used for abstract screening. No other software was used.
Data analysis	Analysis: metafor 4.6 package for R; data visualization: mapdata v.2.3.1 (Figure 1) and ggplot2 v.3.5.2(Figures 2-8)

For manuscripts utilizing custom algorithms or software that are central to the research but not yet described in published literature, software must be made available to editors and reviewers. We strongly encourage code deposition in a community repository (e.g. GitHub). See the Nature Portfolio guidelines for submitting code & software for further information.

Data

Policy information about availability of data

All manuscripts must include a data availability statement. This statement should provide the following information, where applicable:

- Accession codes, unique identifiers, or web links for publicly available datasets
- A description of any restrictions on data availability
- For clinical datasets or third party data, please ensure that the statement adheres to our policy

The data and code generated in this study have been deposited in the Open Science Framework repository under accession code https://doi.org/10.17605/OSF.IO/MD3SZ

Research involving human participants, their data, or biological material

Policy information about studies with human participants or human data. See also policy information about sex, gender (identity/presentation), and sexual orientation and race, ethnicity and racism.

Reporting on sex and gender	Sample socio-demographics, including gender, are reported in Table 1. Sample-level percentage of male participants, as reported by authors of the primary studies, was used as predictor in an additional analyses (Supplementary Table 7).
Reporting on race, ethnicity, or other socially relevant groupings	Sample ethnicity was reported in Table 1 and Figure 1.
Population characteristics	Sample characteristics were reported in Table 1.
Recruitment	This study did not involve participant recruitment.
Ethics oversight	This study did not collect primary data, it was therefore exempted from ethical approval.

Note that full information on the approval of the study protocol must also be provided in the manuscript.

Field-specific reporting

Please select the one below that is the best fit for your research. If you are not sure, read the appropriate sections before making your selection.

☐ Life sciences ☒ Behavioural & social sciences ☐ Ecological, evolutionary & environmental sciences

For a reference copy of the document with all sections, see nature.com/documents/nr-reporting-summary-flat.pdf

Behavioural & social sciences study design

All studies must disclose on these points even when the disclosure is negative.

Study description	This study is a quantitative multilevel meta-analysis of observational studies on the correlates of migrant cross-cultural adaptation, including multilevel meta-regressions used to determine the relative strength of the different correlates.
Research sample	This study aimed at collecting data from all available primary studies on social and contextual correlates of migrant adaptation belonging to four groups: stressors, social resources, exposure to the receiving society, cultural distance. The dataset consisted of data from 1,114 primary studies and 5,066 effects on migrant adaptation, covering 571,260 first-generation migrant participants arriving from 73 countries and residing in 64 countries. Depending on correlate category, gender composition of primary samples ranged, on average, from 46.02% to 60.13% of male participants, and the average age ranged from 30.15% to 35.48%. The representativeness of the sample varied between primary studies.
Sampling strategy	To be eligible for this study, a primary study needed to fulfill the following criteria: (1) use a quantitative design; (2) be conducted no earlier than 1988 (Black's study is considered the first study with a theoretical framing fitting this meta-analysis); (3) be available in English; (4) include an eligible sample consisting of participants in international mobility; (5) include at least one eligible measure of cross-cultural adaptation; (6) include at least one eligible measure of correlates of adaptation (for a list, see Supplementary Table 12), (7) report at least one correlation between a measure of adaptation and a measure of adaptation antecedent or other statistical information sufficient to estimate at least one such correlation.
Data collection	Literature search followed the PRISMA guidelines and is summarized in Figure 9. Studies dating from the period 1988-2014 were included from an existing database by Bierwiaczonek. Studies dating from 2014 or later were identified using a new literature search. All hits from the databases covered by the core literature search (k = 16,639) were screened using ASReview Lab program. Three members of the project team (one holding a PhD in psychology, two holding master's degrees) took turns reviewing titles and abstracts. All the abstracts marked as irrelevant were then checked independently by the fourth team member (holding a master's degree). All discrepancies were discussed and resolved by the team. Full texts of the retained records were screened and coded by nine trained members of the project team (either holding a master's degree or master students). Each full text was screened and coded independently by two team members, resulting in a 77.29% agreement. Any discrepancies or difficulties in the coding process were discussed with the first author and resolved in weekly project team meetings. Additionally, any outliers were checked by three members of the coder team prior to running the analyses.
Timing	Data collection (literature search) was conducted on June 20, 2022 and June 24, 2022 . The included studies cover the period from 1988 to 2022.
Data exclusions	All studies that fulfilled the inclusion criteria were included.
Non-participation	Not applicable
Randomization	Not applicable

Reporting for specific materials, systems and methods

We require information from authors about some types of materials, experimental systems and methods used in many studies. Here, indicate whether each material, system or method listed is relevant to your study. If you are not sure if a list item applies to your research, read the appropriate section before selecting a response.

Materials & experimental systems

n/a	Involved in the study
☒	☐ Antibodies
☒	☐ Eukaryotic cell lines
☒	☐ Palaeontology and archaeology
☒	☐ Animals and other organisms
☒	☐ Clinical data
☒	☐ Dual use research of concern
☒	☐ Plants

Methods

n/a	Involved in the study
☒	☐ ChIP-seq
☒	☐ Flow cytometry
☒	☐ MRI-based neuroimaging

Plants

Seed stocks	NA
Novel plant genotypes	NA
Authentication	NA
